# Estrogen-related receptor alpha (ERRα) controls the stemness and cellular energetics of prostate cancer cells via its direct regulation of citrate metabolism and zinc transportation

**DOI:** 10.1038/s41419-025-07460-z

**Published:** 2025-03-05

**Authors:** Taiyang Ma, Wenjuan Xie, Zhenyu Xu, Weijie Gao, Jianfu Zhou, Yuliang Wang, Franky Leung Chan

**Affiliations:** 1 School of Biomedical Sciences, The Chinese University of Hong Kong, Shatin, Hong Kong China; 2 Department of Surgery, Faculty of Medicine, The Chinese University of Hong Kong, Shatin, Hong Kong China; 3 Department of Urology, The Second Affiliated Hospital of Guangzhou University of Chinese Medicine, Guangzhou, China

**Keywords:** Prostate cancer, Cancer stem cells

## Abstract

Compared to most tumors that are more glycolytic, primary prostate cancer is less glycolytic but more dependent on TCA cycle coupled with OXPHOS for its energy demand. This unique metabolic energetic feature is attributed to activation of mitochondrial m-aconitase in TCA caused by decreased cellular Zn level. Evidence suggests that a small subpopulation of cancer cells within prostate tumors, designated as prostate cancer stem cells (PCSCs), play significant roles in advanced prostate cancer progression. However, their cellular energetics status is still poorly understood. Nuclear receptor ERRα (*ESRRA*) is a key regulator of energy metabolism. Previous studies characterize that ERRα exhibits an upregulation in prostate cancer and can perform multiple oncogenic functions. Here, we demonstrate a novel role of ERRα in the control of stemness and energetics metabolism in PCSCs via a mechanism of combined transrepression of Zn transporter *ZIP1* in reducing intracellular Zn uptake and transactivation of *ACO2* (m-aconitase) in completion of TCA cycle. Results also showed that restoration of Zn accumulation by treatment with a Zn ionophore Clioquinol could significantly suppress both in vitro growth of PCSCs and also their in vivo tumorigenicity, implicating that enhanced cellular Zn uptake could be a potential therapeutic approach for targeting PCSCs in advanced prostate cancer.

## Introduction

Energy metabolism reprogramming or alteration is generally recognized as a hallmark in cancer as being an adaptation to nutrient deficiency, microenvironmental changes and therapies [1]. It is generally accepted that the rapidly proliferating cancer cells in most solid cancers rely on the aerobic glycolysis for considerably less effective but rapid ATP production even provided with sufficient oxygen (Warburg effect phenomenon), instead of relying on the complete oxidation of glucose by mitochondrial oxidative phosphorylation (OXPHOS) due to impaired mitochondrial respiration [2] or as adaptive metabolic requirements caused by oncogenic mutations [3, 4]. However, contrary to Warburg’s original hypothesis, accumulating evidence reveals that cancer cells exhibiting Warburg metabolism have intact mitochondrial respiration, and mitochondrial metabolism is essential for tumor formation and progression [5]. Moreover, tissue-of-origin plays a critical role in defining the metabolic reprogramming that sustains tumor growth [6]. In contrast to most other solid tumors, primary prostate cancer is revealed as exhibiting less glycolytic but more dependent on tricarboxylic acid (TCA) cycle coupled with OXPHOS, as compared to the normal prostate epithelial cells which are more glycolytic [7, 8]. This metabolic shift to more OXPHOS in primary prostate cancer is related to the activation of m-aconitase in TCA resulted by a decrease of mitochondrial Zn level. However, transcriptional regulation of this metabolic shift or reprogramming during prostate cancer development and progression is still not well understood.

Accumulating experimental and clinical evidence indicates that a small subpopulation of cancer cells within tumors or hematological cancers, named as cancer stem cells (CSCs; also tumor progenitor or initiating cells), as being characterized by their displayed phenotypic and functional properties commonly shared with normal tissue stem cells such as self-renewal and differentiation capacities, cancer drug resistance, high tumorigenicity in vivo and expression of certain common stem cell markers [9], can contribute significantly to cancer recurrence and advanced malignant growth. Prostate cancer stem cells (PCSCs) are confirmed to be present in prostate cancer cell lines and patient-derived prostate tumor tissues. Indeed, many experimental studies show that these PCSCs can contribute to the therapy-resistance and metastatic growth [10, 11].

Studies show that CSCs isolated from different cancer types and sources exhibit different preferred metabolic profiles for their energy production. CSCs derived from some patients-derived tumors or transgene-induced tumors, including glioblastoma [12] and pancreatic cancers [13], are shown to rely more on mitochondrial respiration but less on glycolysis for their energy demands. However, CSCs isolated from different cancer types-derived cancer cell lines or primary cultures, including breast [14], lung cancer [15], and ovarian cancers [16], are shown to rely more on glycolysis than OXPHOS. On the other hand, the status of cellular energetics in PCSCs is still unclear and undefined. One study shows that HectH9 E3 ligase can play a key role in glucose metabolism regulation in prostate cancer via its control on the ubiquitination of hexokinase 2 (HK2), and the HectH9-HK2 pathway can regulate the maintenance of PC3-derived PCSCs [17]. Another in vitro study shows that increase of cell density of PCSCs-enriched non-adherent-cultured spheroids derived from PC3 cells can drive a metabolic shift from glycolysis to OXPHOS [18]. Moreover, targeting BRD4 using small-molecule inhibitors or via genetic knockdown in multiple prostate cancer models can block mitochondrial fission and biogenesis, which resulted in PCSC exhaustion and loss of tumorigenicity [19].

Estrogen-related receptor alpha (ERRα, NR3B1, *ESRRA*), which was originally cloned from the fetal kidney and adult heart cDNA libraries and shares high homology with estrogen receptor α (ERα) in its DNA and ligand-binding domain (LBD), is a ligand-independent orphan nuclear receptor [20]. Studies in past decades indicate that ERRα, together working with its coactivators PGC-1s (α or β), performs a central role in the regulation of fatty acid oxidation, energy metabolism/cellular bioenergetics and mitochondrial functions [20, 21]. Our previous studies characterize that ERRα can perform multiple oncogenic functions in prostate cancer, including promotion of hypoxic growth via its direct interaction with HIF-1α and enhancement of HIF-1 signaling [22], transactivation of oncogenic *TMPRSS2:ERG* fusion gene and formation of a transcriptional reciprocal loop with ERG [23] and regulation of intratumoral androgen biosynthesis in castration-resistant prostate cancer (CRPC) via its transcriptional control of multiple steroidogenic enzymes [24].

In the present study, we demonstrate a novel role of ERRα in directing the glucose metabolism in CRPC cells and PCSCs which exhibit higher levels of OXPHOS and ATP production but less rely on lactate-directed glycolysis, via its direct transactivation of *ACO2* (mitochondrial aconitase) in modulating TCA cycle and transrepression on *ZIP1* (*SLC39A1*; Zn transporter 1) in reducing intracellular Zn levels leading to enhancement of mitochondrial aconitase activity.

## Materials and methods

### Cell lines and cell cultures

Four human prostate cancer cell lines (including LNCaP, DU145, PC3 and 22Rv1; ATCC) were used in this study. 293FT (Invitrogen) and PA317 (ATCC) viral packaging cells were used in transfection and lentiviral packaging respectively. (a) Adherent 2D-cell culture conditions were followed as described previously [25, 26]. (b) A previously established agar-based non-adherent 3D-cuture method was followed for isolation and enrichment of prostate cancer stem cells (PCSCs) [27]. In brief, cells were suspended in warm serum free medium-soft agar mixture [1:1 ratio of serum-free medium with supplements (DMEM/F12K with 20 ng/ml EGF, 20 ng/ml FGF-2, 4 μg/ml insulin, 1% KO-serum, 2% B-27, 1% penicillin-streptomycin) and 0.6% soft agar] and seeded onto 0.9% soft agar-precoated 48-well plates at a density of 2 × 10^3^ cells/well. After agar solidification, additional serum free medium (500 μl) was added to each well. After culture for 14–28 days, prostatospheroids formed were imaged and counted under microscope and collected for further analyses. The spheroid formation capacity (stemness) was assayed by counting of spheroids (sizes > 50 μm) formed per 2 × 10^3^ cells seeded.

### Plasmid construction and lentiviral transduction

(a) Expression plasmids. Full-length human ERRα cDNA was PCR-amplified using pSG5-ERRα [25, 28] and subcloned into plenti-V5-topo as plenti (blasticidine)-ERRα for lentiviral transduction or pcDNA3.1 as pcDNA3.1-ERRα for transfection. pcDNA3.1-ERRα-ΔLBD-AF2 (ERRα mutant with deletion of ligand binding and activation function-2 domains) was constructed previously [22]. (b) Luciferase reporter plasmids. Gene promoter fragments of *ACO2* (−2922 to +233 bp) and *ZIP1* (−6817 to −5196 bp) were PCR-amplified from genomic DNA extracted from DU145 cells and cloned into pGL4.11 as pGL4-ACO2 and pGL4-ZIP1 for luciferase reporter assay. pGL4-ΔACO2 and pGL4-ΔZIP1 with deletion of ERRα-binding elements in respective *ACO2* and *ZIP1* promoter sequences were constructed by fusion-PCR using their reporters with wild-type promoter sequences. (c) Gene knockdown shRNA plasmids. Sets of lentiviral plasmids for ERRα knockdown (pLKO.1 (puromycin)-shERRα179 (#1)/180 (#2) were obtained from Dharmacon. shRNA plasmids targeting ACO2 (pLKO.1 (puromycin)-shACO2#1/#2) and ZIP1 (pLKO.1 (neomycin)-shZIP1#1/#2) were constructed according to the RNAi Consortium. (d) Stable clones with ERRα-expression or ERRα/ACO2/ZIP1 knockdown. Prostatic cells were infected with respective infecting viruses together with 8 μg/ml hexadimethrine bromide for 24 h, followed by antibiotic selection for 8 days. (e) SORE6-GFP reporter, which contains six tandem repeats of a composite Sox2/Oct4 response element derived from the human *NANOG* promoter, was used to isolate PCSCs by flow cytometry as described previously [27, 29].

### Gene expression and genomics analyses

Expression profiles of ESRRA, ACO2, and SLC39A1 (ZIP1) in clinical prostate cancer tissues were acquired from datasets available from the cBioPortal for Cancer Genomics (http://www.cbioportal.org/). The ERRα-binding sites present at the 5’-flanking regions of ACO2 and SLC39A1 genes were searched and analyzed using the online MatInspector tool (http://mygga.genomatix.de) [30].

### Molecular biology and immunoblot analyses

(a) Quantitative RT-qPCR analysis. Total cellular RNA was extracted from cultured cells and prostatospheroids using TRIzol reagent (Molecular Research Center), followed by reverse transcription for cDNA synthesis (PrimeScript RT reagent kit with gDNA eraser, TaKaRa). A SYBR Green-based qPCR assay for gene expression analysis was performed using a real-time PCR system (ABI ViiA 7 Real-Time PCR system), with cycle conditions as described previously [31]. Information of primers used is listed in Supplementary Table S1 (b) Chromatin immunoprecipitation (ChIP)-PCR assay. ChIP-PCR assay of *ACO2* and *ZIP1* promoters was performed in DU145 cells, following procedures as described previously [32] using a commercial kit (SimpleChIP Enzymatic Chromatin IP kit, Cell Signaling Technology). Briefly, cross-linked and sonicated DNA samples (300–10^3^ bp fragments) were immunoprecipitated with anti-ERRα antibody or negative control IgG antibody, followed by PCR assay using primers specific for the *ACO2* and *ZIP1* promoters listed in Supplementary Table S1. (c) Luciferase reporter assay. Dual luciferase reporter assay was performed in DU145 or LNCaP cells co-transfected with 0.2 μg reporter plasmids (pGL4 or pGL4-ACO2/-ZIP1 or pGL4-ΔACO2/-ΔZIP10, 0.4 μg expression plasmids (pcDNA3.1 or pcDNA3.1-ERRα/-ERRα-ΔLBD-AF2, pcDNA3-PGC1α) and *Renilla* control reporter pRL-CMV using jetPRIME transfection reagent (Polyplus). Reporter activity was determined following procedures described previously [33]. All assays were repeated in independent triplicates. (d) Immunoblot analysis. Total cellular proteins extracted from cultured prostatic cells and prostatospheroids were separated by the conventional SDS-PAGE electrophoresis, followed by immunoblot analysis using an enhanced chemiluminescence method [31]. Primary antibodies used are listed in the Supplementary Table S1.

### In vitro growth analyses

(a) Cell sorting. CD44^+^ cell population derived from 2D-cultured prostatic cells or 3D-cultured prostatospheroids were analyzed by anti-CD44-based FACS performed on a flow cytometer as described previously (BD FACS Aria Fusion Cell Analyzer) [27]. Sorted cells were collected for molecular analyses and 3D-spheroid formation assay. (b) Spheroid formation assay. Cells derived from 2D-cultures were suspended in serum-free medium and seeded at a density of 2 × 10^3^ cells/well in 0.9% soft agar-coated 48-well plates and cultured for 14–28 days as described above. The spheroid formation capacity (stemness) was assayed by counting of spheroids (sizes > 50 μm) formed per 2 × 10^3^ cells seeded.

### Mitochondrial staining and assay

(a) JC-1 staining of mitochondria. Active mitochondria membrane potential in 2D-cultured prostatic cells, 3D-cultured prostatospheroids, DU145-SORE6-GFP-positive and DU145-SORE6-GFP-negative cells were determined. Briefly, samples were stained with a dual-emission potential-sensitive fluorescent probe JC-1 (5 μM; Invitrogen/ThermoFisher Scientific) and visualized under a confocal scanning microscope following procedures described previously [32]. (b) MitoTracker Orange staining for mitochondrial mass analysis. Above samples were also stained with MitoTracker Orange (150 nM; Invitrogen) in dark at 37 °C for 30 min. After staining, 1 × 10^5^ cells/sample were sorted and analyzed for mitochondrial mass by flow cytometry.

### In vivo tumorigenicity and castration-relapse growth assays

In vivo tumorigenicity of ERRα/shERRα-transduced prostate cancer cells and isolated PCSCs grown in intact SCID/NOD male mice was evaluated by the subcutaneous injection method following procedures described previously [22, 28]. For evaluation of responsiveness toward androgen-deprivation, castration of host mice bearing tumor xenografts formed by prostate cancer cells were performed following procedures described previously and growth of castration-refractory tumors was evaluated [23, 34]. The growth of tumors was evaluated by measuring their sizes using the formula [0.52 × length × width × height] (mm^3^). At the end of experiments, tumors were dissected/harvested for various analyses or histological preparation. All animal experiments were performed following the institute guidelines and with prior approval by the University Animal Experimentation Ethics Committee, CUHK.

### Energy metabolism analysis

The cellular status of glycolysis and mitochondrial respiration, in term of basal extracellular acidification rate (ECAR) and oxygen consumption rate (OCR), of 2D-cultured prostatic cells or 3D-cultured prostatospheroids were determined by using the Seahorse XF96 Extracellular Flux Analyzer according to manufacturer’s suggested procedures with modifications. For glycolysis stress assay, glucose (10 mM), oligomycin (2 μM), and 2-deoxy-D-glucose/2-DG (50 mM) were added sequentially to assay medium containing L-glutamine (2 mM); while for mitochondrial stress assay, oligomycin (1 μM), carbonyl cyanide-p-trifluoromethoxyphenylhydrazone/FCCP (0.5 μM), and rotenone/antimycin A (1 μM) were added to the assay medium containing glucose (25 mM), pyruvate (2 mM) and L-glutamine (2 mM). In brief, cells or prostatospheroids were seeded at 2–3 × 10^4^ cells/well in growth medium in polylysine-coated 96-well plates followed by culturing for 5 h for attachment/sedimentation, briefly washing with XF base medium and culturing in CO_2_-free incubator for 1 h before assay.

### Intracellular ATP analysis

The intracellular ATP levels were determined by a method of detection of ATP-driven luminescence activation of luciferin in metabolic active cells (CellTiter-Glo® Luminescent Cell Viability Assay, Promega) following manufacturer’s instruction. In brief, suspended cells were seeded at a density of 3 × 10^4^ cells/well/100 ml in culture medium in opaque-walled polylysine-coated 96-well plates and incubated for 5 h, followed by addition of reconstituted CellTiter-Glo® Reagent containing reconstituted luciferase and luciferin (100 ml) and gentle shaking on an orbital shaker for 2 min for cell lysis. After incubation at room temperature for 10 min for luminescence signal stabilization, luminescence was recorded using a microplate luminometer (Victor3 V 1420 Multilabel Counter, PerkinElmer). For comparison of ATP levels between prostatospheroid-derived PCSCs and their corresponding 2D-cultured adherent cells, both cells were pre-cultured in culture medium for 5 h, followed by incubation in assay medium as used for the mitochondrial stress assay for another 1 h before ATP measurement of luminescence by procedures as described above.

### Cellular Zn detection and measurement

The intracellular Zn^2+^ was detected using a Zn^2+^-selective fluorophore zinquin ethyl ester (Abcam) following a previous method with modifications [35]. In brief, cells were seeded on polylysine-coated confocal dishes in growth medium supplemented with 10 μM ZnSO_4_ and incubated for 6 h. After brief washing with PBS, cells were further incubated in PBS with 20 μM zinquin ethyl ester for 30 min at room temperature. The fluorescence signals (excitation/emission wavelength: 405/422 nm) were detected and imaged under a confocal scanning system (Olympus FluoView FV1000) and a quartz ×100 oil-immersion objective. For quantitation of cellular Zn^2+^ concentration, cells were seeded at a density of 1.7 × 10^5^ cells/well in polylysine-coated 96-well plates in growth medium supplemented with 10 μM ZnSO_4_ for 5 h. Fluorescence signals were measured using a fluorescence microplate reader (Tecan).

### Statistical analysis

All data are presented as mean ± SD from at least three independent experiments. Results were analyzed using two-tailed Student’s *t*-test for their significant difference. Differences were considered as significant with *P* values < 0.05.

## Results

### Isolated prostate cancer stem cells exhibit increased expression of ERRα

**Fig. 1:**
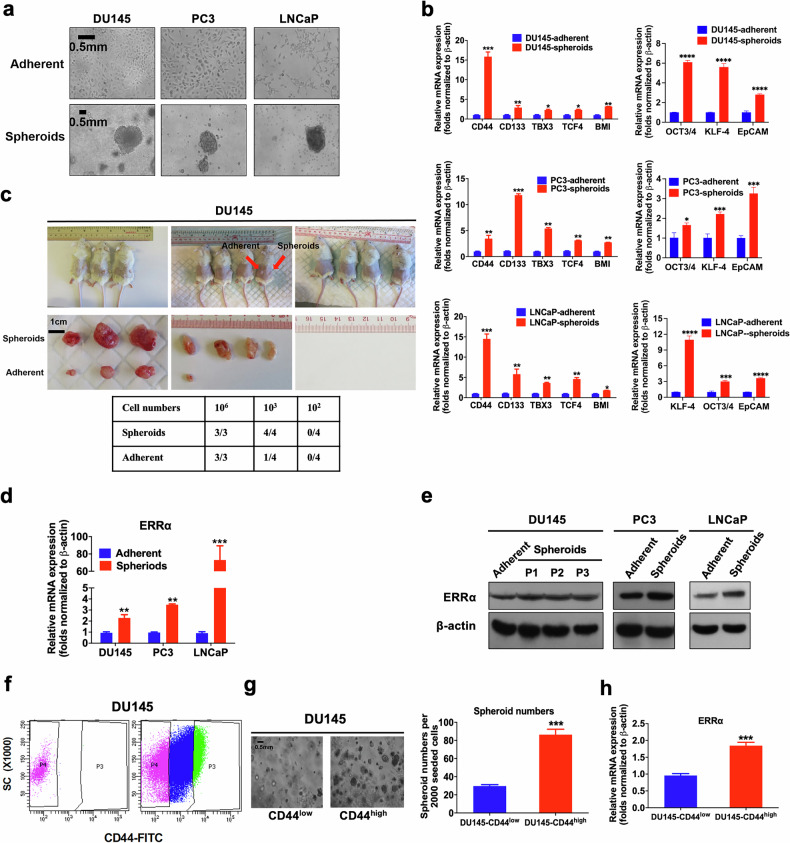
Isolated PCSCs exhibit increased ERRα expression. **a** Representative images of DU145, PC3 and LNCaP cells grown under either adherent 2D- or non-adherent 3D-cultured (spheroids) conditions. Bars: 500 μm. **b** qPCR analyses. 3D-cultured DU145-, PC3- and LNCaP-spheroids exhibited significant higher mRNA levels of multiple CSC-associated markers than their corresponding 2D-cultured adherent cells. **c** In vivo tumorigenicity assay of DU145 cells grown under either adherent 2D- (inoculation site: left flank) or 3D-cultured (right flank) conditions at varying cell numbers injected per site. Results showed that DU145 spheroids could form xenograft tumors in all host mice at 10^3^ cells/site, but DU145 adherent cells could generate smaller-sized tumors in only 1/4 of host mice at this same cell number. **d**, **e** qPCR and immunoblot analyses of ERRα expression. DU145-, PC3- and LNCaP-spheroids expressed significant higher mRNA (**d**) and protein levels (**e**) of ERRα than their corresponding adherent cells. **f** CD44 FACS of DU145 cells. Approximately top 10% CD44^high^ (P3) and top 10% CD44^low^ (P4) DU145 cells were sorted out. **g** 3D-cultured spheroid formation assay. Left: Representative images of spheroids formed by CD44^low^ and CD44^high^ DU145 cells. Right: Quantification analysis of spheroids formed. Results showed that CD44^high^ DU145 cells formed more and larger spheroids than CD44^low^ cells. **h** qPCR analysis. CD44^high^ DU145 cells expressed significant higher mRNA of ERRα than CD44^low^ cells. Results are mean ± SD of three independent experiments. *P < 0.05, **P < 0.01, ***P < 0.001.

To establish the significance of ERRα as therapeutic target in PCSCs, we used multiple methodological approaches to isolate PCSCs from diverse sources of prostate cancer cells. Firstly, leveraging the anchorage-independent growth characteristic of cancer stem cells, we isolated and enriched the putative PCSCs from three prostate cancer cell lines differing in their AR expression status (AR-positive: LNCaP, AR-negative: DU145 and PC3) using an agar-based non-adhesion method [27]. Under this culture condition, putative PCSCs, expanded from single-cell suspensions, grew as non-adherent spherical aggregates (designated as prostatospheroids or spheroids) (Fig. 1a). qRT-PCR analyses showed that prostatospheroids, expressed significantly higher levels of multiple CSC markers (including the commonly expressed CD44 and CD133) as compared to their counterpart cells grown under the conventional 2D-cultured adherent conditions (Fig. 1b). Furthermore, the prostatospheroids were subjected to a gold standard experimental demonstration of their CSC properties, where the candidate CSC population should possess the ability to initiate novel tumor growth in immunocompromised xenograft recipients with high efficiency relative to bulk tumor cell population. In comparisons of the tumorigenic potential, DU145 prostatospheroids and their adherent counterparts were subcutaneously (s.c.) injected into immunodeficient mice at varying cell numbers. The prostatospheroids consistently initiated tumor growth with 100% efficiency, in stark contrast to the lower tumorigenicity observed in adherent cells, confirming the tumor-initiating capability of the prostatospheroids (Fig. 1c). Consistently, both qRT-PCR and immunoblotting assays revealed that prostatospheroids displayed a significantly higher expression of ERRα than their corresponding adherent cells (Fig. 1d, e). Of note, even after several passages in non-adherent conditions, DU145-derived spheroids continued to express high levels of ERRα, highlighting the stability of this phenotype (Fig. 1e). Lastly, we isolated putative PCSCs (CD44^high^ subpopulation) by anti-CD44-based FACS in DU145 cells. The results revealed that CD44^high^ subpopulation exhibited an enhanced sphere formation capacity and a significant increased mRNA level of ERRα as compared to CD44^low^ subpopulation (Fig. 1f–h). Together, our results showed that PCSCs, isolated from different sources of prostate cancer cells, expressed higher levels of ERRα, suggesting that ERRα could play a positive role in the growth regulation of PCSCs.

### ERRα promotes cancer stem cell phenotypes in prostate cancer

**Fig. 2:**
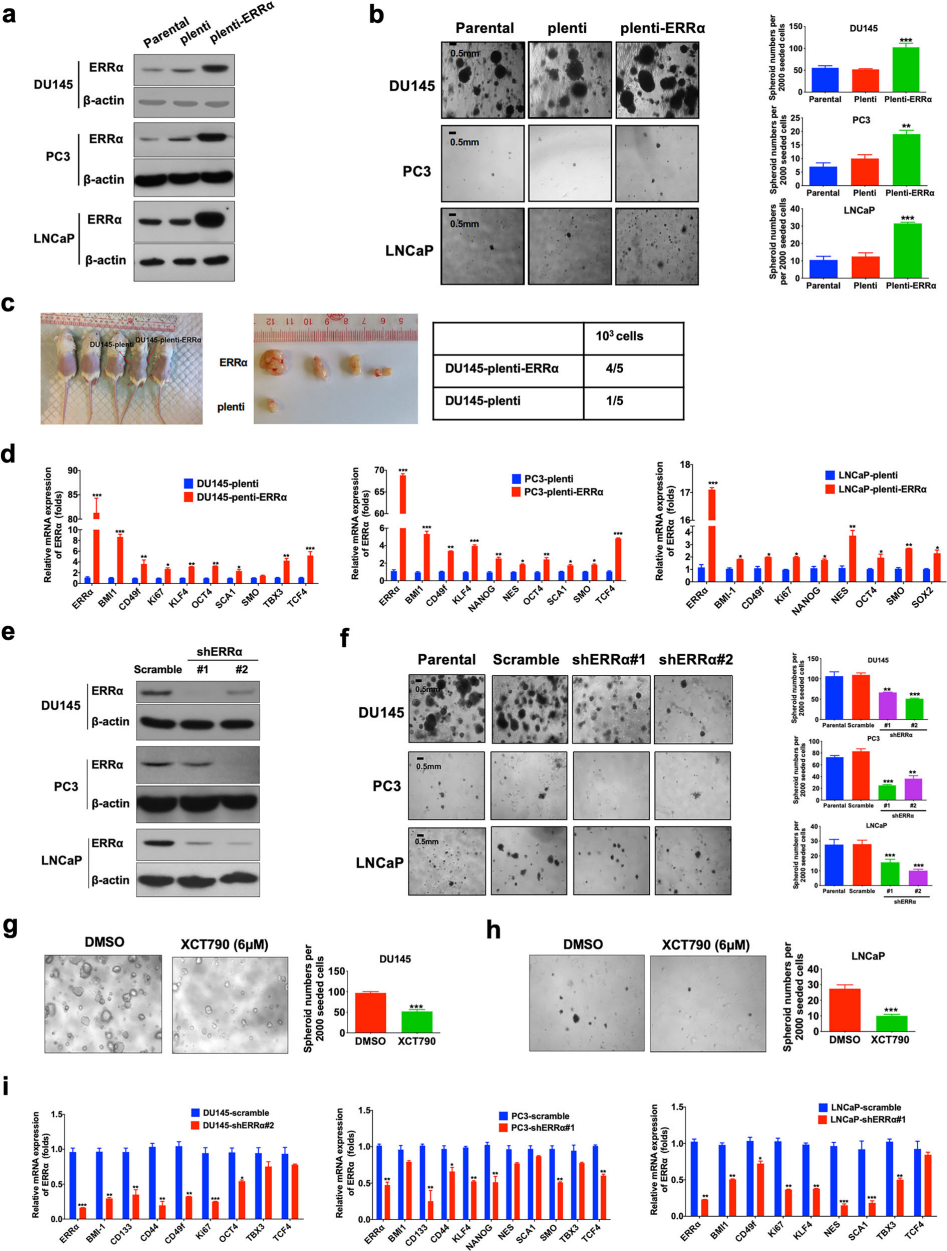
ERRα functions to regulate cancer stem cell (CSC) phenotypes of prostate cancer. **a** Immunoblot validation of ERRα protein expression in stable ERRα-infectants generated from DU145, PC3, and LNCaP cells. **b** 3D-cultured spheroid formation assay. Left: Representative images of spheroids formed by DU145-ERRα, PC3-ERRα, and LNCaP-ERRα infectants and their corresponding controls (parental cells and empty vector infectants). Right: Quantification analyses of spheroids formed. Results showed that DU145-ERRα, PC3-ERRα, and LNCaP-ERRα infectants formed more and larger spheroids than their corresponding controls. **c** In vivo tumorigenicity assay of low-cell-number inoculations (1 × 10^3^ cells/site) of DU145-ERRα versus empty vector infectants (DU145-plenti). Results showed that DU145-ERRα cells could form xenograft tumors in almost all (4/5) host mice, but DU145-plenti cells could generate tumors only in 1/5 of host mice. **d** qPCR analyses. Results showed that DU145-ERRα, PC3-ERRα, and LNCaP-ERRα infectants expressed significant higher mRNA levels of CSC-associated markers than their corresponding empty vector controls. **e** Immunoblot validation of the constructed ERRα-specific shRNA expressing vector on its ERRα-knockdown efficiency in DU145, PC3, and LNCaP cells. **f** Representative confocal microscopic images (Left) and quantification analyses (right) of spheroids formed by the shERRα infectants and control scramble-infectants in DU145, PC3, and LNCaP cells. Results showed that DU145-shERRα, PC3-shERRα and LNCaP-shERRα infectants formed less and smaller spheroids than their corresponding scramble infectants. **g**, **h** Representative images (left) and quantification analyses (right) of spheroids formed by DU145 and LNCaP cells upon treatment with ERRα-selective inverse agonist XCT790 or vehicle. Results showed that XCT790 (6 μM) could significantly inhibit the spheroid formation capacity of prostate cancer cells. **i** qPCR analyses. Results showed that ERRα knockdown could damage the mRNA expressions of multiple CSC-associated markers in DU145 (left), PC3 (middle), and LNCaP (right) cells. Results are presented as mean ± SD of three independent experiments. *P < 0.05, **P < 0.01, ***P < 0.001 versus corresponding empty vector plenti, scramble controls, or vehicle.

To assess the functional significance of ERRα in the maintenance of PCSCs, we next determined the impact of stable ERRα overexpression on the in vitro self-renewal capacity of prostate cancer cells by non-adherent sphere formation assay. The results demonstrated that ectopic expression of ERRα significantly enhanced the sphere formation capacity in DU145, PC3, and LNCaP cells (Fig. 2a, b). Also, we examined the tumorigenicity of ERRα-overexpressed DU145 cells at low cell numbers (10^3^ per site), which showed that ERRα-overexpressed infectant was endowed with a significant higher probability of tumor formation compared with its empty vector counterparts (Fig. 2c). Moreover, ERRα overexpression induced a remarkable upregulation of multiple CSC-associated markers across various prostate cancer cell lines (Fig. 2d). On the contrary, suppression of ERRα, achieved either through shRNA-mediated knockdown or the application of the ERRα inverse agonist XCT790, significantly inhibited the sphere formation capacity (Fig. 2e–h) and decreased the CSC-associated markers expression (Fig. 2i). Collectively, these results underline the critical function of ERRα in sustaining the stem-like properties of prostate cancer cells.

### ERRα facilitates an energy metabolism shift towards OXPHOS in PCSCs

**Fig. 3:**
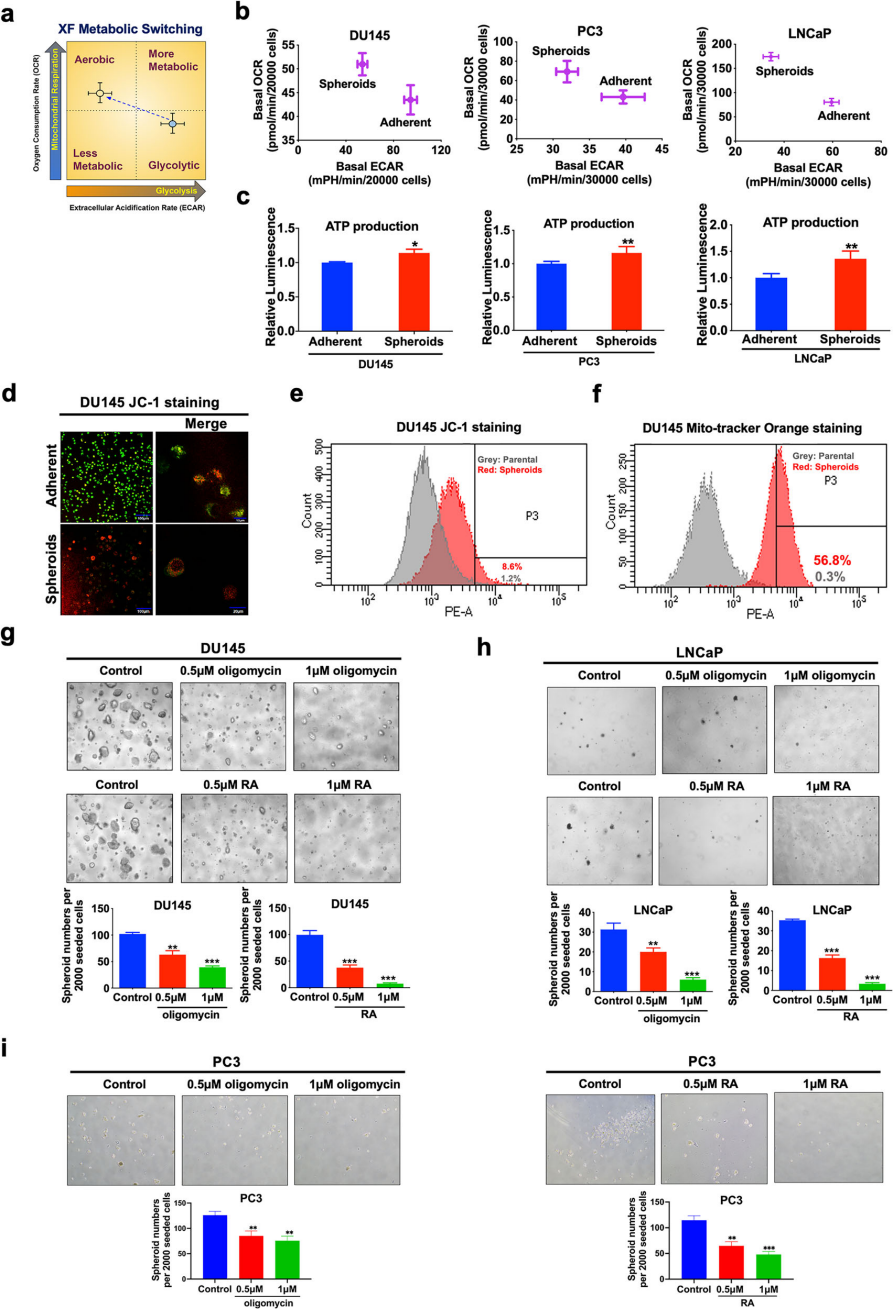
Isolated PCSCs show metabolic switch from glycolysis to OXPHOS. **a** Schematic diagram illustrating the relative metabolic state of cells under two conditions (mean oxygen consumption rate/OCR on the y-axis vs. extracellular acidification rate/ECAR on the x-axis) measured by Seahorse Extracellular Flux/energy analyzer (XFe96, Seahorse Bioscience). **b** Metabolic Switching assessment. Results showed that 3D-cultured DU145-, PC3-, and LNCaP-spheroids exhibited higher OCR but lower ECAR than their corresponding 2D-cultured adherent cells. **c** Measurement of ATP production. Results showed that DU145-, PC3-, and LNCaP-spheroids exhibited higher ATP production as compared to their corresponding adherent 2D-cultured cells. **d**, **e** Mitochondrial membrane potential (MMP) determined by JC-1 fluorescence staining (the higher the ratio of red to green, the higher the MMP). Representative images of JC-1 staining examined by confocal microscopy (**d**) and quantitative analyses of red intensity measured by FACS (**e**) in 2D- and 3D-cultured DU145 cells. Results showed that DU145-spheroids displayed higher MMP as compared to its 2D-cultured adherent cells. **f** Analysis of MMP-dependent mitochondrial mass in DU145 grown under adherent 2D- and 3D-cultured conditions using mitochondrial probe MitoTracker Orange by FACS. Results showed that DU145-spheroids exhibited an increase in MM as compared to its 2D-cultured adherent cells. **g**–**i** 3D-cultured spheroid formation assay. Representative images (upper) and quantification analyses (lower) of spheroids formed by DU145 (**g**), LNCaP (**h**), and PC3 (**i**) cells upon treatment with oligomycin, or rotenone plus antimycin (RA). Results showed that treatment with oligomycin or RA could significantly inhibit the spheroid formation capacity of prostate cancer cells. *P < 0.05, **P < 0.01, ***P < 0.001 versus adherent 2D-cultured cells or vehicle.

To elucidate the roles of ERRα in bioenergetics metabolism regulation in PCSCs, we employed the Seahorse Extracellular Flux analyzer (XFe96) to assess the mitochondrial respiration (oxygen consumption rate/OCR) and glycolysis (extracellular acidification rate/ECAR) status in PCSC-enriched prostatospheroids (Fig. 3a). The prostatospheroids derived from DU145, PC3, and LNCaP cells exhibited elevated OCR and ATP production, along with reduced ECAR compared to their respective adherent parental cells (Fig. 3b, c), indicating a reliance on OXPHOS over glycolysis. Moreover, DU145 prostatospheroids demonstrated a higher mitochondria membrane potential (MMP) (Fig. 3d, e) and an increase in mitochondrial mass (MM) (Fig. 3f) relative to its adherent parental cells. Similar increases in MMP and MM were also detected in PC3- and 22Rv1-derived prostatospheroids as compared to their corresponding adherent 2D-cultured cells (Supplementary Fig. S1a–S1d). Consistently, FACS-sorted SORE6-positive DU145 and LNCaP cells also presented higher MMP and MM than their corresponding SORE6-negative cells (Supplementary Fig. S1e, S1f). Together, these results strongly suggest that mitochondrion in PCSCs exhibited higher activity and mass. Interestingly, pharmacological inhibition of the OCR by mitochondrial ATP synthase inhibitor oligomycin or rotenone plus antimycin (RA) significantly impeded the sphere formation capacity of DU145, LNCaP, and PC3 cells in a dose-dependent manner (Fig. 3g–i). These results suggest that PCSCs prefer OXPHOS as their primary source of energy, and disturbance of the OXPHOS in PCSCs may impair their stem-like properties.

**Fig. 4:**
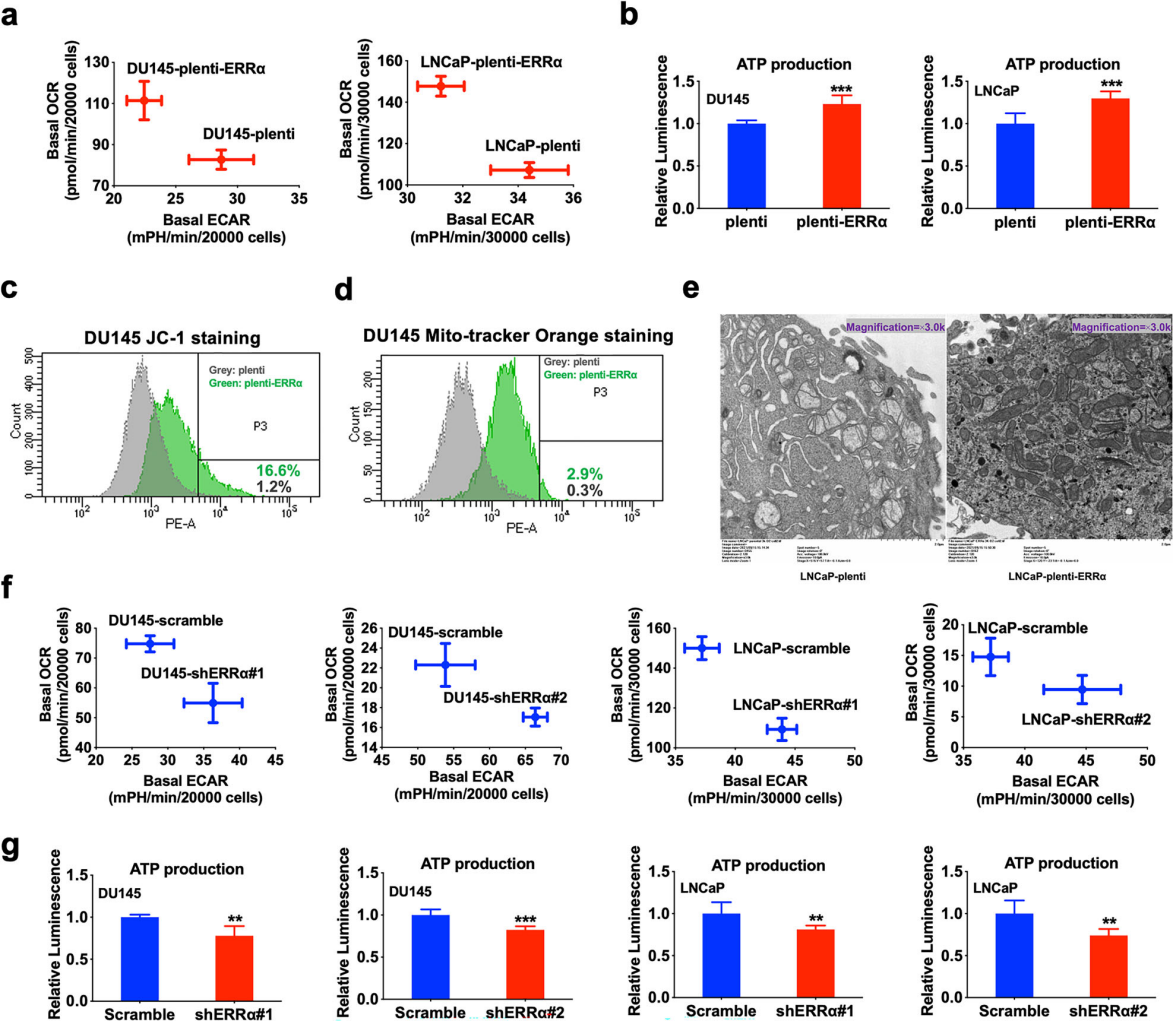
ERRα promotes OXPHOS and ATP production in prostate cancer. **a** Metabolic Switching assessment by Seahorse Extracellular Flux/energy analyzer. Results showed that DU145-plenti-ERRα and LNCaP-plenti-ERRα infectants exhibited higher OCR but lower ECAR than their corresponding empty vector controls (DU145-plenti or LNCaP-plenti). **b** Measurement of ATP production. Results showed that DU145-plenti-ERRα and LNCaP-plenti-ERRα infectants exhibited higher ATP production as compared to their corresponding empty vector controls. **c** MMP determined by JC-1 fluorescence staining. Quantitative analyses of red intensity of JC-1 staining by FACS revealed that DU145-plenti-ERRα cells displayed higher MMP as compared to DU145-plenti cells. **d** Analysis of MMP-dependent mitochondrial mass using mitochondrial probe MitoTracker Orange by FACS. Results showed that DU145-plenti-ERRα exhibited increased mitochondrial mass as compared to DU145-plenti cells. **e** Transmission electron microscopy (TEM) of LNCaP-plenti-ERRα and LNCaP-plenti cells. Results showed that LNCaP-plenti-ERRα cells contained an increased number of mitochondria as compared to LNCaP-plenti cells. **f** Metabolic Switching assessment. Results showed that DU145-shERRα and LNCaP-shERRα infectants exhibited lower OCR but higher ECAR than their corresponding scramble controls. **g** Measurement of ATP production. Results showed that DU145-shERRα and LNCaP-shERRα infectants exhibited Lower ATP production as compared to their corresponding scramble controls. **P < 0.01, ***P < 0.001 versus corresponding empty vector plenti or scramble controls.

We subsequently sought to evaluate the energy metabolism status of prostate cancer cells with either overexpression or knockdown of ERRα. Results showed that ERRα overexpression markedly enhanced the OCR (OXPHOS) and ATP production (Fig. 4a, b; Supplementary Fig. S2a, S2c), increased MMP (Fig. 4c), and augmented mitochondrial mass and quantity (Fig. 4d, e) within prostate cancer cells. Conversely, ERRα knockdown inhibited OCR and ATP production (Fig. 4f, g; Supplementary Fig. S2b, S2d) across various prostate cancer cells. Taking together, these results imply that ERRα-mediated growth regulation of PCSCs could involve its facilitation of an energy metabolism shift towards TCA cycle coupled with OXPHOS.

### ERRα regulates ACO2 and ZIP1, two key citrate metabolism markers in prostate cancer cells

**Fig. 5:**
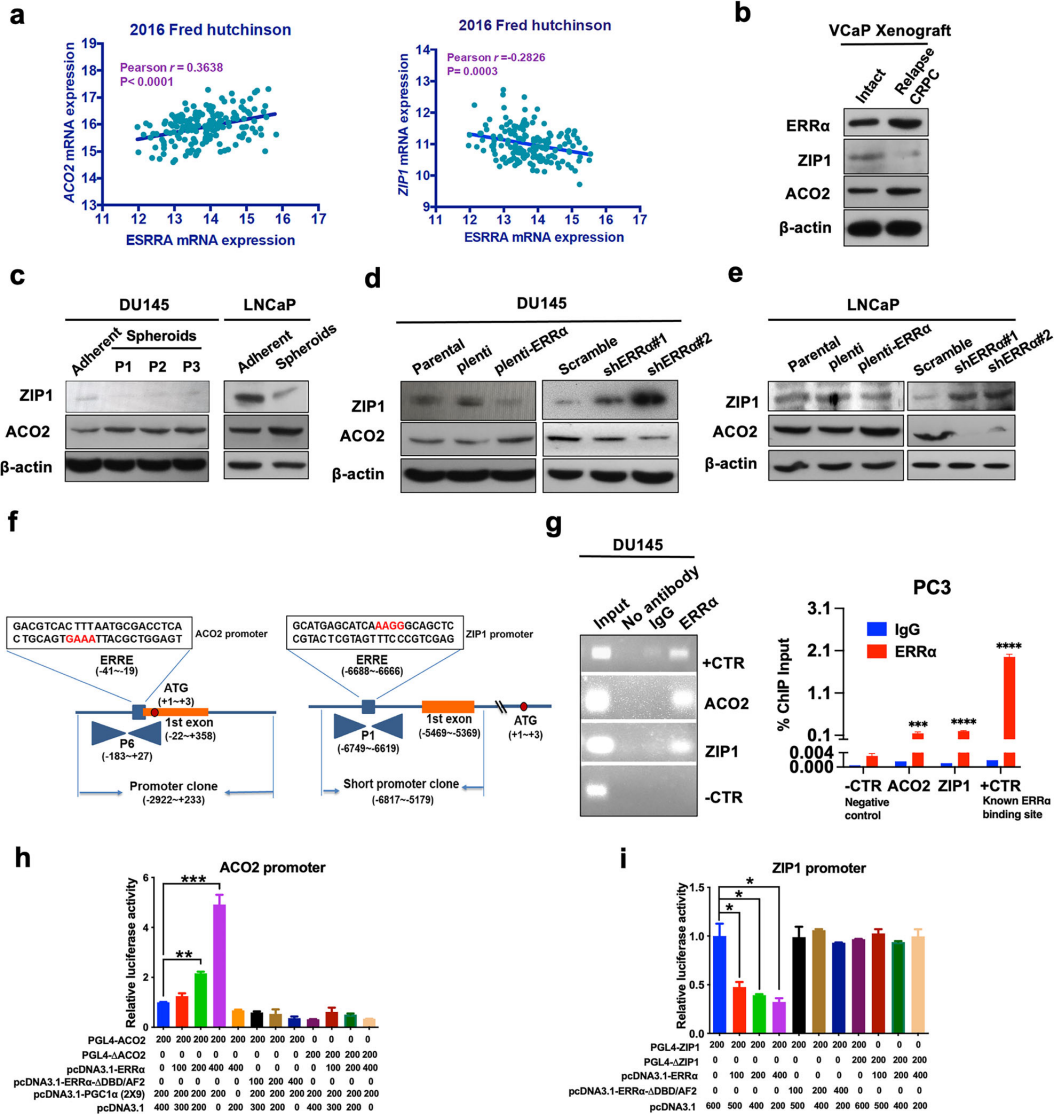
ERRα exhibits distinct reverse expression correlation with two citrate metabolism regulatory markers ACO2 and ZIP1 in prostate cancer. **a** Correlation and linear regression analysis between ERRα (ESRRA) and ACO2 (Left)/ZIP1 (Right) mRNA expressions in clinical metastatic prostate cancer using cancer genomic datasets available from cBioPortal. Results showed that ESRRA and ACO2 manifested a positive, whereas ESRRA and ZIP1 showed a negative expression correlation pattern in metastatic prostate cancer. **b**–**e** Immunoblot analyses of ZIP1 and ACO2 protein expression. **b** The castration-relapsed VCaP-CRPC xenograft tumors expressed lower levels of ZIP1 and higher levels of ACO2 compared with intact VCaP xenograft tumors. **c** DU145-spheroids and their serial passages, as well as LNCaP-spheroids expressed significantly lower levels of ZIP1 and higher levels of ACO2 than their corresponding adherent cells. **d** DU145-ERRα and (**e**) LNCaP-ERRα infectants expressed significant decreased levels of ZIP1 and increased levels of ACO2, whereas DU145-shERRα and LNCaP-shERRα infectants exhibited markable increased levels of ZIP1 and decreased levels of ACO2 (**e**) compared with their corresponding control cells. **f**, **g** ChIP-PCR assay of endogenous ERRα binding performed in DU145 and PC3 cells. **f** Schematic diagram shows the predicted ERRα-binding sites located in the promoter regions of *ACO2* and *ZIP1* gene. **g** Robust binding of ERRα to promoter regions of *ACO2* and *ZIP1* gene were detected. Non-immunoprecipitated DNA was used as input. A known ERRα binding site in ERRα gene promoter was designed as positive control [59], and a region without predicted ERRα response element was designed as negative control. **h**, **i** Luciferase reporter assay of ACO2-Luc and ZIP1-Luc reporters performed in DU145 cells. The ACO2-Luc reporters could be dose-dependently transactivated (**h**), whereas ZIP1-Luc reporters could be dose-dependently transrepressed (**i**) by wild-type ERRα, but not its truncated mutant (ΔDBD/AF), and further potentiated by co-transfection with co-regulator PGC-1α (2 × 9); Deletion of ERRα binding motifs in the ACO2-Luc (PGL4-ΔACO2) or ZIP1-Luc (PGL4-ΔZIP1) significantly abolished ERRα-induced transactivation or transrepression as compared to the wild-type ACO2- or ZIP1-Luc reporters, respectively. Fold changes were normalized to pCDNA3.1. Results are presented as mean ± SD of three independent experiments. *P < 0.05, **P < 0.005, ***P < 0.001 versus respective control.

Among the key TCA outputs, citrate serves as the key intermediate, and normal prostate epithelia produce citrate through a physiologic truncation of the TCA cycle at the level of m-aconitase (ACO2), of which activity is inhibited by the unique high level of intracellular zinc in normal prostate gland. In contrast, during oncogenic transformation, normal prostatic epithelia undergo metabolic reprogramming from citrate-accumulating to citrate-oxidizing cancer cells with lower zinc levels and higher ACO2 activity, fueling tumor growth and progression. Since intracellular zinc levels are mainly controlled by zinc transporter ZIP1, we then examined the expression profiles of ERRα, ACO2 and ZIP1 in clinical prostate cancer samples using an available prostate cancer dataset. Results showed that ERRα (ESRRA) and ACO2 manifested a positive expression correlation pattern, whereas ERRα and ZIP1 showed a negative expression correlation pattern in metastatic prostate cancer (Fig. 5a). Our immunoblot results showed that castration-relapse VCaP-CRPC xenografts as well as prostatospheroids of DU145 and LNCaP showed higher ACO2 and lower ZIP1 protein levels as compared to intact VCaP xenografts, DU145 and LNCaP parental adherent cells, respectively (Fig. 5b, c; Supplementary Fig. S2e). Moreover, ERRα overexpression enhanced ACO2 protein expression but weakened ZIP1 expression, whereas ERRα knockdown could reverse the expression pattern of ACO2 and ZIP1 (Fig. 5d, e; Supplementary Fig. S2e).

Importantly, potential ERRα binding sites (ERREs) in the promoter regions of both ACO2 (−41 to −19 bp) and ZIP1 (−6688 to −6666 bp) were predicted via sequence analysis, suggesting that they could be direct transcriptional targets of ERRα (Fig. 5f). To further address this, we performed ChIP experiments on the human *ACO2* and *ZIP1* gene promoter in DU145 and PC3 cells. ChIP-PCR assays showed that ERRα could directly bind to the ERRE-containing sites within *ACO2* and *ZIP1* promoters (Fig. 5g). Next, luciferase reporter gene assay was carried out in DU145 cells, and data revealed that only wild-type ERRα, but not its truncated mutant (ΔDBD/AF), could dose-dependently transactivate the activity of ACO2-Luc promoter, with further potentiation by the ERRα co-activator PGC1α (2 × 9), whereas it could dose-dependently transrepress the activity of ZIP1-Luc promoter (Fig. 5h, i). Lastly, to validate the functional ERREs, luciferase reporter assay was performed with wild-type (PGL4-ACO2 or PGL4-ZIP1) and mutated ACO2-Luc (PGL4-ΔACO2) or ZIP1-Luc (PGL4-ΔZIP1) reporters. Results showed that Luc reporter constructs containing mutation in either of the above ChIP-validated ERREs could significantly impair the ERRα-induced luciferase activity as compared to the wild-type ACO2- or ZIP1-Luc reporter (Fig. 5h, i). Collectively, these results indicated that ERRα could directly activate the *ACO2* gene, and repress the *ZIP1* gene, thus resulting in the dysregulation of zinc homeostasis and citrate metabolism in prostate cancer cells.

### ERRα-promoted growth of PCSCs is mediated by ACO2

**Fig. 6:**
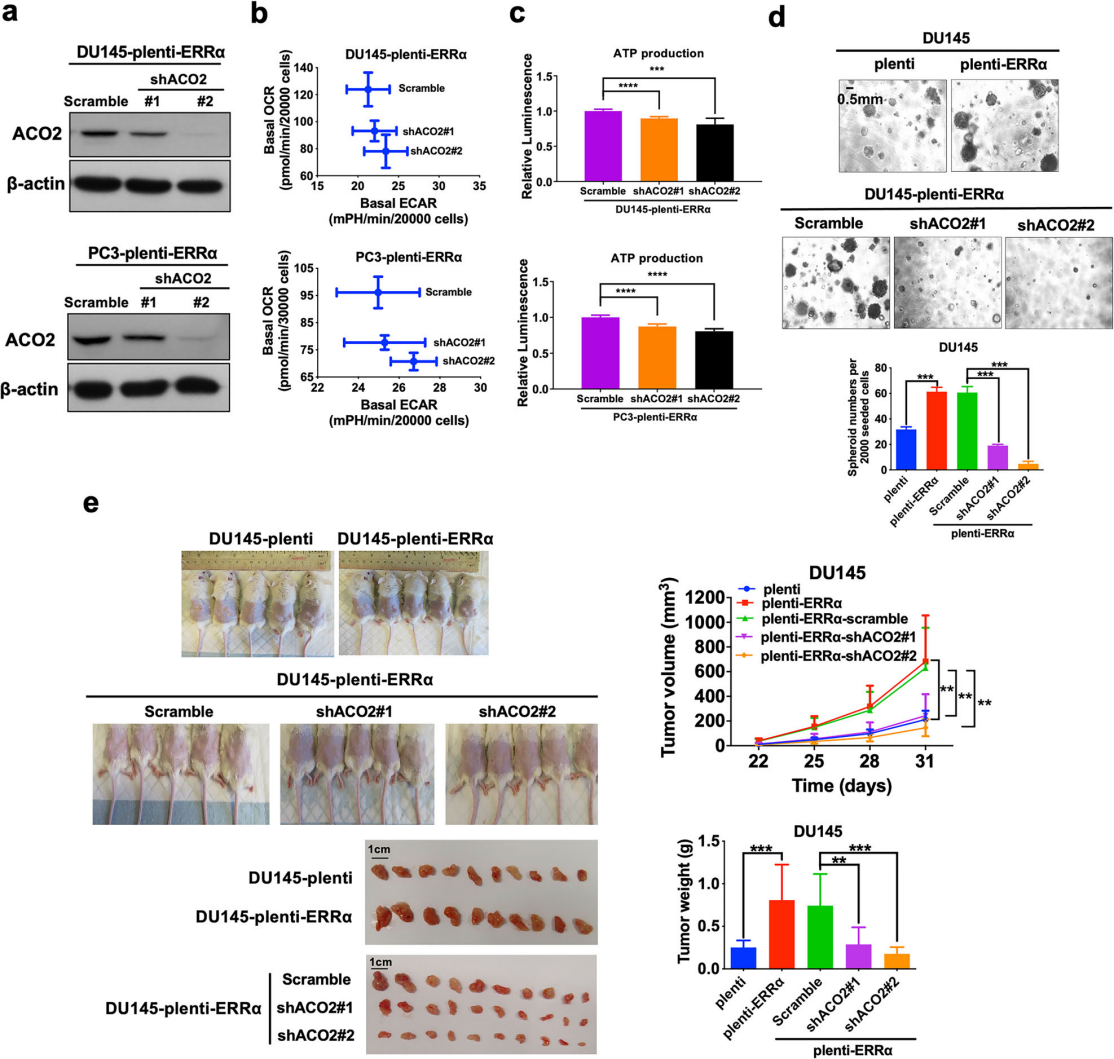
ERRα-promoted growth of PCSCs is reversed by ACO2 knockdown. **a** Immunoblot analysis of AOC2 in DU145-plenti-ERRα and PC3-plenti-ERRα infectants upon ACO2-knockdown. ACO2 protein expression was significantly downregulated in DU145-plenti-ERRα and PC3-plenti-ERRα infectants upon shACO2 constructs transduction. **b**, **c** Metabolic Switching assessment and ATP production measurement. Results showed that ACO2 knockdown could significantly abolish the increased OCR (**b**) and reverse the increased ATP production (**c**) mediated by ectopic expression of ERRα in DU145 and PC3 cells. **d** 3D-cultured spheroid formation assay. Upper and middle: Representative images of spheroids formed. Bottom: Quantification analyses of spheroids formed. Results showed that DU145-plenti-ERRα and DU145-plenti-ERRα-scramble infectants formed more and larger spheroids than empty vector infectants (DU145-plenti), which could be abolished by ACO2 knockdown. **e** In vivo tumorigenicity assay of DU145-plenti, DU145-plenti-ERRα, DU145-plenti-ERRα-scramble, DU145-plenti-ERRα-scramble-shACO2#1 and DU145-plenti-ERRα-shACO2#2 infectants. Top left: Representative images of host mice bearing tumors formed by the injected infectants; Top right: Graph shows the growth curve of xenograft tumors formed by the infectants. Bottom left: Images of dissected tumors formed by the infectants. Bottom right: Measurement of wet weights of tumors formed by the infectants. DU145-plenti-ERRα cells formed larger tumors than DU145-plenti cells. DU145-plenti-ERRα-shACO2#1 and DU145-plenti-ERRα-shACO2#2 infectants generated smaller tumors at slower rate than those of control DU145-plenti-ERRα-scramble. Results are presented as mean ± SD of three (*n* = 3) independent experiments. **P < 0.01, ***P < 0.001 versus corresponding empty vector plenti or scramble controls.

To further elaborate the functional contribution of ERRα-mediated transactivation of ACO2 towards promotion of OXPHOS and CSC growth in prostate cancer cells, we downregulated ACO2 in ERRα overexpressed DU145 and PC3 cells (Fig. 6a) and assessed their metabolic status. The results revealed that shRNA-mediated ACO2 knockdown could revert, at least partially, the metabolic shift from glycolysis to OXPHOS and the increase in ATP production triggered by ERRα overexpression in DU145 and PC3 cells (Fig. 6b, c). As expected, the enhanced in vitro sphere formation capacity and in vivo tumorigenicity mediated by ERRα overexpression were also conspicuously abolished upon ACO2 knockdown in DU145 and PC3 cells (Fig. 6d, e; Supplementary Fig. S3a, b). These results suggest that transactivation of ACO2 was involved in the ERRα-mediated energy metabolism shift towards OXPHOS and CSC maintenance in prostate cancer cells.

### ERRα represses zinc transportation into prostate cancer cells via its negative control on ZIP1 expression

**Fig. 7:**
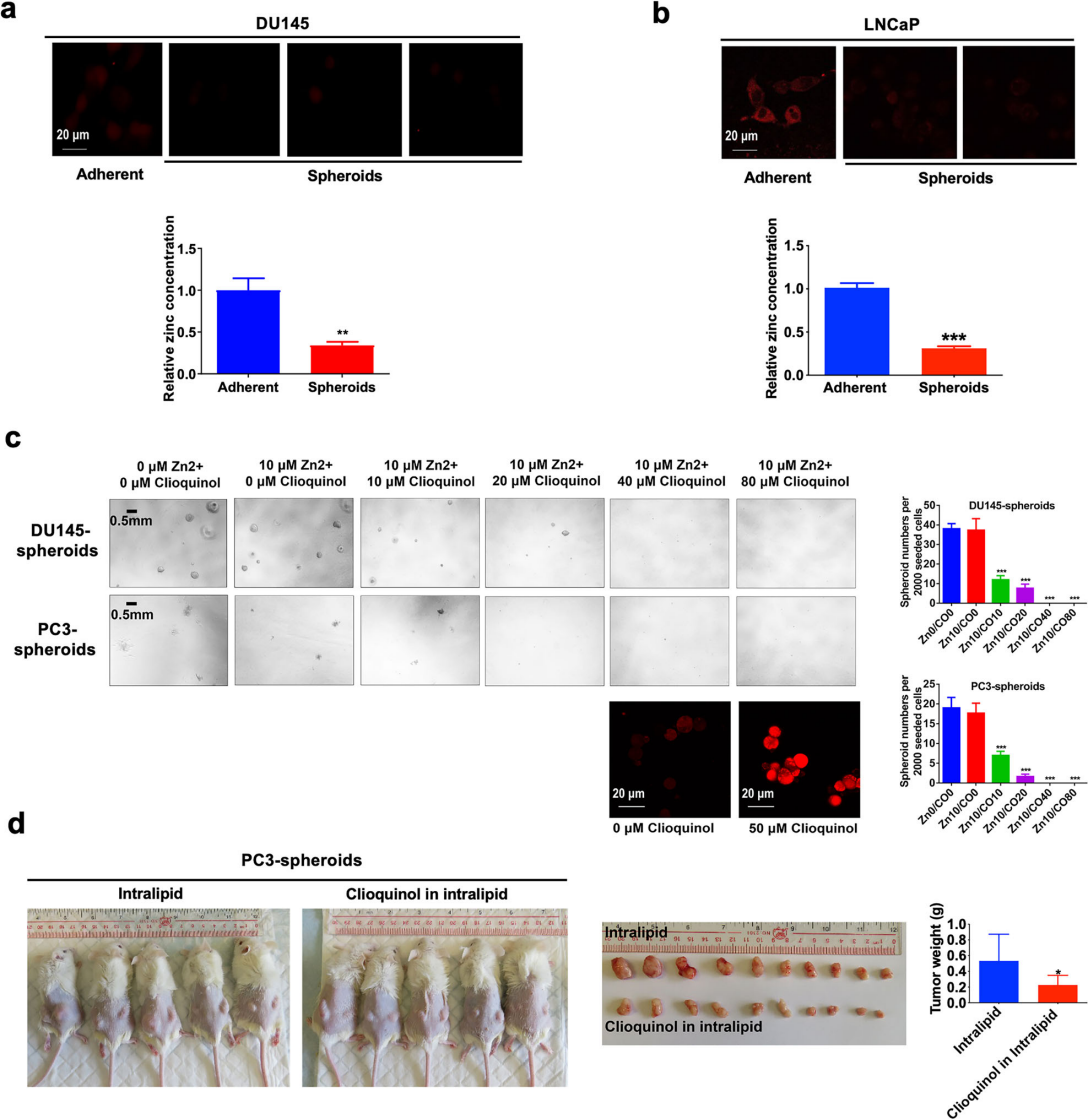
Intracellular Zinc accumulation suppresses the stemness of prostate cancer. **a**, **b** Intracellular zinc levels measured using a zinc-selective fluorescent probe (zinquin ethyl ester). Top: Representative images of zinquin ethyl ester staining examined by confocal microscopy in DU145 (**a**) and LNCaP (**b**) cells grown under either 2D- or 3D-cultured conditions. Bottom: Quantitative analyses of zinquin ethyl ester staining by microplate reader. Results showed that DU145- and LNCaP-spheroids displayed lower intracellular zinc levels as compared to their corresponding 2D-cultured adherent cells. **c** 3D-cultured spheroid formation assay. Left: Representative images of spheroids formed by the DU145 (Top) and PC3 (Bottom) cells upon treatment with gradient concentrations of clioquinol (a zinc ionophore), supplemented with 10 μM zinc sulfate. Right: Quantification analyses of spheroids formed (Top: DU145; Bottom: PC3). Results showed that treatment with clioquinol significantly repressed the spheroid formation capacity of DU145 and PC3 cells. **d** Tumorigenicity of PC3-spheroids upon in vivo treatment with clioquinol or vehicle (intralipid). Following bilateral inoculation with PC3-spheroids, the host mice were randomly assigned to intraperitoneal injections of clioquinol (dissolved in 20% intralipid and 33 mg/kg in 200 μl) or vehicle every other day for two months. Left: Representative images of host mice bearing tumors formed by the PC3-spheroids upon treatment with clioquinol or vehicle. Middle: Images of dissected tumors. Right: Measurement of wet weights of tumors. Results showed that clioquinol could significantly retard the growth of PC3-spheroid-derived tumors as compared to vehicle. *P < 0.05, **P < 0.01, ***P < 0.001 versus adherent 2D-cultured cells or vehicle.

We then investigated the intracellular zinc levels within PCSC-enriched prostatospheroids using zinquin ethyl ester, a zinc-selective fluorescent probe. Our results showed that PCSC-enriched prostatospheroids manifested substantially low or barely detectable intracellular zinc levels, in stark contrast to the adherent parental cells (Fig. 7a, b; Supplementary Fig. S4a). To explore the impact of intracellular zinc on PCSC growth, we employed a zinc selective ionophore (clioquinol) to increase the intracellular zinc accumulation within PCSCs and assessed its effect on their spheroid formation capacity. Results revealed that clioquinol notably repressed the sphere formation capacity of the DU145 and PC3 cells in a dose dependent manner, highlighting the inhibitory effect of increased zinc on PCSC growth (Fig. 7c). Moreover, the in vivo tumorigenicity of PC3- and LNCaP-spheroids was significantly suppressed upon clioquinol treatment (Fig. 7d and Supplementary Fig. S4b). These findings indicate a critical role for intracellular zinc levels in modulating stem cell-like traits of prostate cancer cells.

**Fig. 8:**
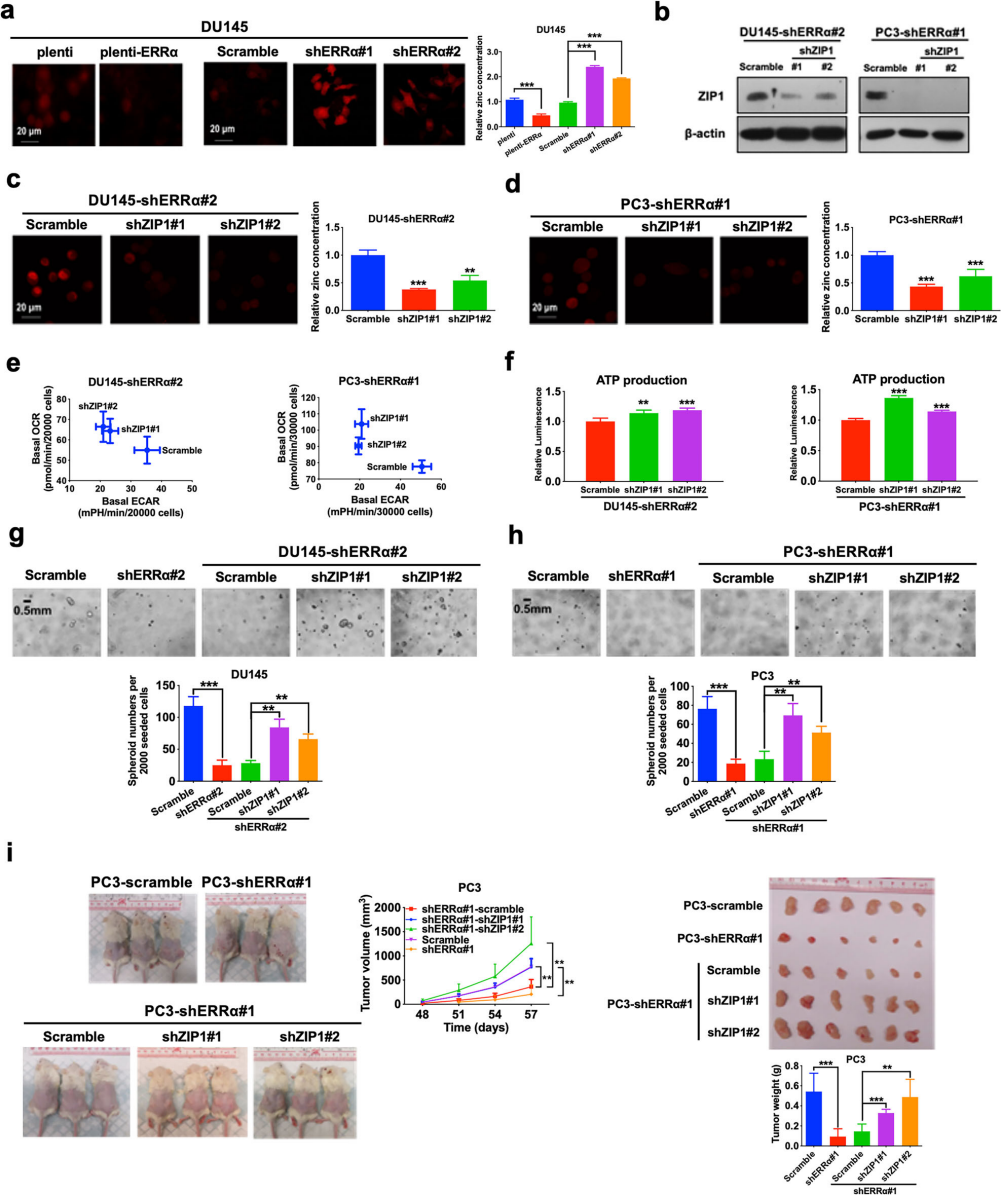
ZIP1 knockdown reverses ERRα-mediated OXPHOS and stemness in prostate cancer. **a** Intracellular zinc levels measured using zinquin ethyl ester. Left and Middle: Representative confocal microscopic images of zinquin ethyl ester staining in DU145-plenti and DU145-plenti-ERRα (Left) as well as DU145-scramble, DU145-shERRα#1 and #2 infectants (Middle). Right: Quantitative analyses of zinquin ethyl ester staining by microplate reader. Results showed that DU145-plenti-ERRα cells exhibited lower intracellular zinc levels as compared to DU145-plenti cells, whereas DU145-shERRα#1 and #2 infectants displayed higher intracellular zinc levels when compared with DU145-scramble infectants. **b** Immunoblot analysis of ZIP1 in DU145-shERRα and PC3-shERRα infectants upon ZIP1 knockdown. ZIP1 protein expression was significantly abolished after transduction with shZIP1 constructs. **c**, **d** Intracellular zinc levels. Representative images and quantitative analyses of zinquin ethyl ester staining in DU145-shERRα#2 (**c**) and PC3-shERRα #1 (**d**) infectants upon ZIP1 knockdown. Results showed that the increased intracellular zinc levels mediated by ERRα knockdown could be significantly abolished by ZIP1 knockdown. **e** Metabolic switching assessment. Results showed that ZIP1 knockdown could significantly reverse the increased ECAR and reduced OCR mediated by ERRα knockdown in DU145 (Left) and PC3 (Right) cells. **f** Measurement of ATP production. ZIP1 knockdown could restore the reduced ATP production mediated by ERRα knockdown in DU145 (Left) and PC3 (Right) cells. **g**, **h** 3D-cultured spheroid formation assay. Top: Representative images of spheroids formed. Bottom: Quantification analyses of spheroids formed. Results showed that DU145-shERRα (**g**) and PC3-shERRα (**h**) infectants formed less and smaller spheroids than their corresponding scramble controls, which could be reversed by ZIP1 knockdown. **i** In vivo tumorigenicity assay. Left: Representative images of host mice bearing tumors formed by PC3-scramble, PC3-shERRα#1, PC3-shERRα#1-scramble, PC3-shERRα#1-shZIP1#1, and PC3-shERRα#1-shZIP1#2 infectants; Middle: Graph shows the growth curve of xenograft tumors formed by the infectants. Right Top: Images of dissected tumors formed by the infectants. Right Bottom: Measurement of wet weights of tumors formed by the infectants. Data showed that PC3-shERRα#1 infectants grew tumors at slower rate and formed tumors with smaller sizes than that of PC3-scramble; while PC3-shERRα#1-shZIP1#1 and PC3-shERRα#1-shZIP1#2 infectants formed xenograft tumors at faster rates and with larger sizes than that of PC3-shERRα#1-scramble infectants. Results are presented as mean ± SD of three independent experiments. **P < 0.01, ***P < 0.001 versus corresponding empty vector plenti or scramble controls.

Prompted by these observations, we hypothesize that the depletion of intracellular zinc levels within PCSCs might be due to ERRα-mediated downregulation of ZIP1. Therefore, we measured the intracellular zinc levels in prostate cancer cells upon either ERRα overexpression or knockdown. Results showed that ERRα overexpression significantly reduced, whereas ERRα knockdown enhanced the intracellular zinc levels in prostate cancer cells (Fig. 8a; Supplementary Fig. S5a, b). To further elucidate the significance of ERRα in ZIP1 regulation and its contribution to the growth of PCSCs, we performed the shRNA-mediated knockdown of ZIP1 in ERRα-knockdowned prostate cancer cells (Fig. 8b), and found that ZIP1 knockdown could abrogate the intracellular zinc accumulation (Fig. 8c, d), and restore the suppressed OXPHOS status (Fig. 8e), ATP production (Fig. 8f), in vitro spheroid formation capacity (Fig. 8g, h) as well as in vivo tumorigenicity (Fig. 8i; Supplementary Fig. S5c) induced by ERRα knockdown in prostate cancer cells. Together, these findings indicated that PCSCs contained low intracellular zinc levels and ERRα could function to repress zinc transportation into prostate cancer cells via its negative control on ZIP1 expression.

## Discussion

It is well established that normal prostate gland contains the highest Zn contents in the body as compared to other tissues or organs. This unique feature of accumulation of high Zn levels leads to high production and secretion of citrate levels by the normal prostate epithelial cells, which is a major function of prostate gland, as high intracellular Zn levels can inhibit the activity of mitochondrial (m-) aconitase, a key enzyme responsible for the oxidation of citrate to isocitrate in TCA cycle [36]. However, the prostatic tissue displays a marked reduction in Zn levels accompanied with a concurrent decrease in citrate in the development and progression of prostate cancer, in contrast to that in normal and benign prostatic hyperplasia (BPH) prostate [37, 38]. As a result, the prostate cancer cells transform to be citrate-oxidizing cells due to m-aconitase activation induced by decreased Zn levels. This unique metabolic shift to higher OXPHOS is believed to be beneficial to primary prostate cancer cells in their cellular bioenergetics for its complete glucose oxidation and more ATP production [39]. Of note, unlike primary prostate adenocarcinoma, metastatic neuroendocrine prostate cancer (NEPC) is considered to be comparatively glycolytic, as it is commonly detectable on fluorodeoxyglucose (FDG) imaging, indicating considerable metabolic plasticity during NE transdifferentiation [6].

In this study, we showed that nuclear receptor ERRα exhibited an increased expression in isolated PCSCs and could act to enhance the growth and stemness of PCSCs. Indeed, upregulation of ERRα together with another ERR family member ERRγ and their cofactors PGC-1α/β, are essential for the metabolic reprogramming to the sudden enhanced OXPHOS in the induced pluripotency in human and mouse stem cells [40]. It is also shown that ERRα-PGC-1α signaling can promote the survival of mesenchymal stem cells via its regulation of a mitochondrial anti-apoptotic regulator Bcl-2 [41]. Another in vitro study suggests that activation of ERRα and TGF-β1 signaling by xenoestrogen bisphenol A can promote the growth and maintenance of human neural stem cells [42]. Moreover, in vitro studies by inhibition of ERRα activity also suggest a positive role of ERRα in the survival and maintenance of breast cancer stem cells via its regulation of mitochondrial biogenesis [43] and malignant features [44]. Together, our present study and others suggest that ERRα can support the growth and maintenance of CSCs, including PCSCs.

In this study, we also demonstrated that treatments with a Zn ionophore Clioquinol could significantly suppress both the in vitro spheroid formation capacity (stemness) and the in vivo tumorigenicity of PCSCs. Our results imply that increase of Zn uptake or cellular accumulation could be a potential therapeutic strategy for growth suppression of PCSCs. Indeed, a previous study also shows that in vivo treatment with clioquinol can suppress the tumor growth of ZIP1-knockdowned PC3 cells [45]. Since prostate cancer is a Zn-deficient malignancy, therapeutic approach of enhanced Zn uptake or cellular accumulation could be of potential therapeutic value or an adjuvant therapy for advanced prostate cancer management.

Among the Zn transporters, ZIP1 is characterized as the major Zn uptake membrane transporter mainly expressed in prostate, which acts to play a key role in regulating the uptake and intracellular Zn accumulation in prostatic epithelial cells and prostate cancer cells [46–48]. Downregulation of ZIP1, which can prevent the uptake and accumulation of Zn, is the major cause for the decreased Zn levels in prostate cancer [46]. However, the mechanism involved in ZIP1 downregulation in prostate cancer is still poorly undefined. In the present study, we characterize that nuclear receptor ERRα, which displays an up-regulation in prostate cancer and a further increase in PCSCs, can directly target and transrepress ZIP1 and through this ERR-mediated down-regulation of ZIP1, ERRα significantly prevents the Zn uptake and accumulation in the isolated PCSCs independent of their AR expression status. Previous studies have identified a few negative regulators of ZIP1 in prostate cancer cells. An early epigenetic analysis shows that it is the hypermethylation of promoter region of transcription factor activating enhancer-binding protein-2α (AP-2α), a regulator of ZIP1, but not the methylation of ZIP1 promoter, is the mechanism responsible for the downregulation of ZIP1 in DU145 and LNCaP prostate cancer cells [49]. A study shows that the zinc-finger transcription factor RREB-1 (RAS-responsive element binding-binding protein 1) can act as a negative regulator of ZIP1 via its binding to the ZIP1 promoter region and mediate downregulation of ZIP1 in PC3 cells [50, 51]. Another study shows that microRNA miR-183-96-182 cluster can downregulate ZIP1 expression in prostate cancer [52].

In this study, we demonstrated that ERRα could play significant roles in the enhancement of tumorigenicity and stemness (in vitro anchorage-independent growth and enhanced expressions of CSCs-associated markers) of prostate cancer cells, regardless of their AR expression status, via its direct control of metabolic shift to be more OXPHOS-dependent and ATP-productive. The metabolic shift to be more OXPHOS dependent with an advantage of enhanced tumorigenic growth and stemness in advanced growth of prostate cancer is mediated by the upstream transcriptional regulation by ERRα that linking the upregulation of ACO2 in TCA cycle and the downregulation of ZIP1 leading to the loss of intracellular Zn. In addition to ACO2 in TCA cycle as characterized in this study, the metabolic shift to be more OXPHOS as induced by ERRα in prostate cancer cells and PCSCs can also be due to its direct regulation of wide set of mitochondrial targets involved in OXPHOS/electron transport [21, 53]. Studies show that CSCs isolated from different tumors display different metabolic and bioenergetic preference. Some studies show that CSCs isolated from lung cancer [54], glioblastoma [12], leukemia [55], and pancreatic ductal adenocarcinoma [56] rely more on OXPHOS for their bioenergy demands. On the other hand, some reports also show that CSCs from lung cancer [15], osteosarcoma [57], breast cancer [14], and ovarian cancer [58] prefer glycolysis as their primary source of energy. Such a discrepancy on the metabolic and bioenergetic profiles of CSCs as reported by various studies, even CSCs derived from the same cancer types, could be attributed to the primary sources (clinical tumor tissues, cell lines-derived xenografts or cancer cell lines) for their isolation, differentiation status of CSCs during quiescence or proliferation, and also their capacity in metabolic shift during lineage plasticity.

## Conclusions

**Fig. 9:**
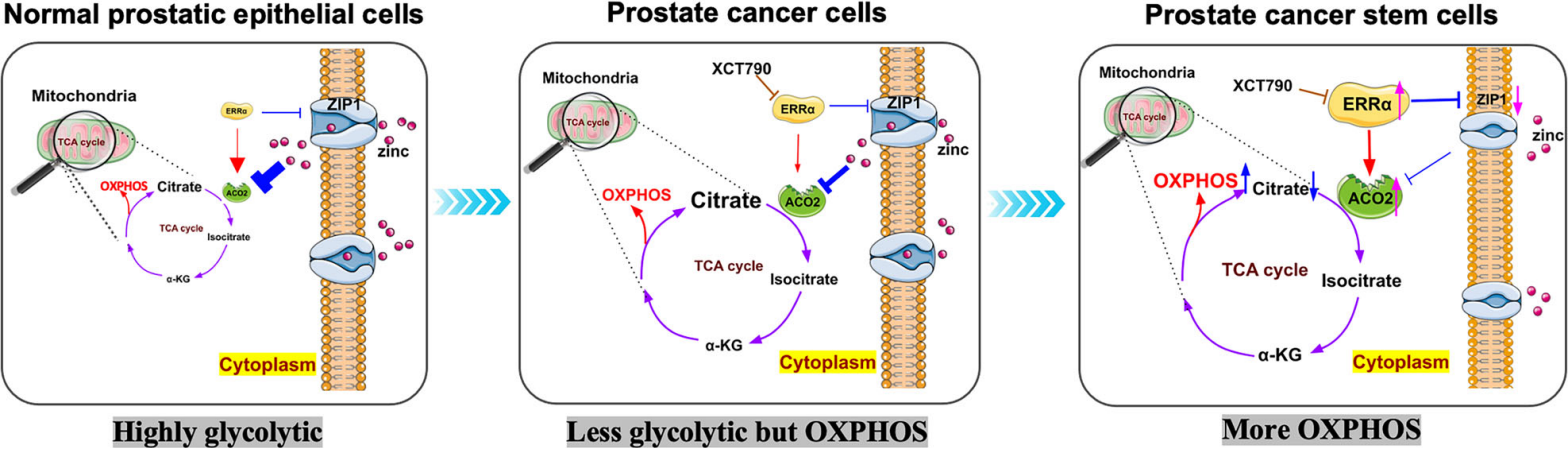
ERRα promotes TCA-OXPHOS in prostate cancer cells and prostate cancer stem cells via its conjoined transcriptional modulation of ACO2 and ZIP1. Schematic diagrams depict the specific roles of ERRα in the control of energy metabolism status in normal prostatic epithelial cells (more glycolytic due to incomplete TCA cycle as being repressed by high intracellular zinc levels), prostate cancer cells and prostate cancer stem cells (less glycolytic but more OXPHOS due to increased and complete TCA as caused by increased ERRα expression resulted in decreased intracellular zinc levels), via its conjoined transrepression of zinc transporter ZIP1 in reducing intracellular zinc uptake and transactivation of aconitase ACO2 in completion of TCA cycle.

In summary, we show here that nuclear receptor ERRα plays a key role in the growth of PCSCs via its control of stemness and cellular energetic metabolism toward more OXPHOS-dependent by a direct and combined transcriptional regulation of ZIP1 transrepression in suppression of Zn uptake and ACO2 transactivation in completion of TCA cycle (Fig. 9). ERRα represents a promising metabolic vulnerability in prostate cancer and PCSCs, and targeting mitochondrial metabolism in PCSCs through inhibition of ERRα provides a novel paradigm for developing more effective therapeutic strategies for advanced prostate cancer.

## Supplementary information

Supplementary Table S1

Supplementary Figures S1-S5

Unchopped Whole Blots

## Data Availability

All data responsible for evaluating the conclusions in this study are presented in the paper and/or the Supplementary Materials. The datasets used and analysed in the study are available from the corresponding author upon reasonable request.
