# Supplementary material for: Estrogen-related receptor alpha (ERRα) controls the stemness and cellular energetics of prostate cancer cells via its direct regulation of citrate metabolism and zinc transportation
Source: Cell Death Dis. 2025 Mar 5;16(1):154. doi: 10.1038/s41419-025-07460-z (PMC11882781; doi:10.1038/s41419-025-07460-z)
Supplement: Supplementary file 2 — Supplementary Figures S1-S5 [file 41419_2025_7460_MOESM2_ESM.pdf]

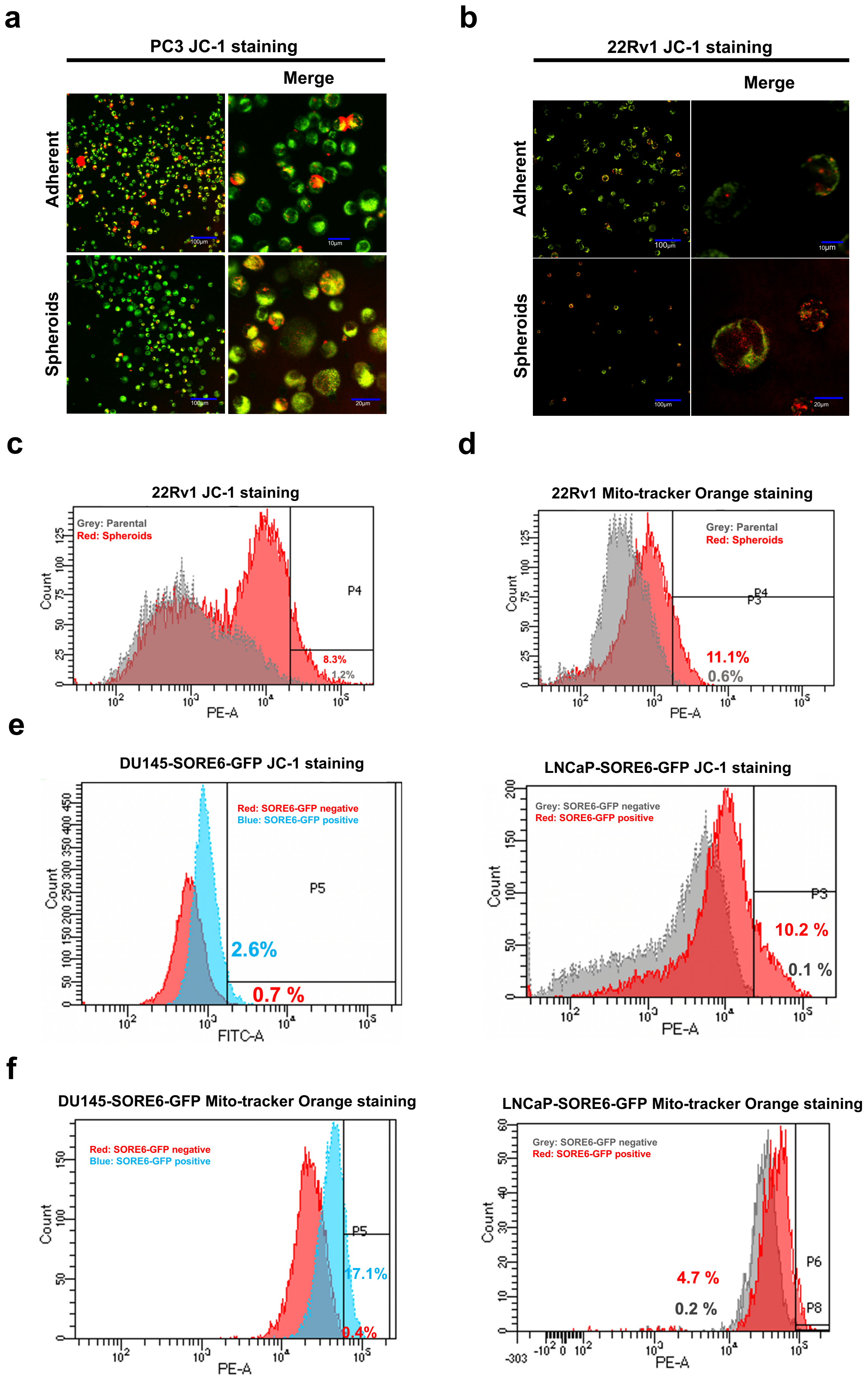

Fig. S1 a-d MMP and MM determined by JC-1 and MitoTracker Orange staining. Representative confocal microscopic images of JC-1 stained PC3 (a) and 22Rv1 (b) cell lines. Quantitative FACS measurement of JC-1 (c) and MitoTracker Orange staining (d) in 2D- and 3D-cultured 22Rv1 cells. Results showed that cells in prostatospheroids displayed higher MMP and MM than their 2D-cultured adherent cells. e and f Detection of MMP (e) and MM (f) in SORE6-sorted DU145 and LNCaP cells. Results showed that SORE6-positive DU145 and LNCaP cells exhibited higher MMP and MM as compared to their corresponding negative cells.

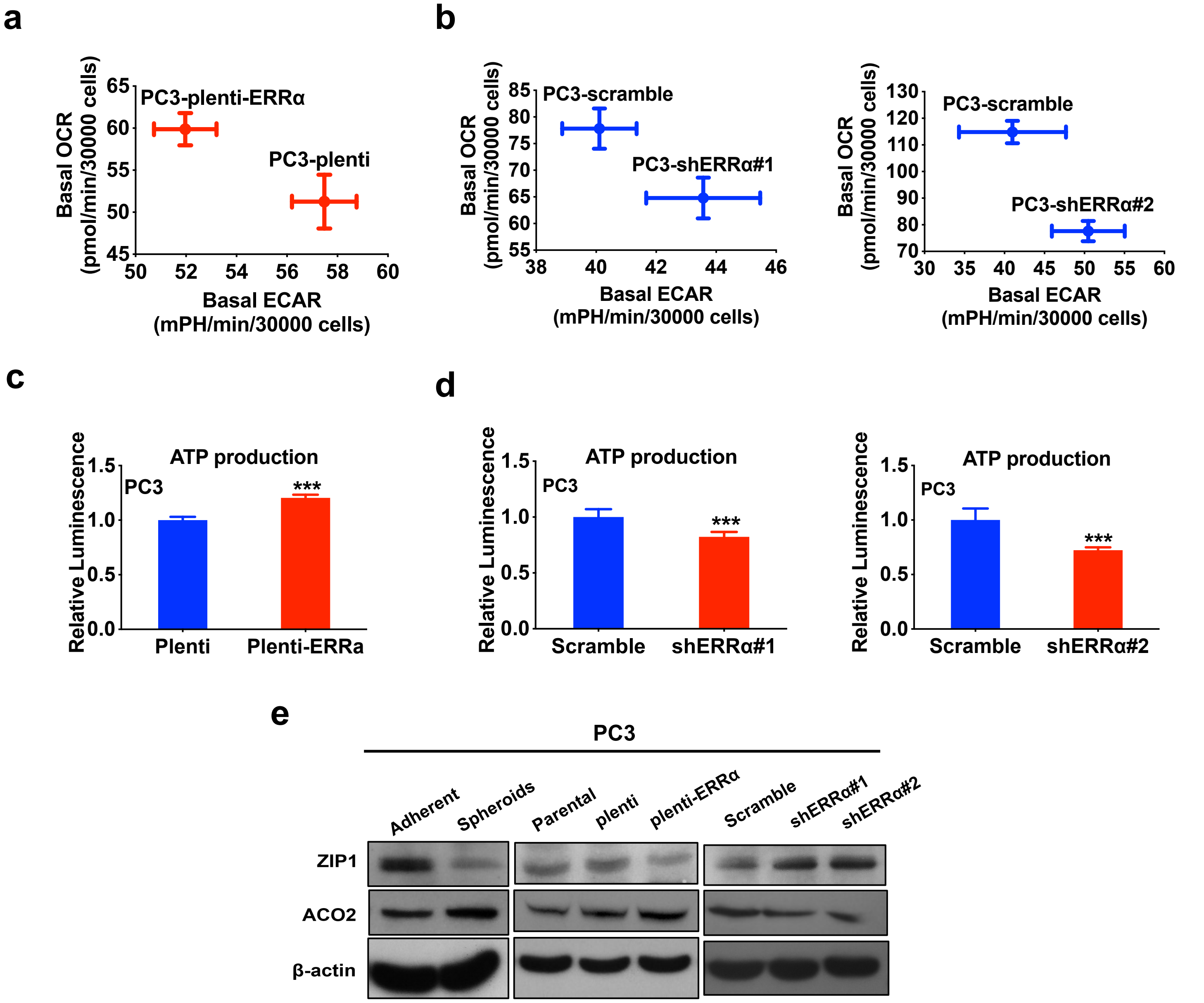

Fig. S2 a-b Metabolic Switching assessment and ATP production measurement. Results showed that PC3-plenti-ERRα infectants exhibited higher OCR but lower ECAR (a), as well as higher ATP production (c) than their empty vector controls, whereas PC3-shERRα#1 and PC3-shERRα#2 infectants exhibited lower OCR but higher ECAR (b), as well as lower ATP production (d) than their scrambles. e Immunoblot analyses of ZIP1 and ACO2 protein expression. Left: 3D-cultured PC3-spheroids expressed significant lower levels of ZIP1 and higher levels of ACO2 than adherent PC3 cells. Middle: PC3-plenti-ERRα infectants expressed significant decreased levels of ZIP1 and increased levels of ACO2 as compared to PC3-plenti infectants. Right: PC3-shERRα#1 and PC3-shERRα#2 infectants exhibited significant increased levels of ZIP1 and decreased levels of ACO2 compared with PC3-scramble infectants. Results are presented as mean ± SD of three independent experiments. *** < 0.001 versus corresponding empty vector plenti or scramble controls.

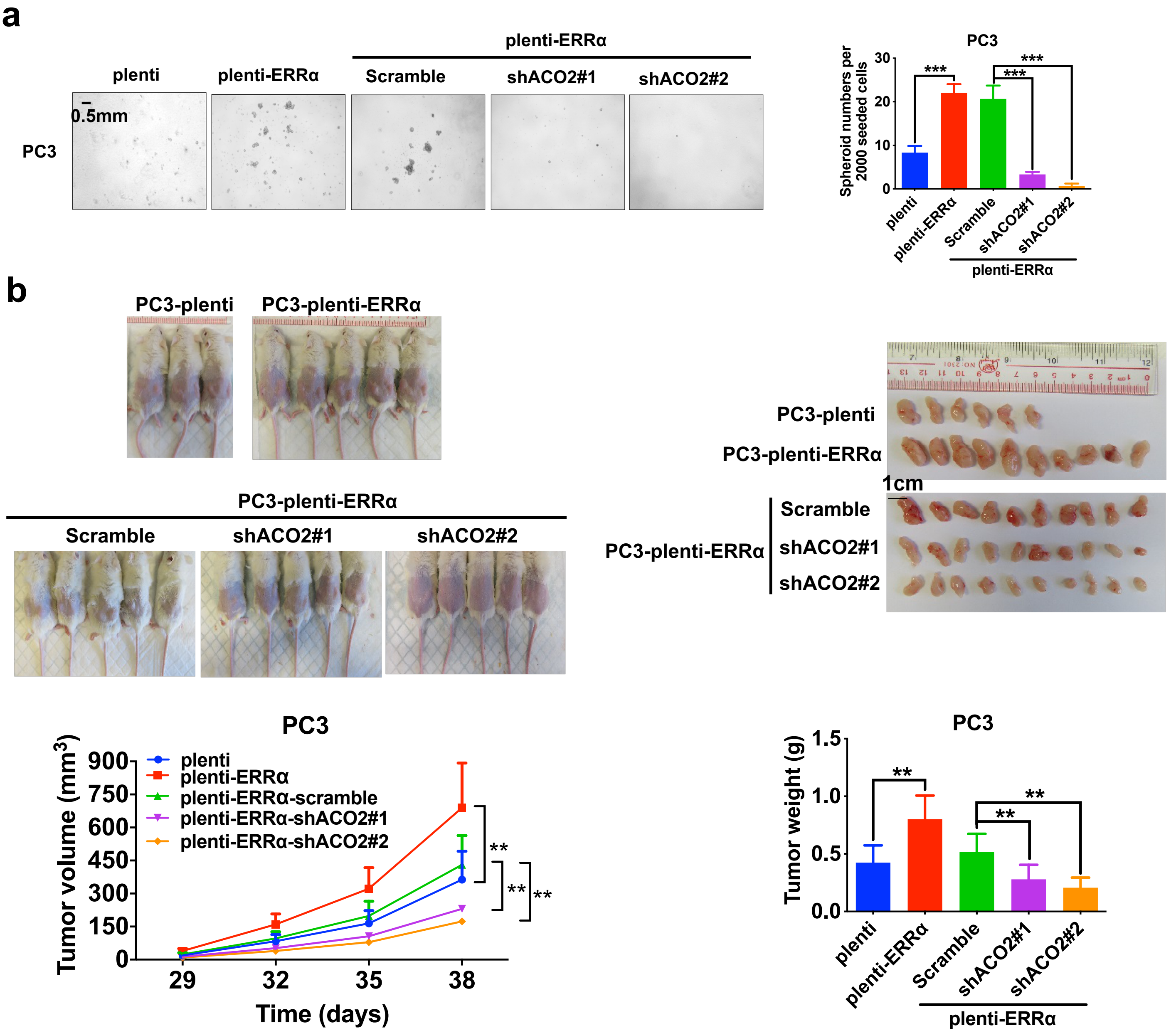

Fig. S3 a 3D-cultured spheroid formation assay. Left: Representative images of spheroids formed by PC3-plenti, PC3-plenti-ERRα, PC3-plenti-ERRα-scramble, PC3-plenti-ERRα-shACO2#1 and PC3-plenti-ERRα-shACO2#2 infectants. Right: Quantification analyses of spheroids formed. Results showed that PC3-plenti-ERRα infectants formed more and larger spheroids than PC3-plenti infectants, which could be abolished by ACO2 knockdown. **b** *In vivo* tumorigenicity assay of PC3-plenti, PC3-plenti-ERRα, PC3-plenti-ERRα-scramble, PC3-plenti-ERRα-scramble-shACO2#1 and PC3-plenti-ERRα-shACO2#2 infectants. Top left: Representative images of host mice bearing tumors formed by the injected infectants; Top right: Images of dissected tumors formed by the infectants. Bottom left: Graph shows the growth curve of xenograft tumors formed by the infectants. Bottom right: Measurement of wet weights of tumors formed by the infectants. PC3-plenti-ERRα infectants formed larger tumors than PC3-plenti cells. PC3-plenti-ERRα-shACO2#1 and PC3-plenti-ERRα-shACO2#2 infectants generated smaller tumors at slower rates than control PC3-plenti-ERRα-scramble. Results are presented as mean ± SD of three independent experiments. $^{**}P < 0.01$, $^{***} < 0.001$ versus corresponding empty vector plenti or scramble controls.

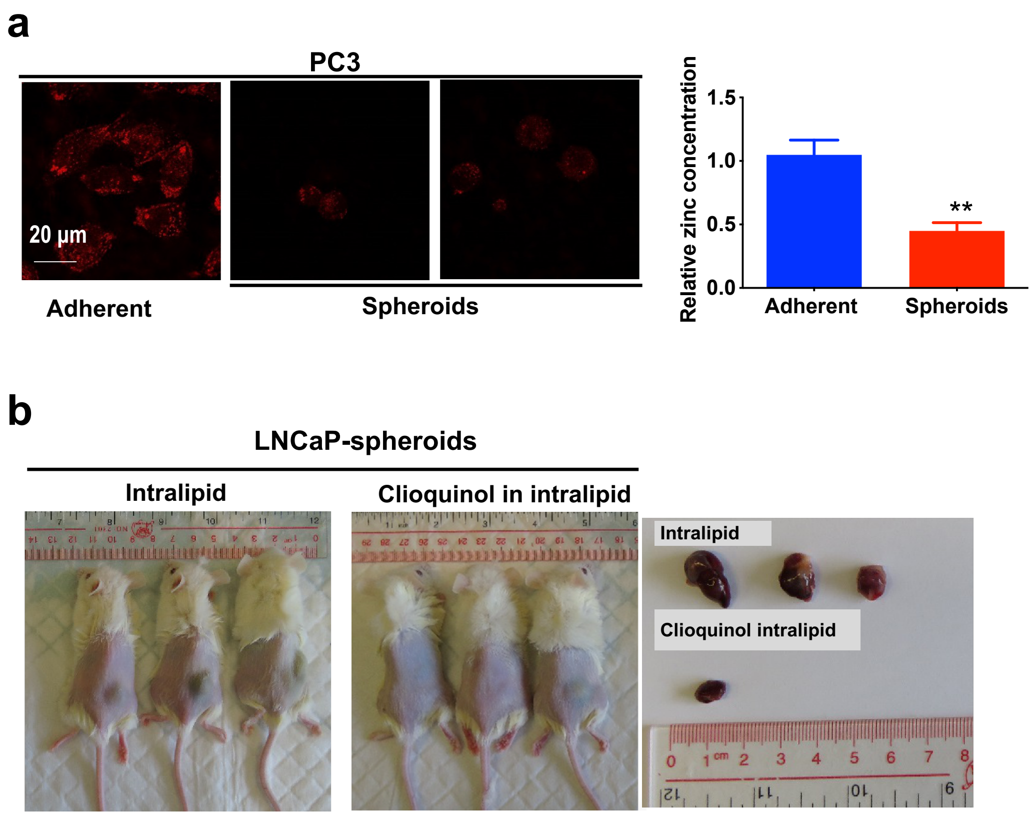

Fig. S4 a Intracellular zinc levels measured using zinquin ethyl ester. Left: Representative images of zinquin ethyl ester staining examined by confocal microscopy in PC3 cells grown under 2D- and 3D-cultured conditions. Right: Quantitative analyses of zinquin ethyl ester staining by microplate reader. Results showed that PC3-spheroids displayed lower intracellular zinc levels as compared to their corresponding 2D-cultured adherent cells. **b** Tumorigenicity of LNCaP-spheroids upon *in vivo* treatment with clioquinol or vehicle (intralipid). Following inoculation with LNCaP-spheroids, the host mice were randomly assigned to intraperitoneal injections of clioquinol (dissolved in 20% intralipid and 33 mg/Kg in 200 µl) or vehicle every other day for two months. Left: Representative images of host mice bearing tumors formed by the LNCaP-spheroids upon treatment with clioquinol or vehicle. Right: Images of dissected tumors. Results showed that LNCaP spheroids formed bigger xenograft tumors in all (3/3) host mice upon treatment with vehicle, whereas smaller xenograft tumors in only 1/3 of host mice upon treatment with clioquinol. **$P < 0.01$ versus adherent 2D-cultured cells.

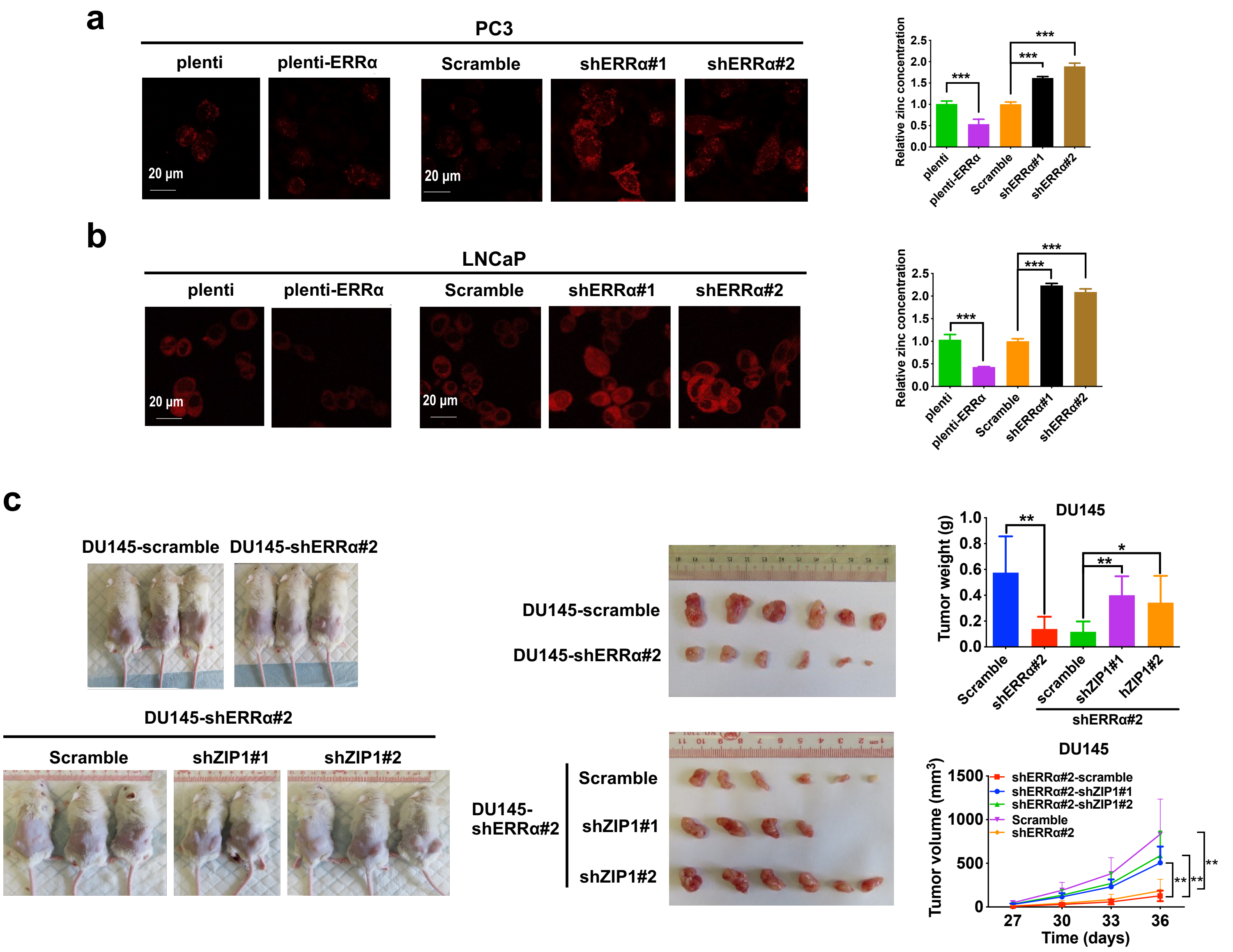

Fig. S5 a and b Intracellular zinc levels measured using zinquin ethyl ester. a Left: Representative images of zinquin ethyl ester staining in PC3-plenti and PC3-plenti-ERRα as well as PC3-scramble, PC3-shERRα#1 and #2 infectants. Right: Quantitative analyses of zinquin ethyl ester staining. Results showed that PC3-plenti-ERRα cells exhibited lower intracellular zinc levels as compared to PC3-plenti cells, whereas PC3-shERRα#1 and #2 infectants displayed higher intracellular zinc levels compared with PC3-scramble infectants. **b** Left : Representative images of zinquin ethyl ester staining in LNCaP-plenti and LNCaP-plenti-ERRα as well as LNCaP-scramble, LNCaP-shERRα#1 and #2 infectants. Right : Quantitative analyses of zinquin ethyl ester staining. Results showed that LNCaP-plenti-ERRα cells exhibited lower intracellular zinc levels as compared to LNCaP-plenti cells, whereas LNCaP -shERRα#1 and #2 infectants displayed higher intracellular zinc levels compared with LNCaP -scramble infectants. **c** *In vivo* tumorigenicity assay. Left: Representative images of host mice bearing tumors formed by the injected DU145-scramble, DU145-shERRα#2, DU145-shERRα#2-shZIP1#1 and DU145-shERRα#2-shZIP1#2 infectants; Middle: Images of dissected tumors formed by the infectants. Right top: Measurement of wet weights of tumors formed by the infectants. Right bottom: Graph shows the growth curve of xenograft tumors formed by the infectants. DU145-shERRα#2 infectants formed tumors in smaller sizes at slower rates than those of DU145-scramble infectants; DU145-shERRα#2-shZIP1#1 and DU145-shERRα#2-shZIP1#2 infectants formed xenograft tumors in larger sizes at faster rates than those of DU145-shERRα#2 infectants. Results are presented as mean ± SD of three independent experiments. $^{**}P < 0.01$, $^{***} < 0.001$ versus corresponding empty vector plenti or scramble controls.
