# Supplementary material for: Estrogen-related receptor alpha (ERRα) controls the stemness and cellular energetics of prostate cancer cells via its direct regulation of citrate metabolism and zinc transportation
Source: Cell Death Dis. 2025 Mar 5;16(1):154. doi: 10.1038/s41419-025-07460-z (PMC11882781; doi:10.1038/s41419-025-07460-z)
Supplement: Supplementary file 3 — Unchopped Whole Blots [file 41419_2025_7460_MOESM3_ESM.pdf]

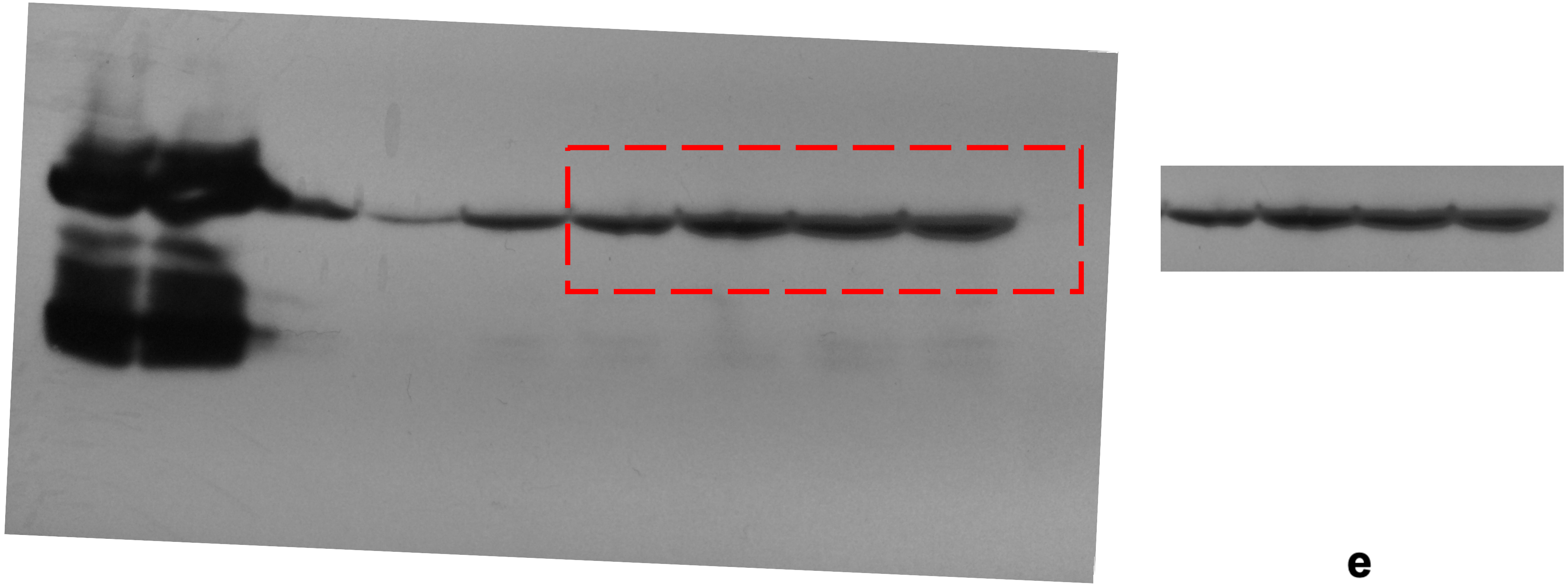

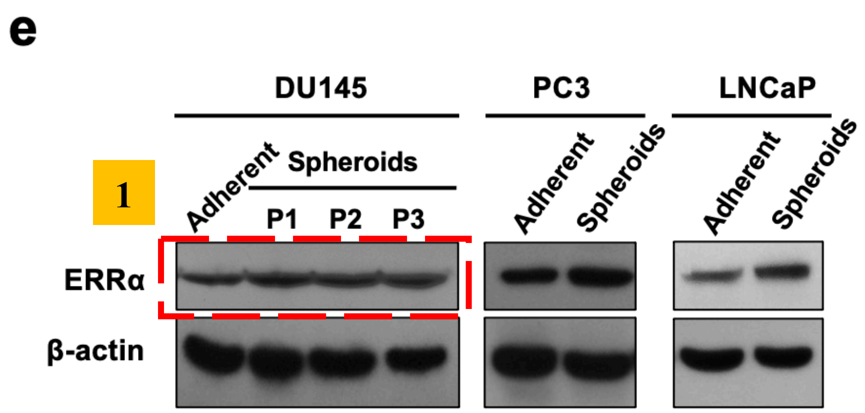
e
DU145
PC3
LNCaP
Adherent
Spheroids
P1
P2
P3
Adherent
Spheroids
Adherent
Spheroids
1
ERRα
β-actin

Fig. 1e

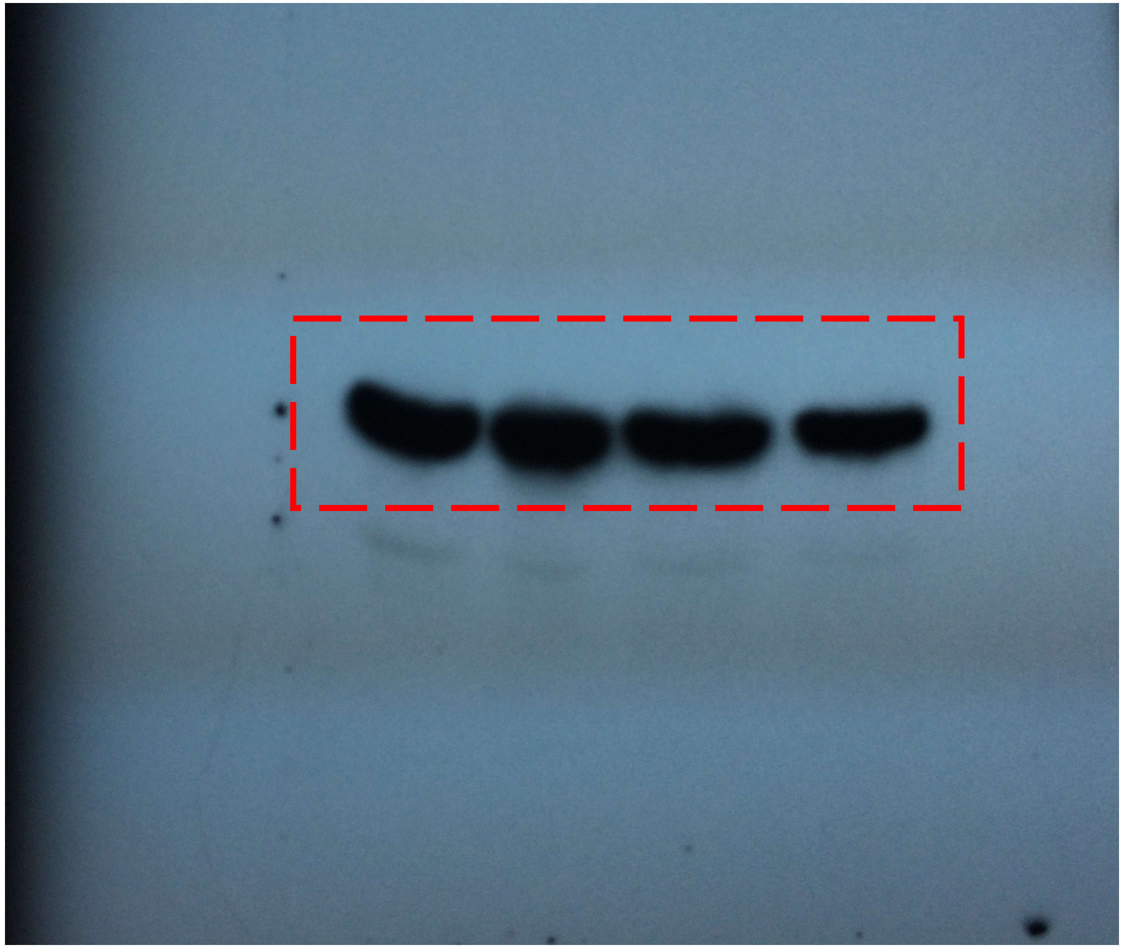

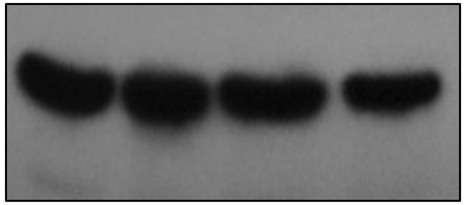

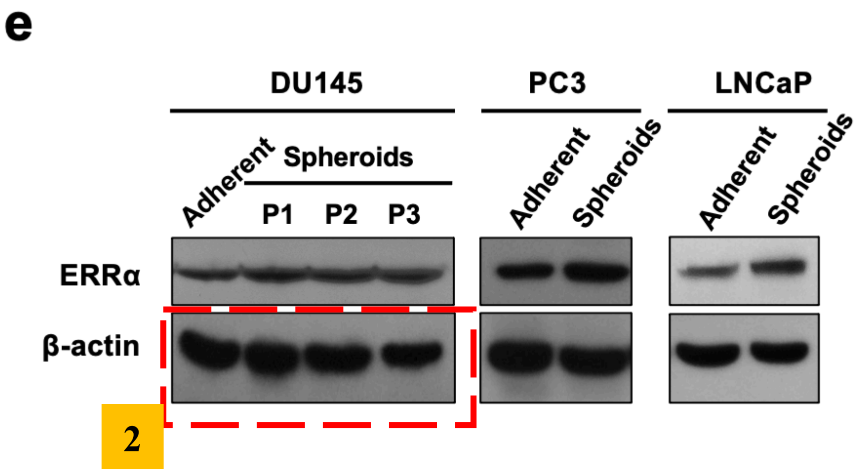
e
DU145
PC3
LNCaP
Adherent
Spheroids
P1
P2
P3
Adherent
Spheroids
Adherent
Spheroids
ERRα
β-actin
2

Fig. 1e

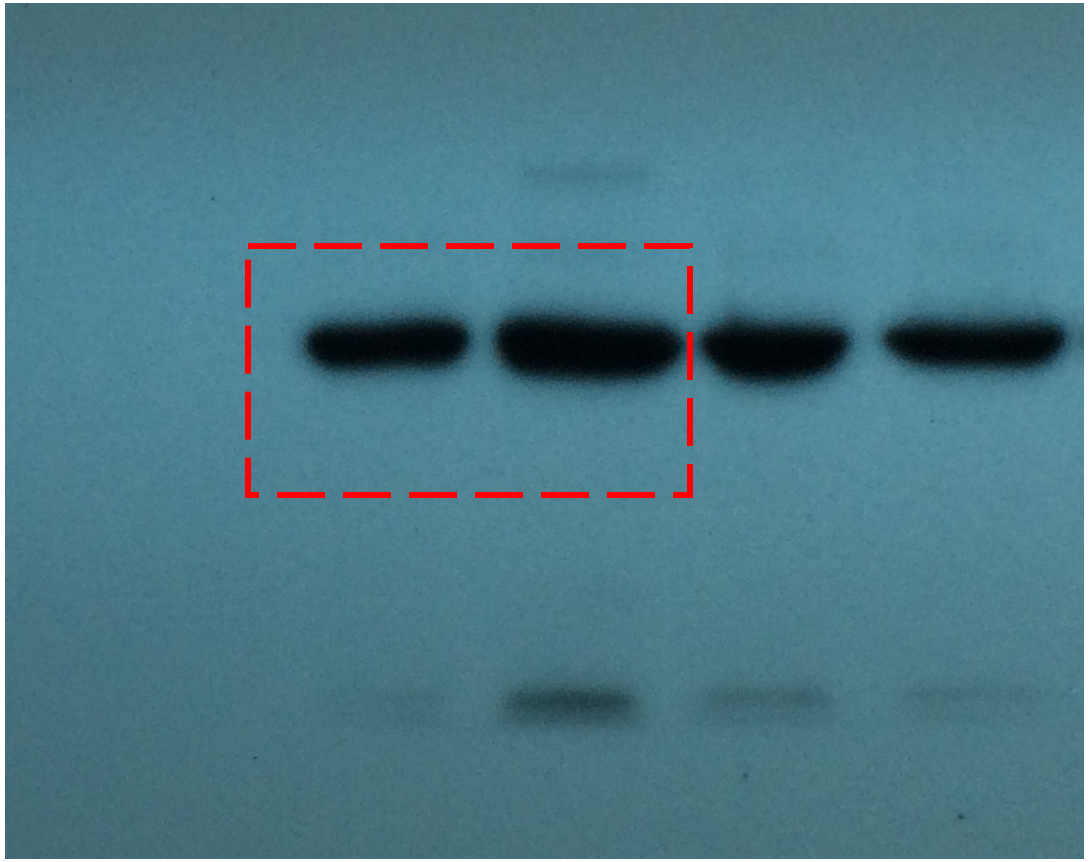

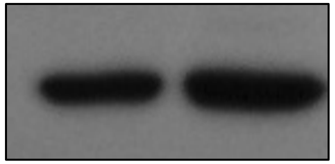

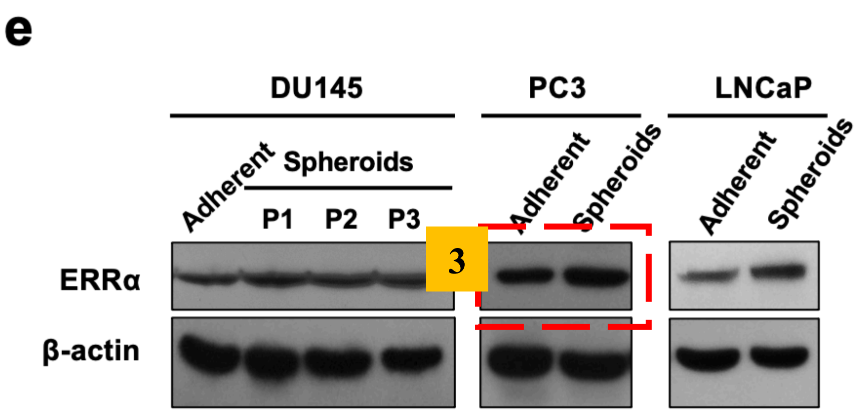
e
DU145
PC3
LNCaP
Adherent
Spheroids
P1
P2
P3
Adherent
Spheroids
Adherent
Spheroids
3
ERRα
β-actin

Fig. 1e

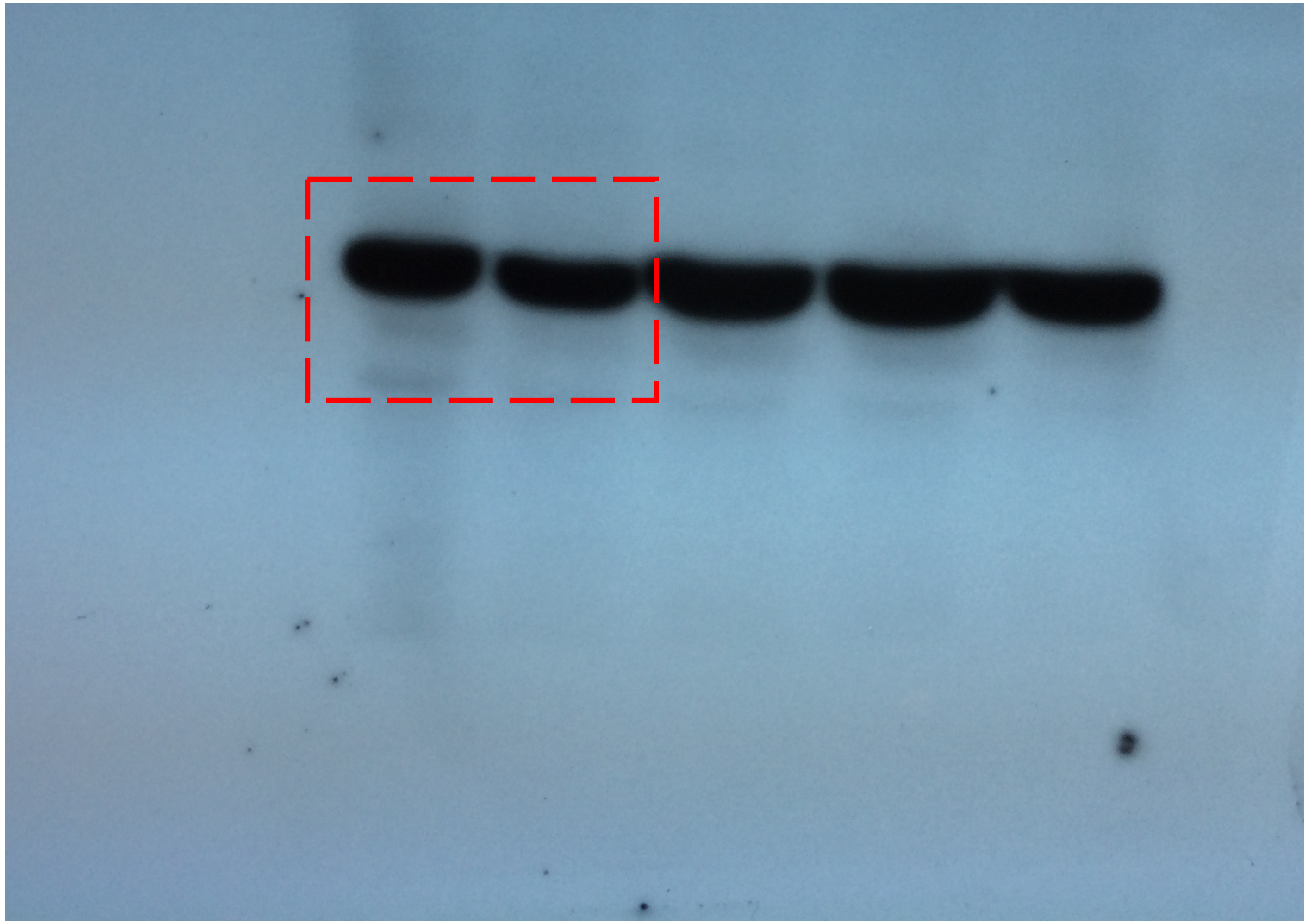

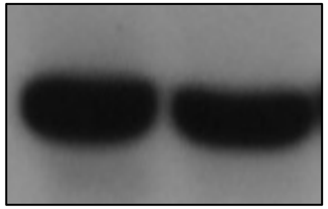

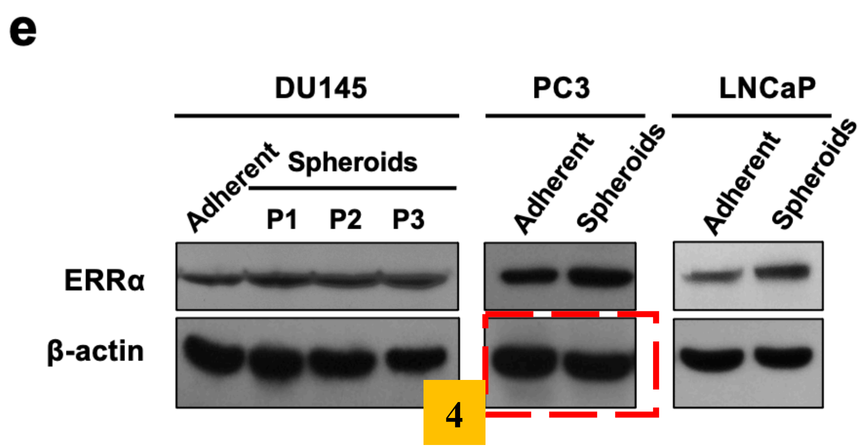
e
DU145
PC3
LNCaP
Adherent
Spheroids
P1
P2
P3
Adherent
Spheroids
Adherent
Spheroids
ERRα
β-actin
4

Fig. 1e

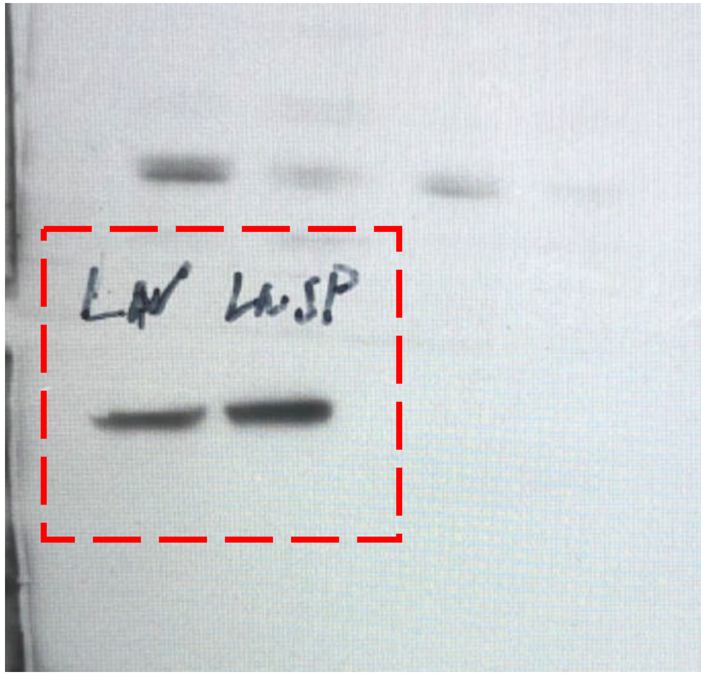

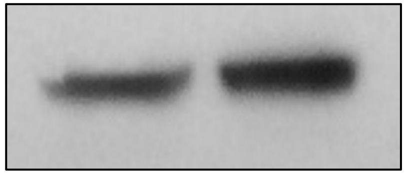

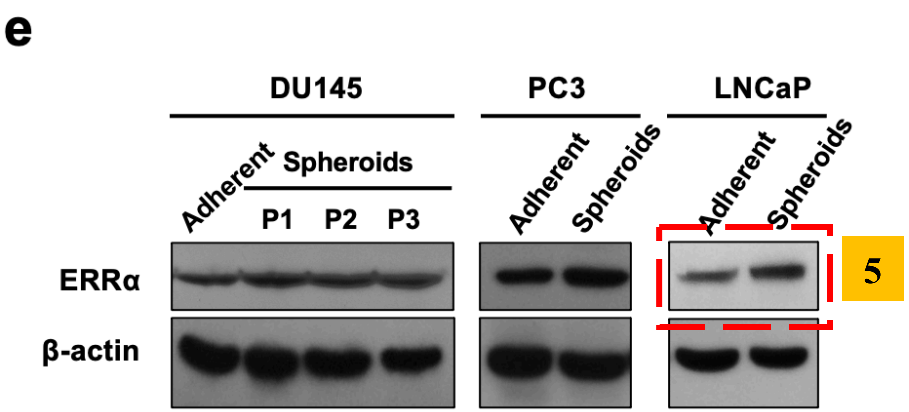
e
DU145
PC3
LNCaP
Adherent
Spheroids
P1
P2
P3
Adherent
Spheroids
Adherent
Spheroids
ERRα
β-actin
5

Fig. 1e

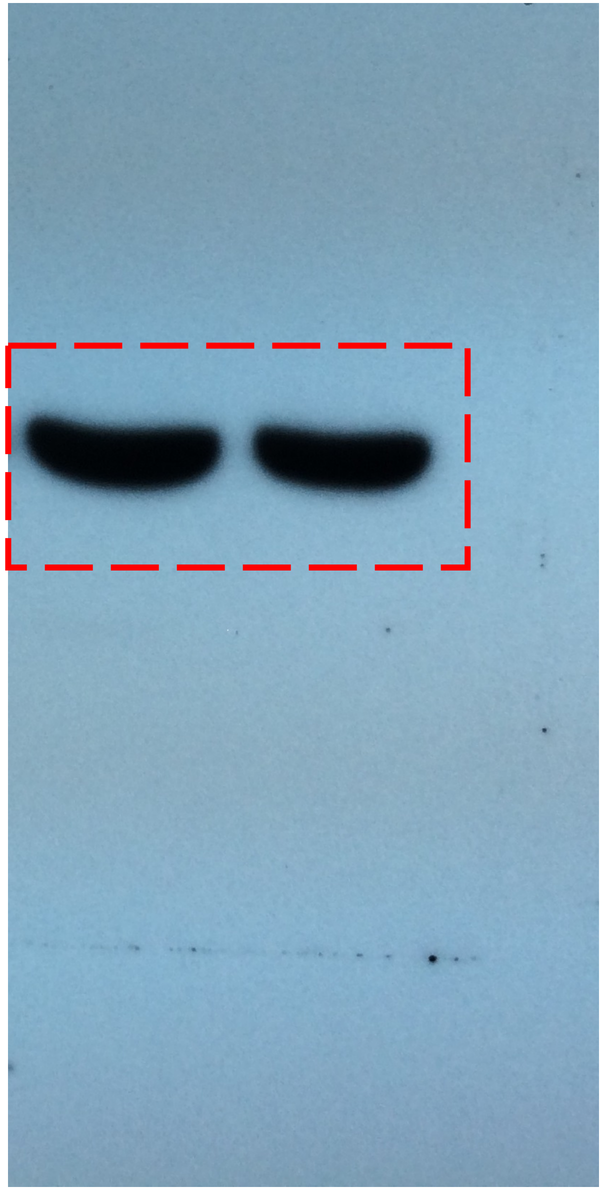

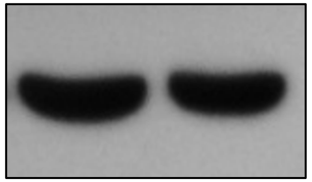

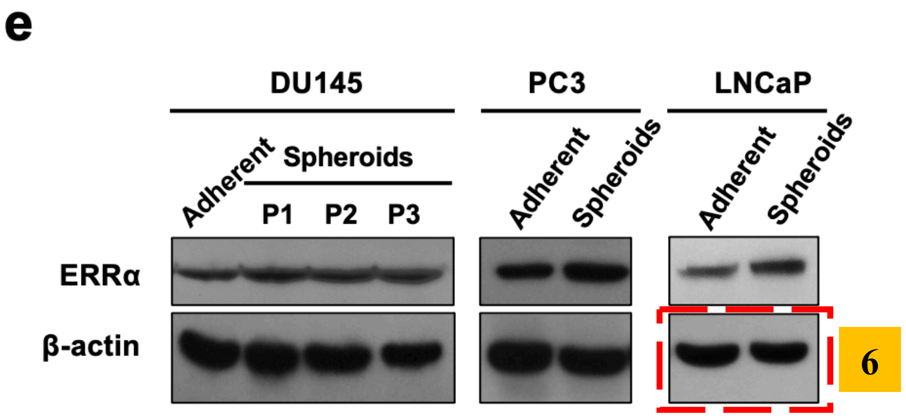
e
DU145
PC3
LNCaP
Adherent
Spheroids
P1
P2
P3
Adherent
Spheroids
Adherent
Spheroids
ERRα
β-actin
6

Fig. 1e

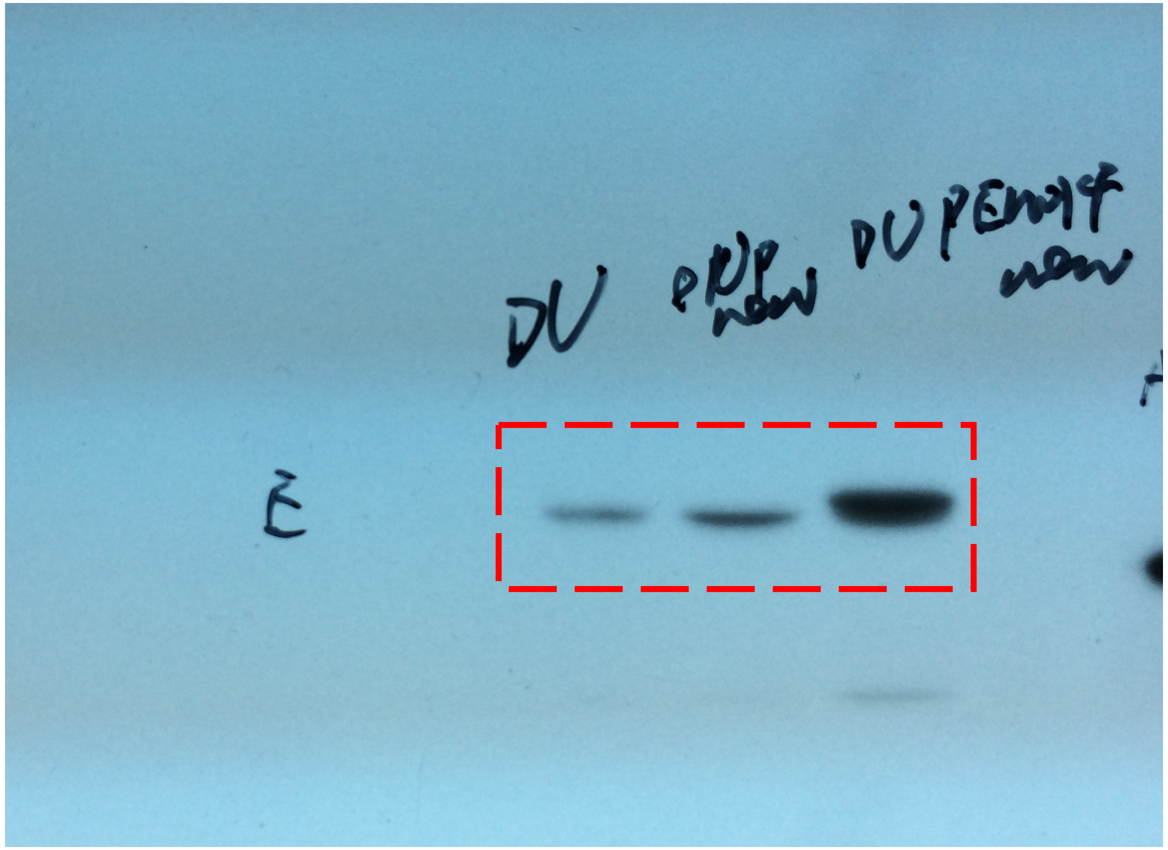
DU

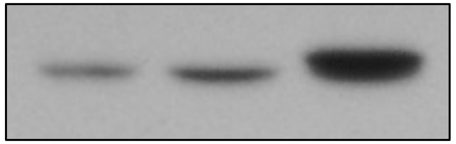

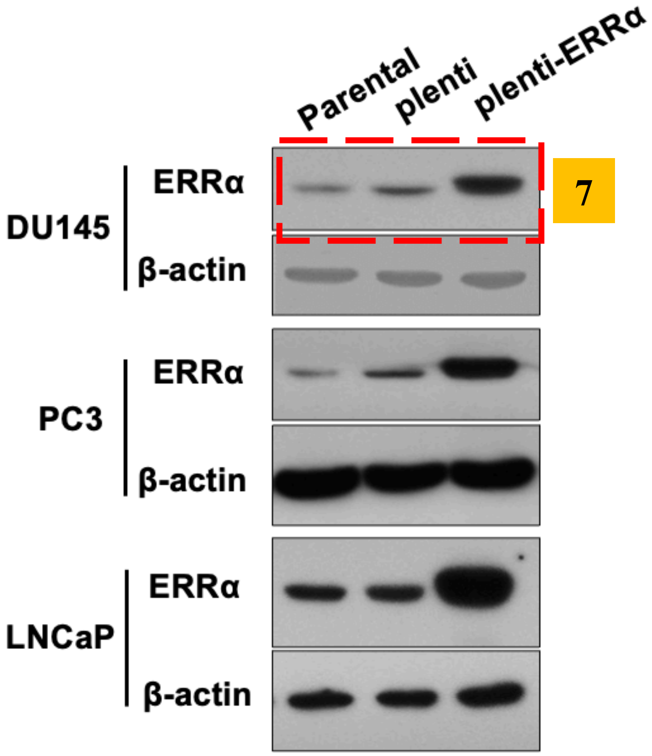
Parental
plenti
plenti-ERRα
DU145
ERRα
β-actin
PC3
ERRα
β-actin
LNCaP
ERRα
β-actin
7

Fig. 2a

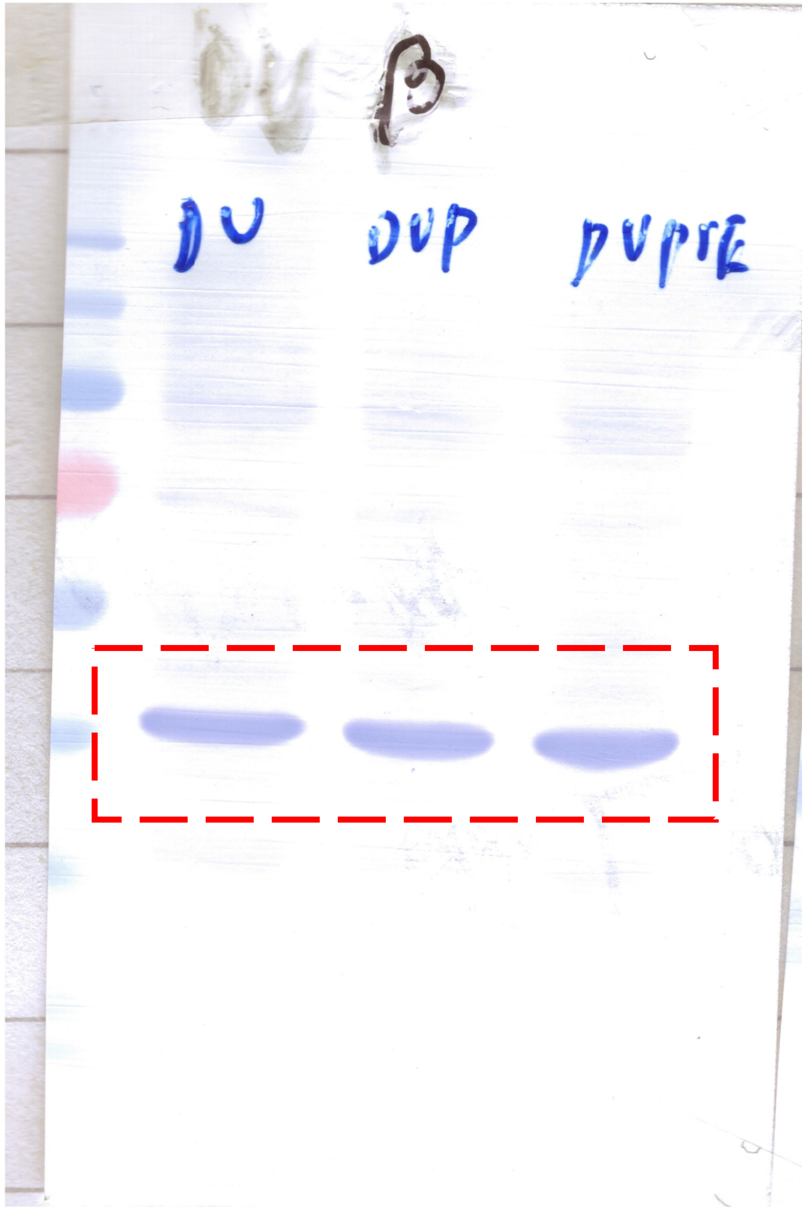

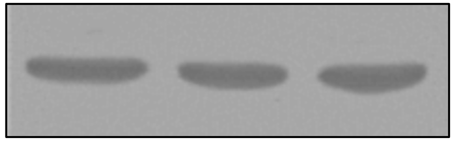

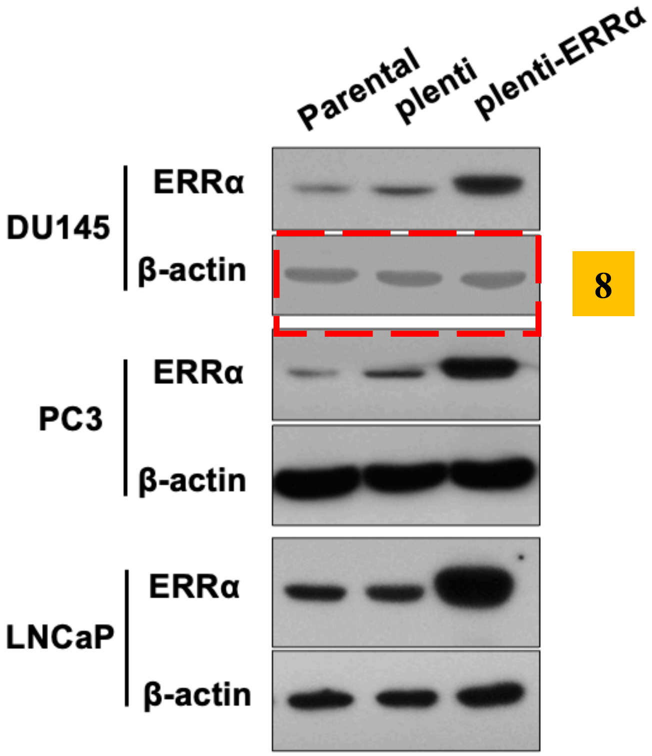
Parental
plenti
plenti-ERRα
DU145
ERRα
β-actin
PC3
ERRα
β-actin
LNCaP
ERRα
β-actin

8

Fig. 2a

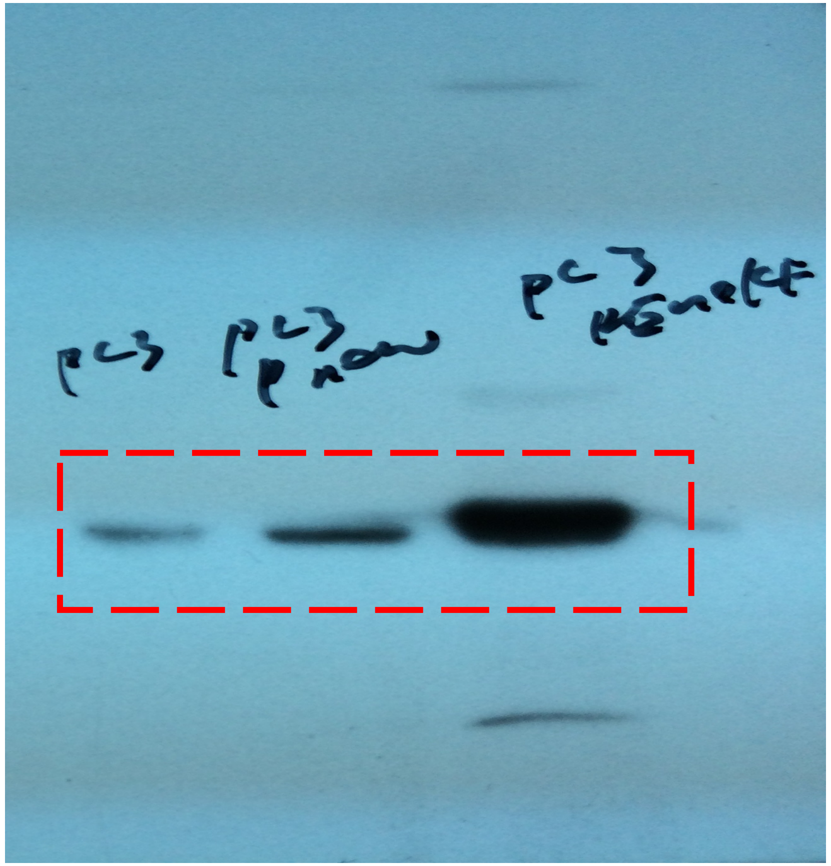

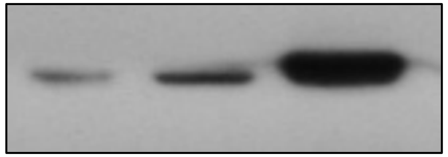

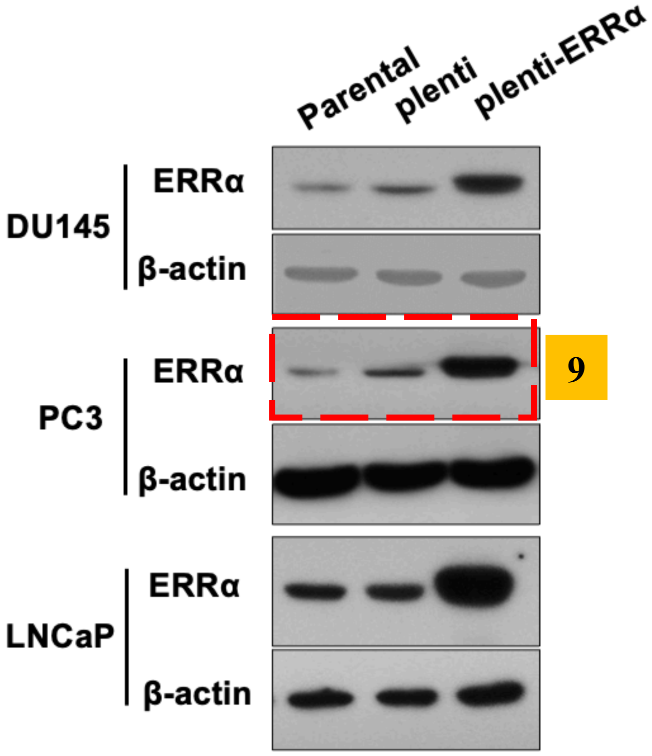
Parental
plenti
plenti-ERRα
DU145
ERRα
β-actin
PC3
ERRα
β-actin
LNCaP
ERRα
β-actin
9

Fig. 2a

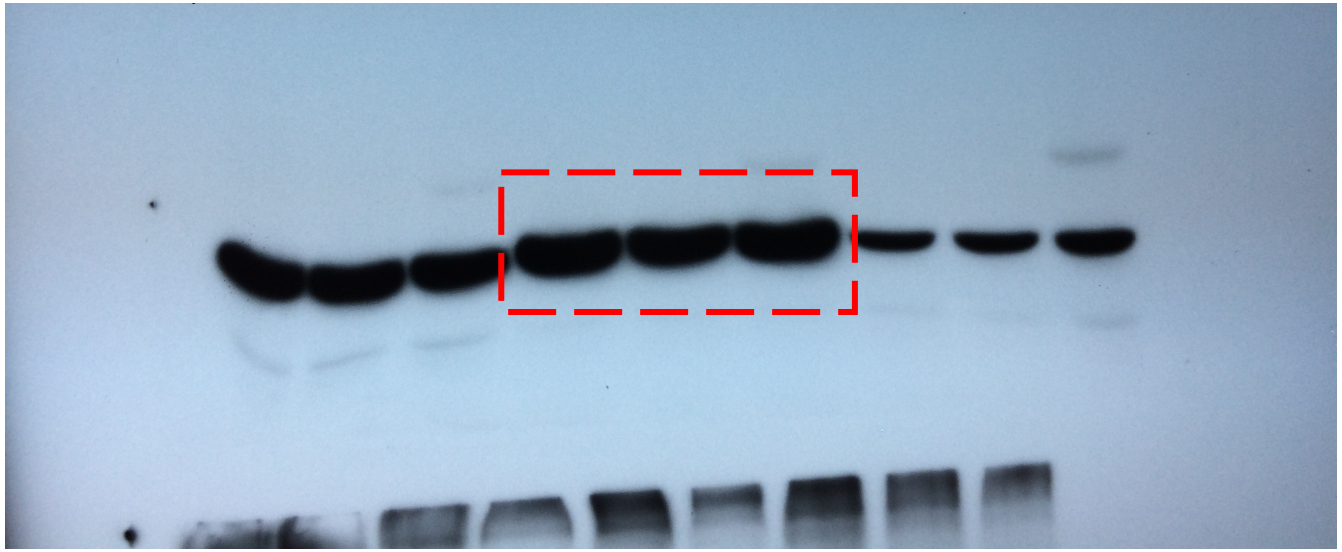

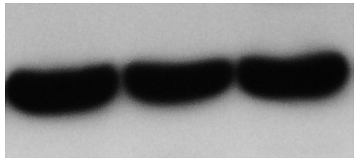

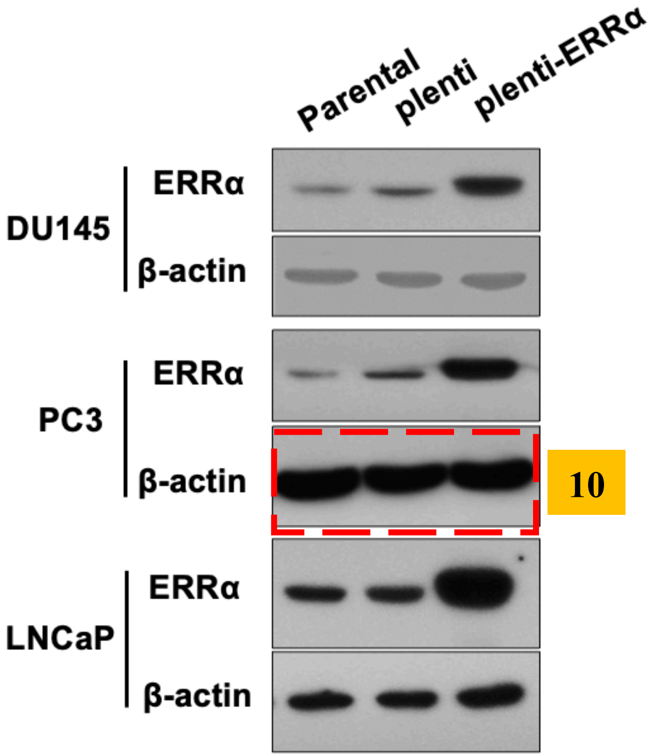
Parental
plenti
plenti-ERRα
DU145
ERRα
β-actin
PC3
ERRα
β-actin
10
LNCaP
ERRα
β-actin

Fig. 2a

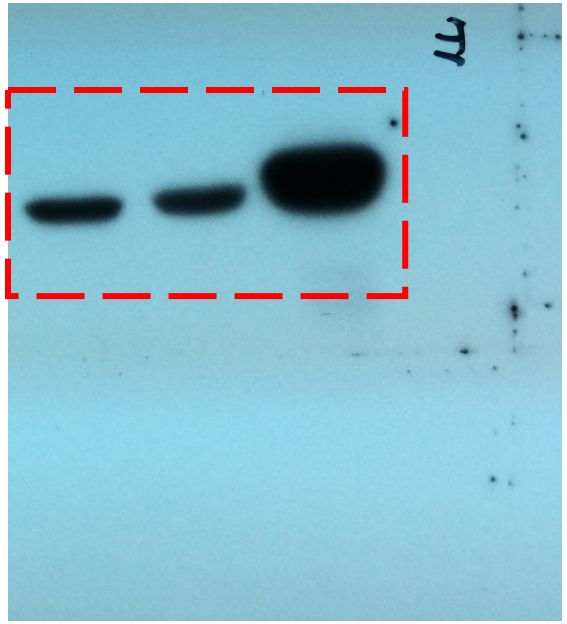

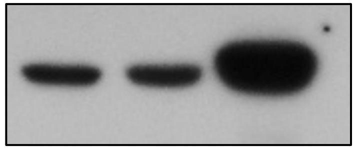

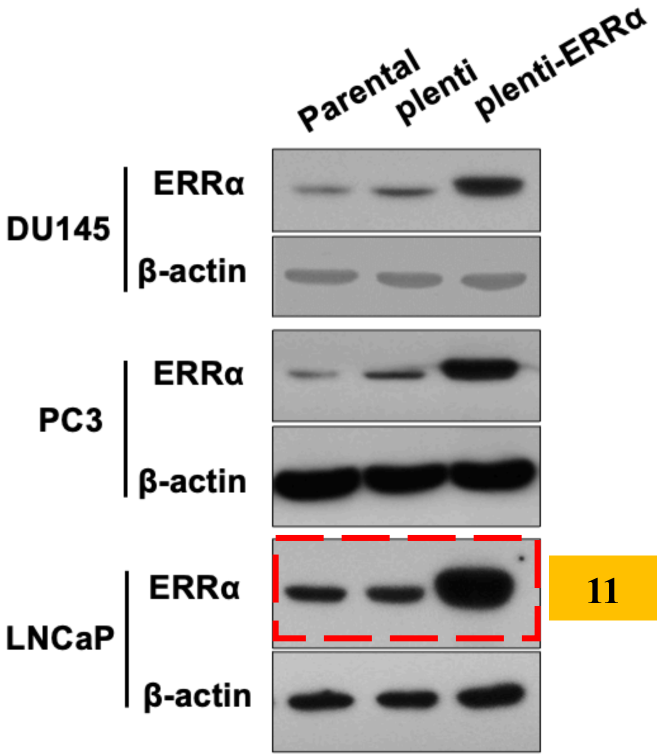
Parental
plenti
plenti-ERRα
DU145
ERRα
β-actin
PC3
ERRα
β-actin
LNCaP
ERRα
β-actin

11

Fig. 2a

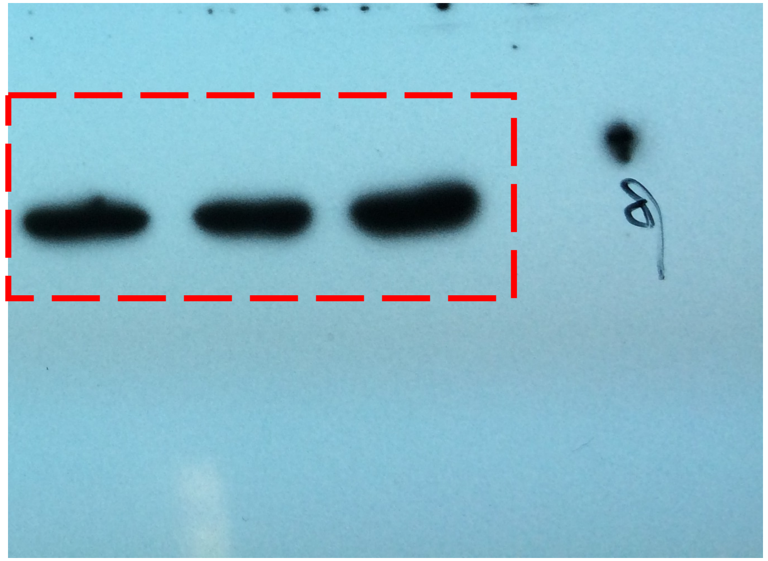

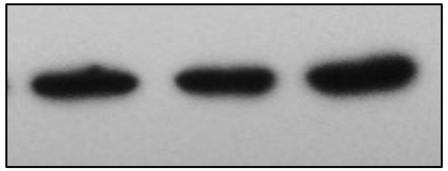

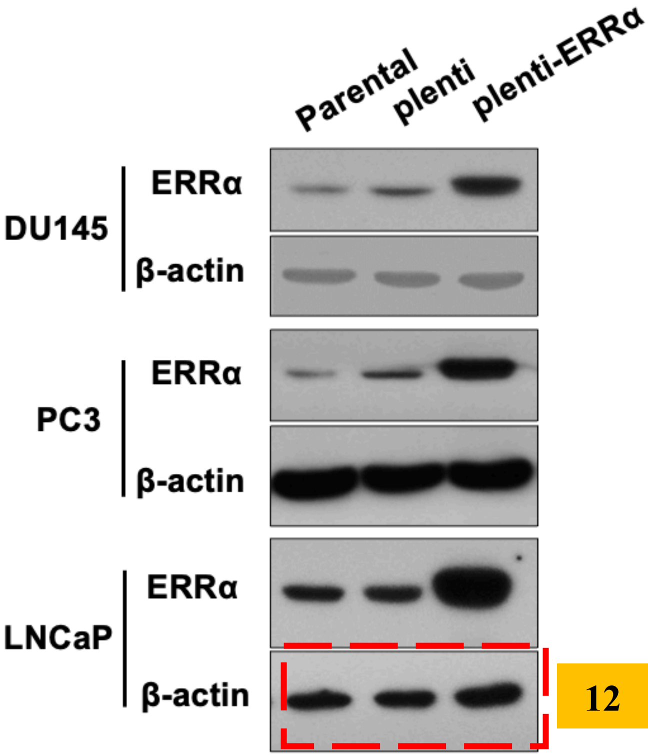
Parental
plenti
plenti-ERRα
DU145
ERRα
β-actin
PC3
ERRα
β-actin
LNCaP
ERRα
β-actin

Fig. 2a

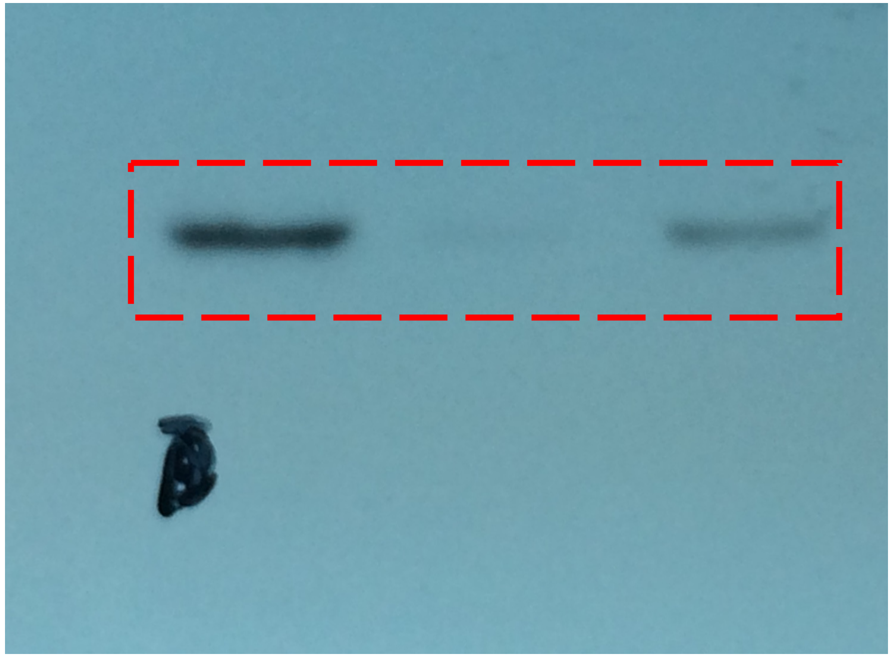

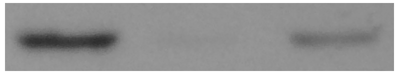

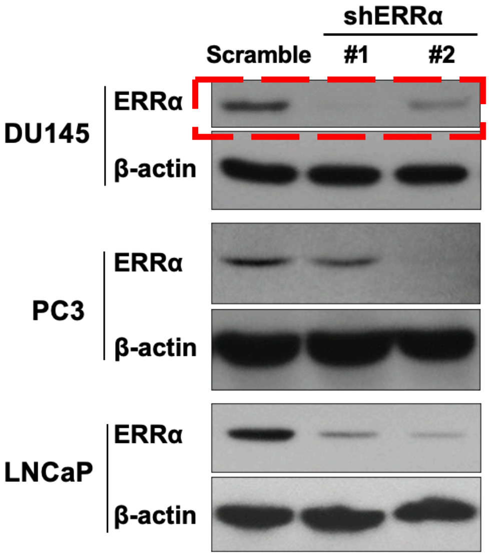
shERRα
Scramble
#1
#2
DU145
ERRα
β-actin
PC3
ERRα
β-actin
LNCaP
ERRα
β-actin

Fig. 2e

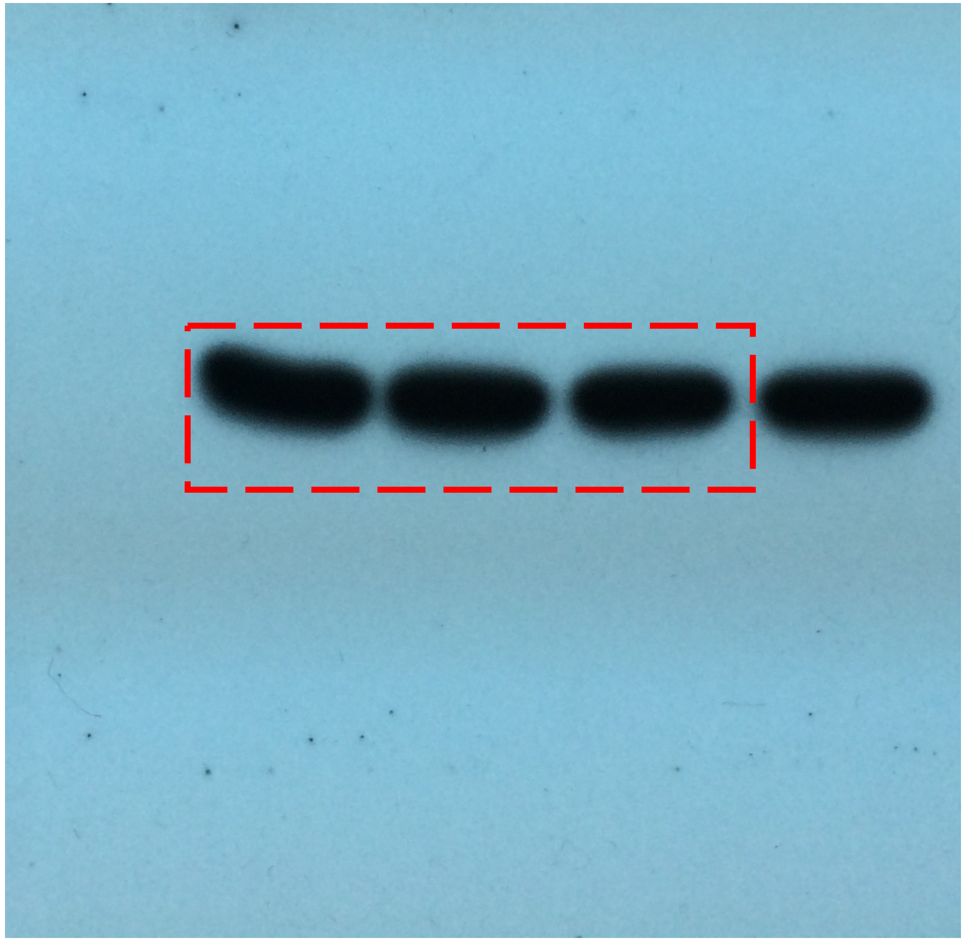

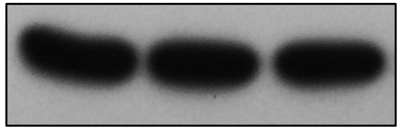

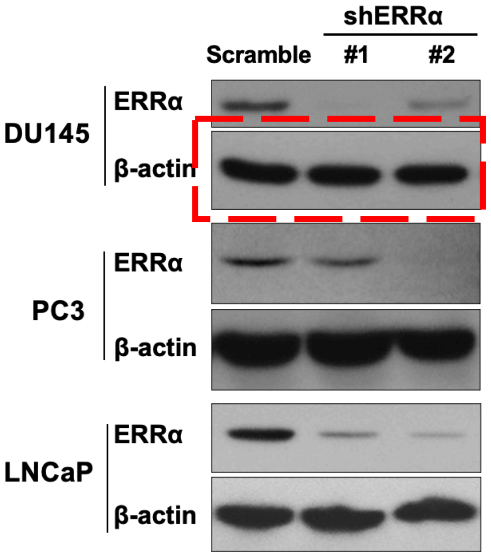
shERRα
Scramble
#1
#2
DU145
ERRα
β-actin
PC3
ERRα
β-actin
LNCaP
ERRα
β-actin

14

Fig. 2e

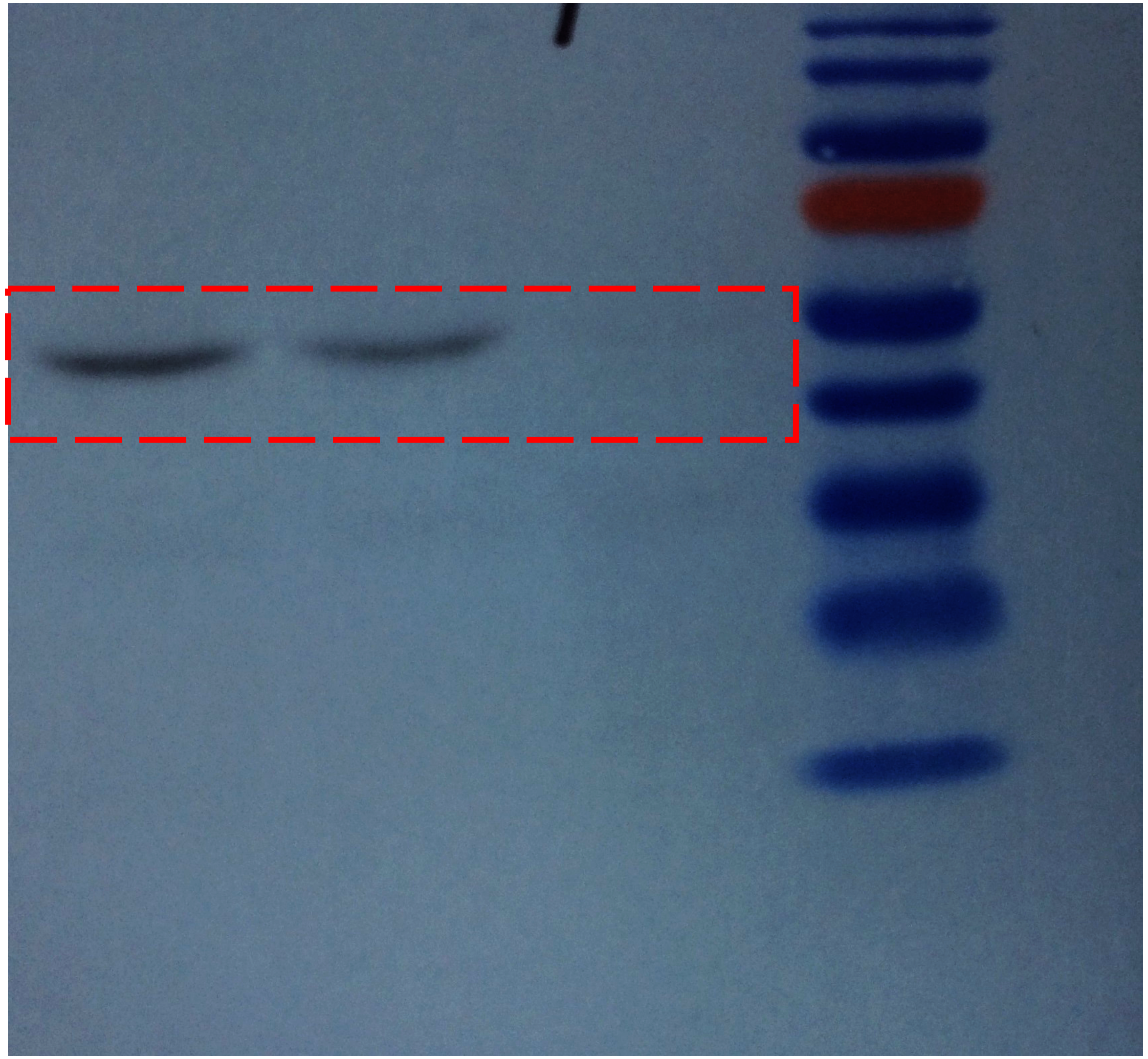

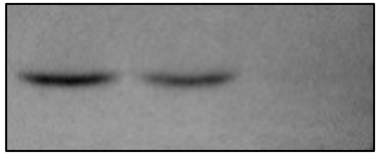

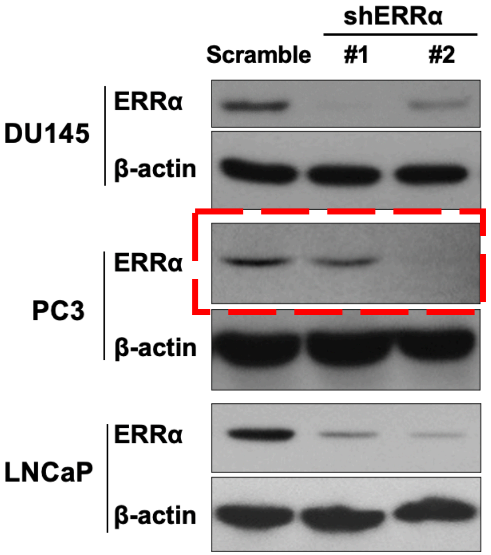

shERRα
Scramble
#1
#2
DU145
ERRα
β-actin
PC3
ERRα
β-actin
LNCaP
ERRα
β-actin

15

Fig. 2e

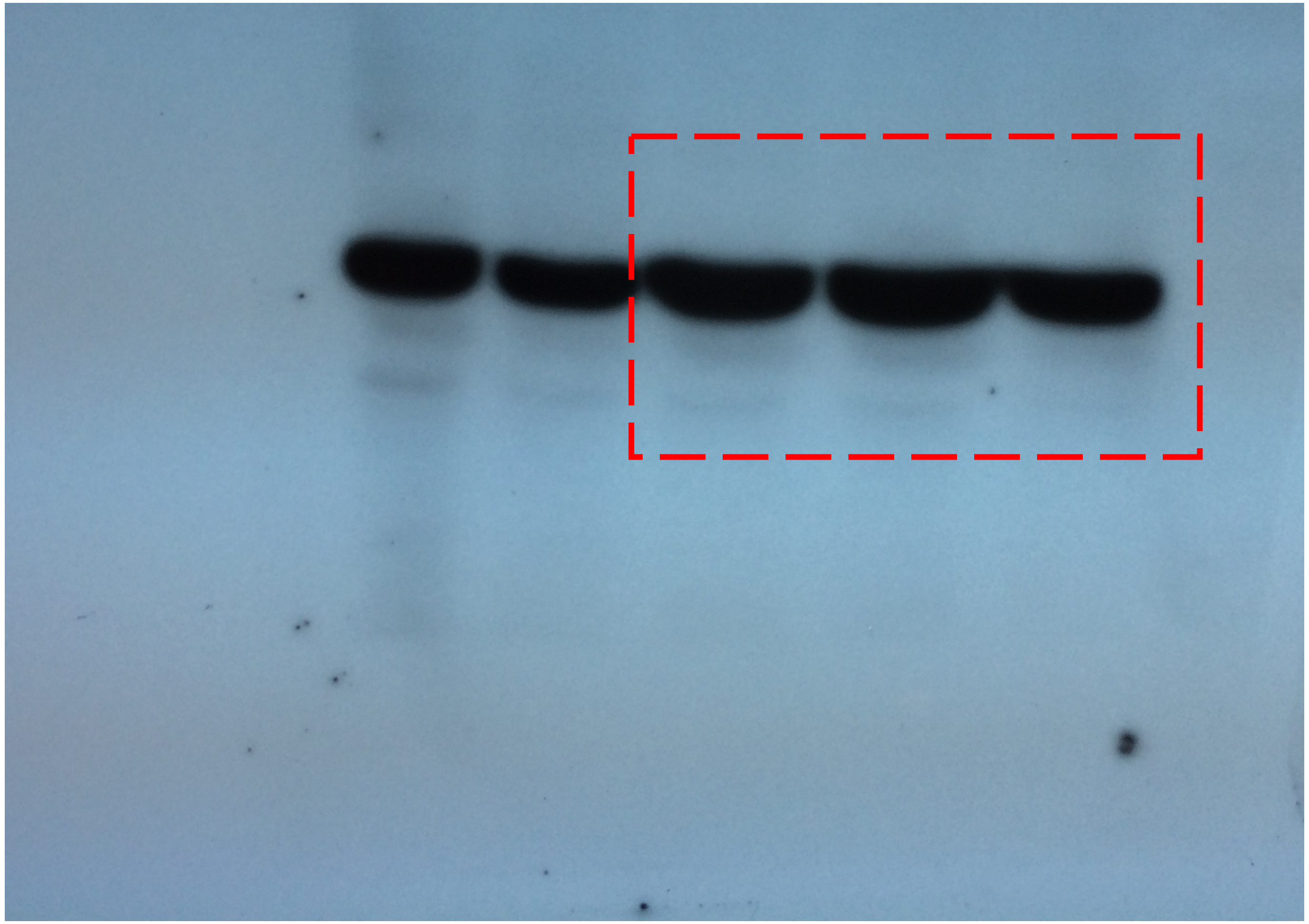

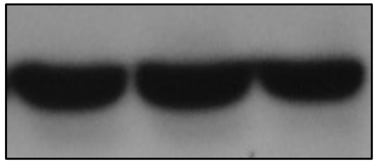

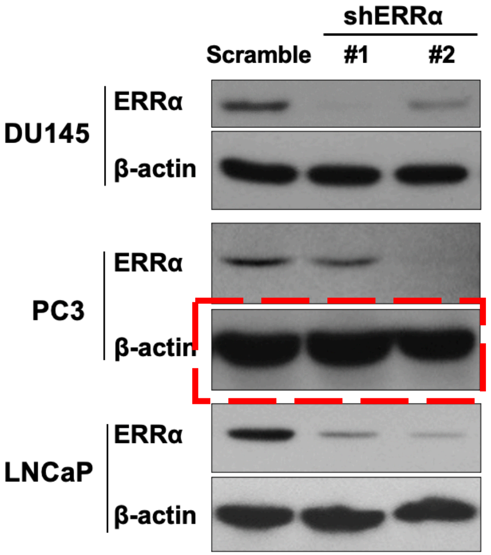
shERRα
Scramble
#1
#2
DU145
ERRα
β-actin
PC3
ERRα
β-actin
LNCaP
ERRα
β-actin

16

Fig. 2e

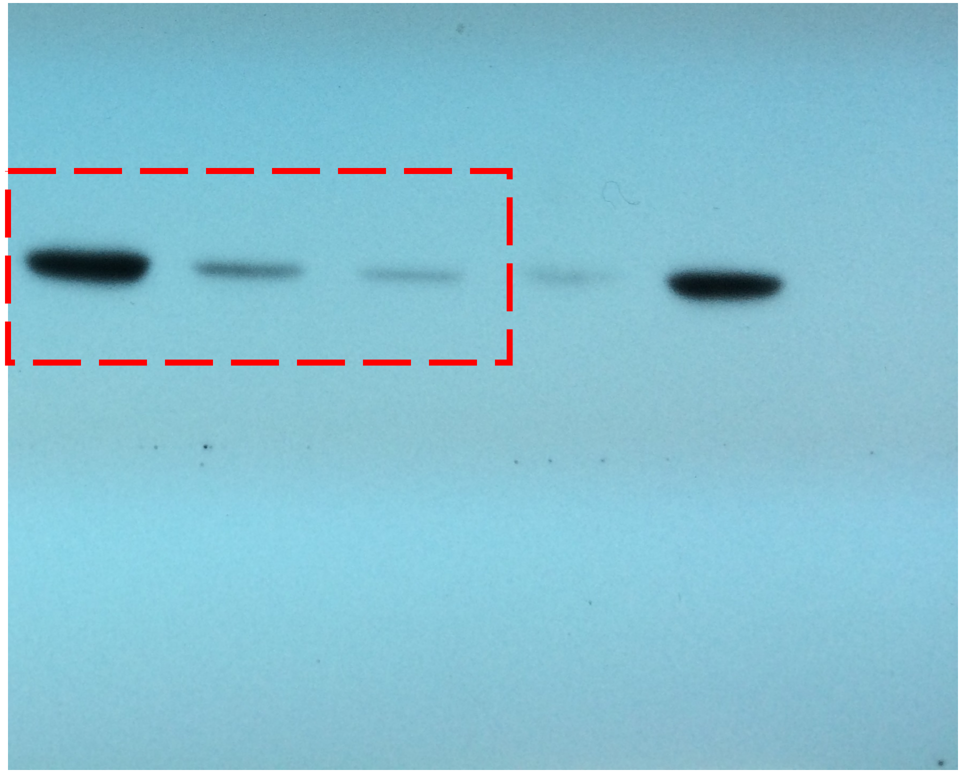

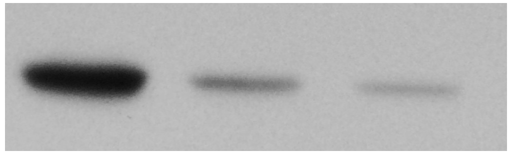

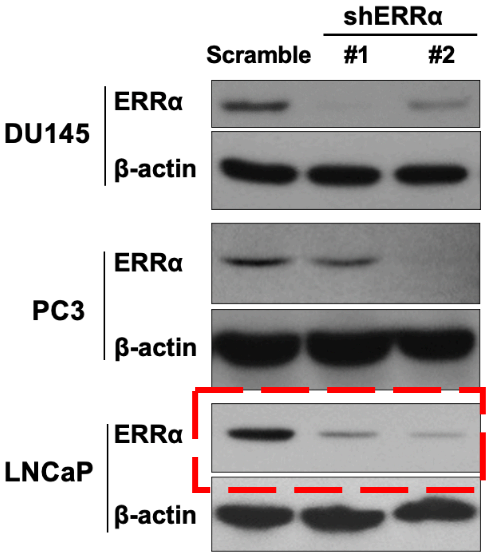
shERRα
Scramble
#1
#2
DU145
ERRα
β-actin
PC3
ERRα
β-actin
LNCaP
ERRα
β-actin

17

Fig. 2e

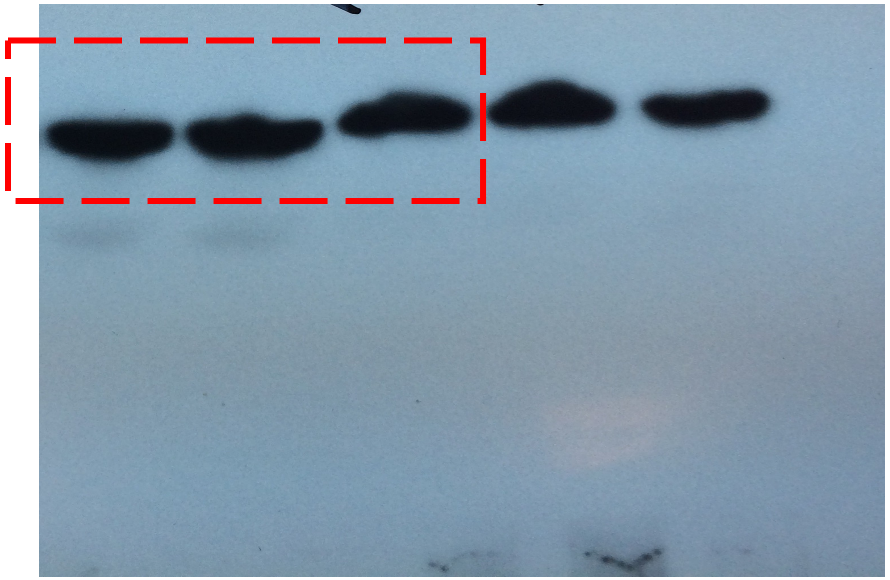

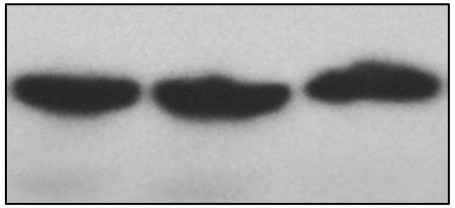

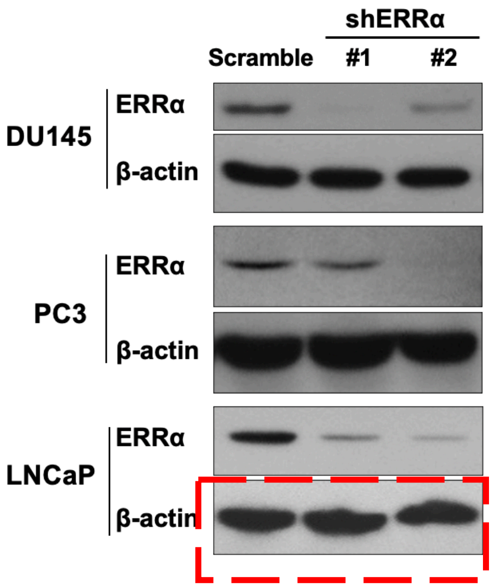
shERRα
Scramble
#1
#2
DU145
ERRα
β-actin
PC3
ERRα
β-actin
LNCaP
ERRα
β-actin

Fig. 2e

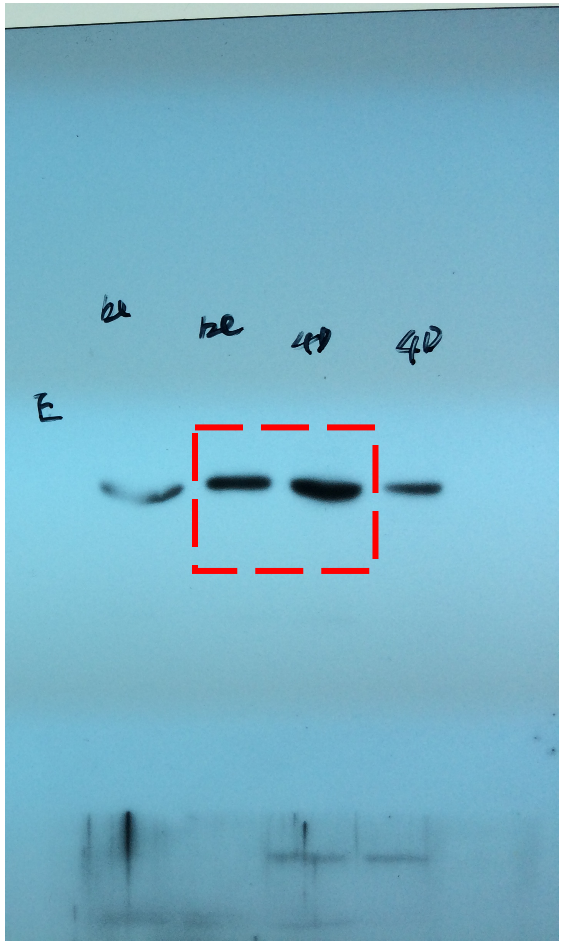

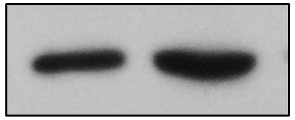

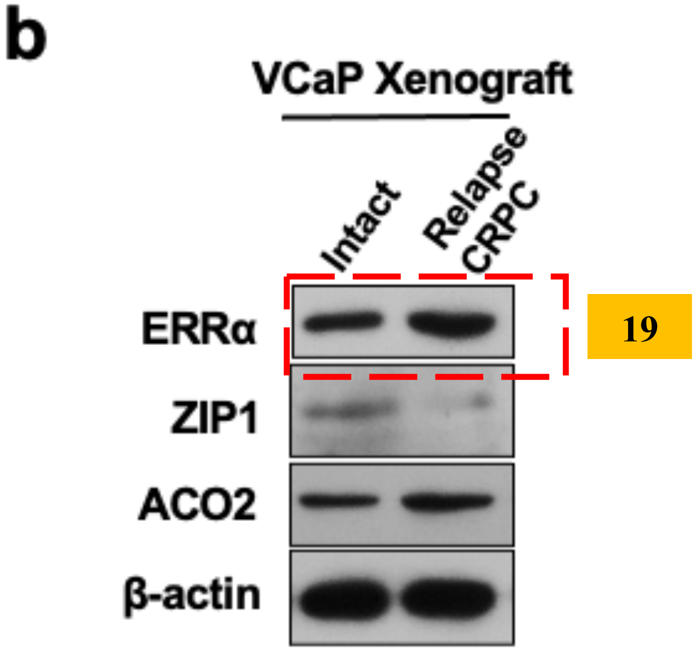
b
VCaP Xenograft
Intact
Relapse
CRPC
ERRα
ZIP1
ACO2
β-actin
19

Fig. 5b

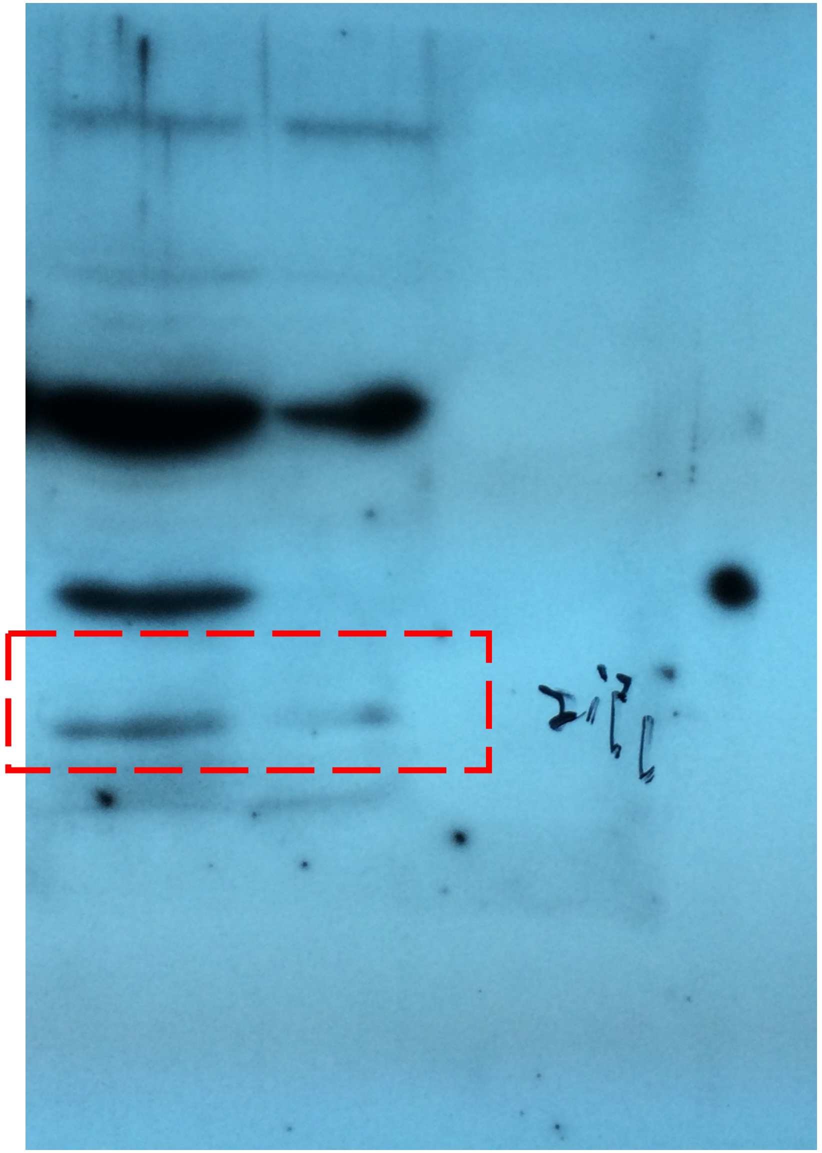

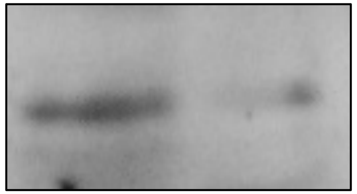

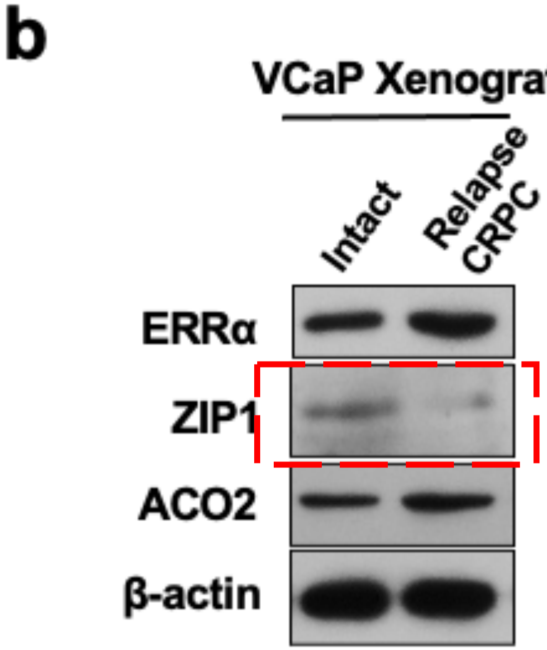

20

Fig. 5b

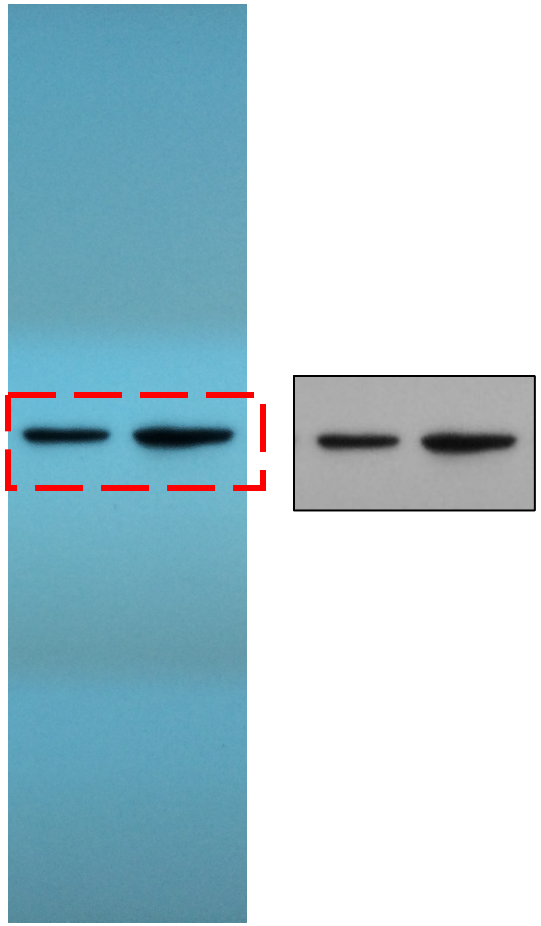

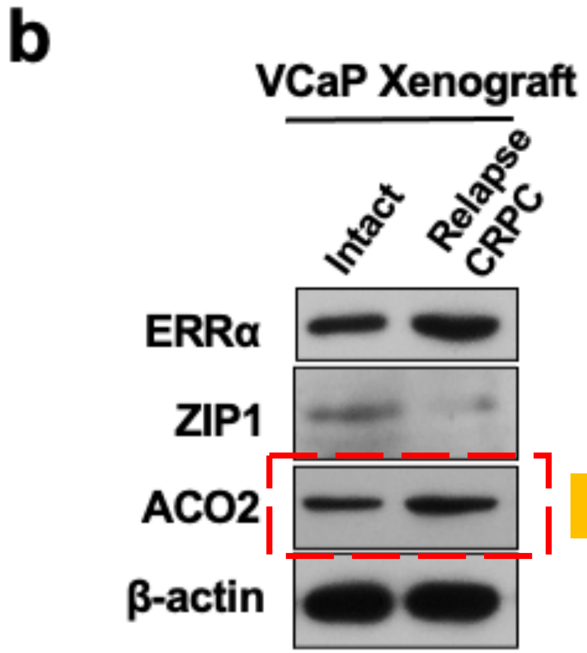

21

Fig. 5b

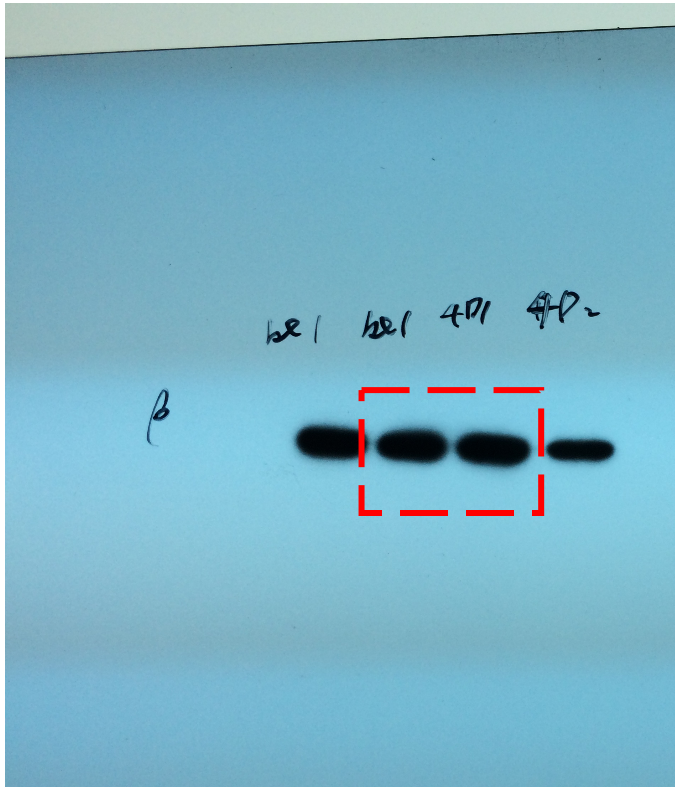

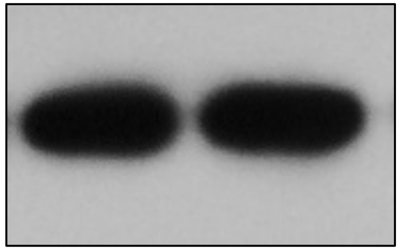

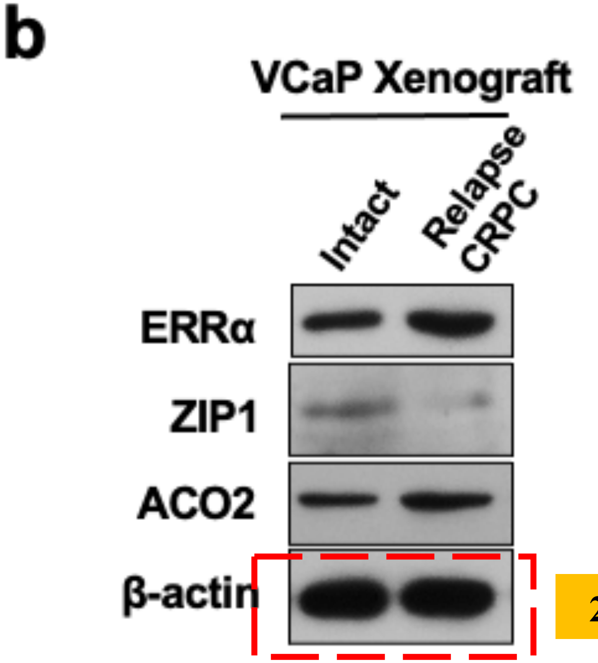

22

Fig. 5b

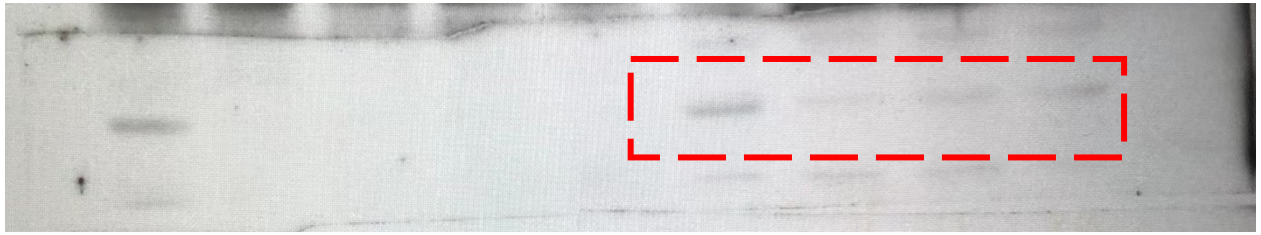

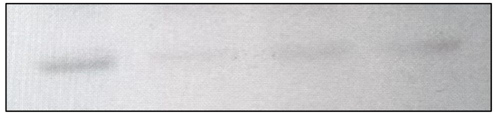

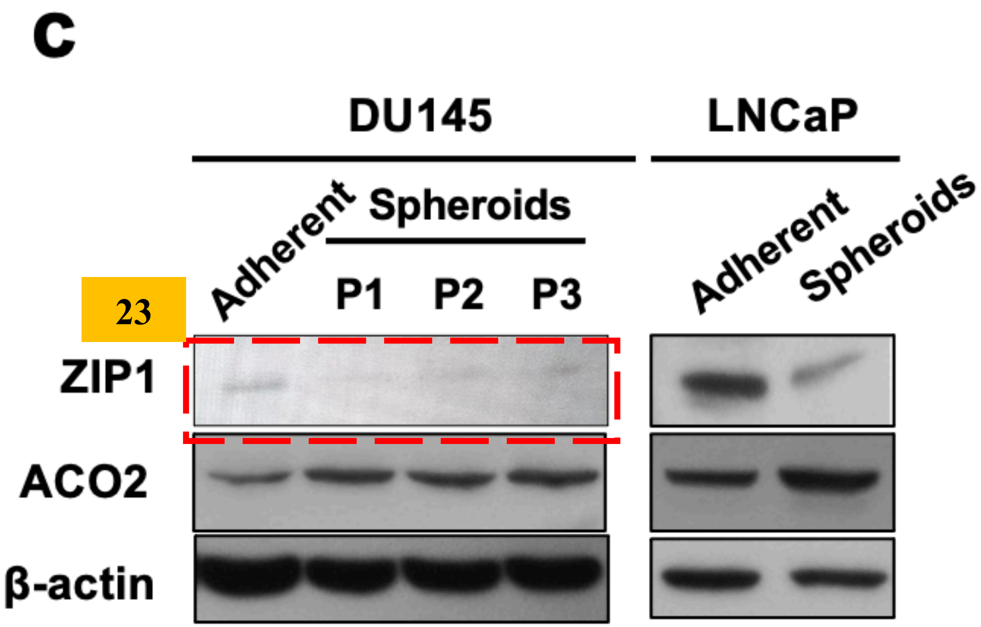
c
DU145
LNCaP
Adherent
Spheroids
P1
P2
P3
Adherent
Spheroids
23
ZIP1
ACO2
β-actin

Fig. 5c

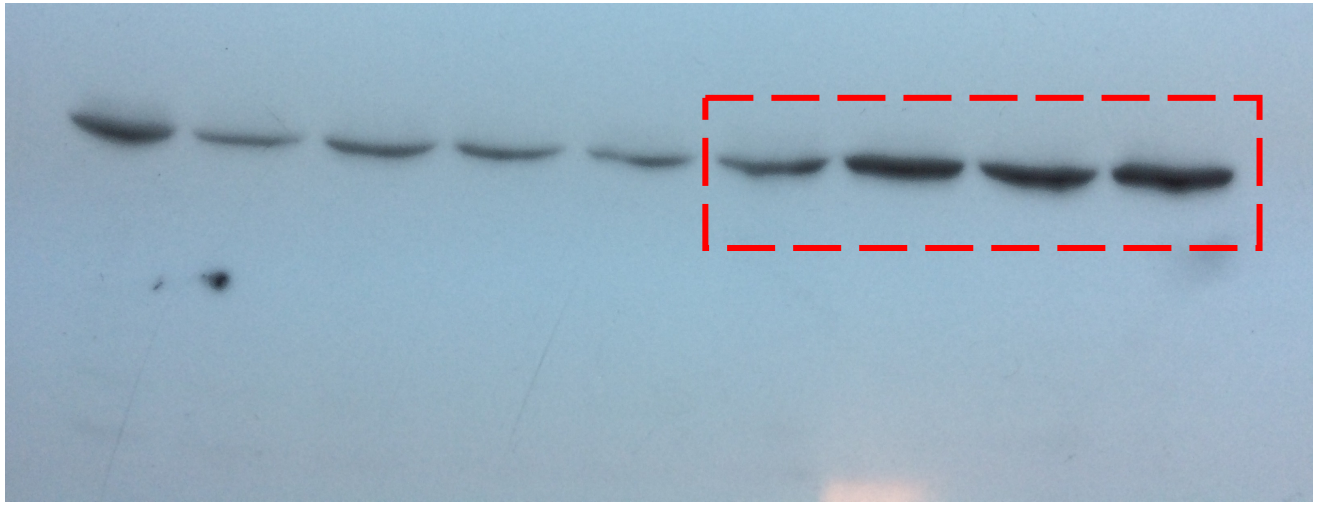

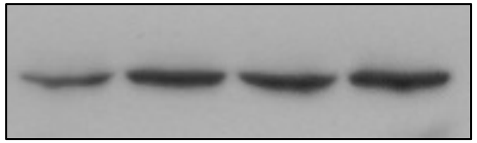

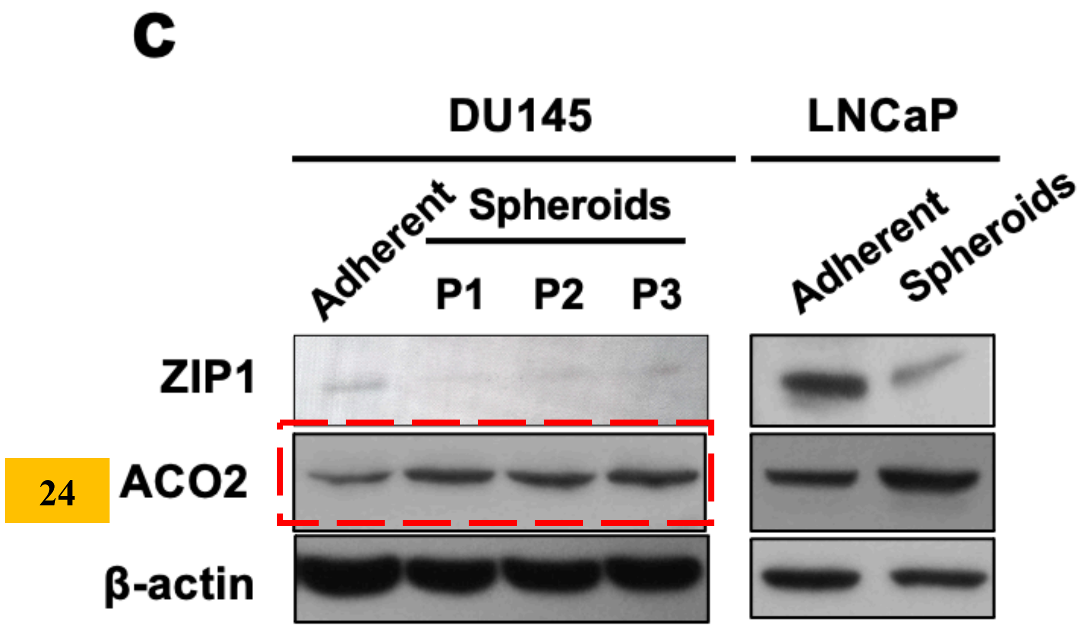
c
DU145
LNCaP
Adherent
Spheroids
P1
P2
P3
Adherent
Spheroids
ZIP1
24
ACO2
β-actin

Fig. 5c

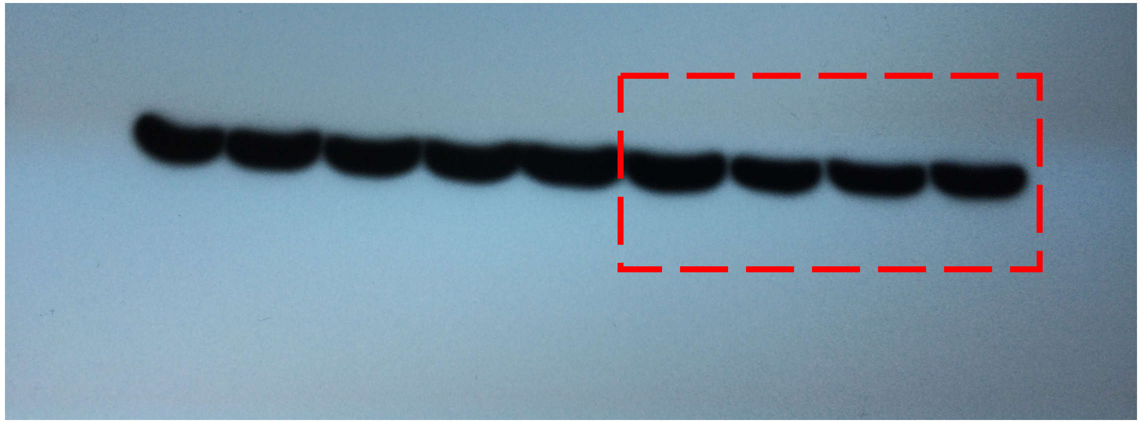

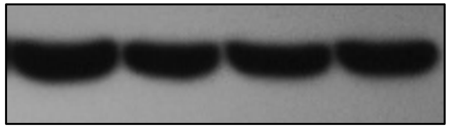

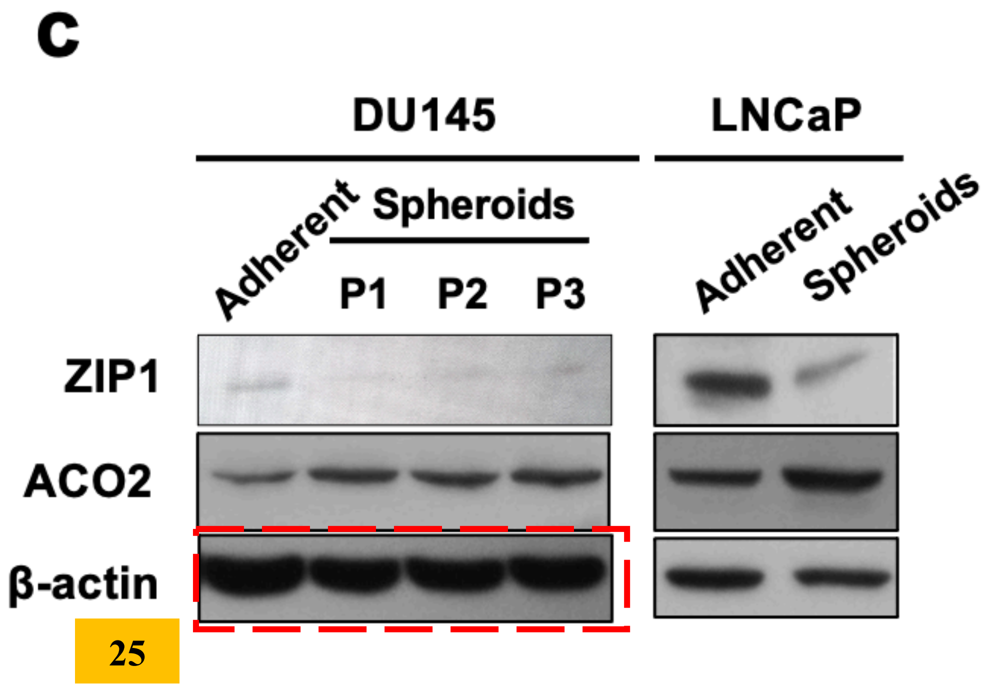
c
DU145
LNCaP
Adherent
Spheroids
P1
P2
P3
Adherent
Spheroids
ZIP1
ACO2
β-actin

Fig. 5c

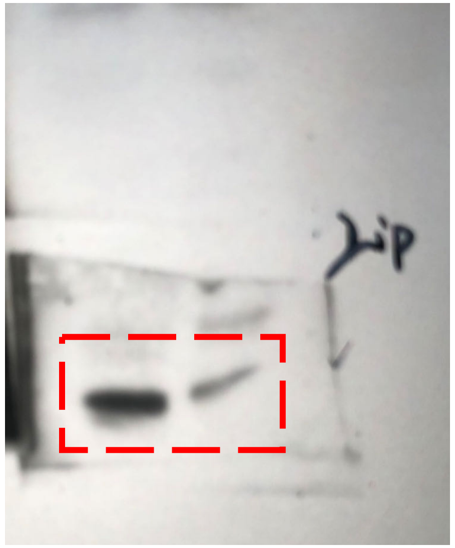
Zip

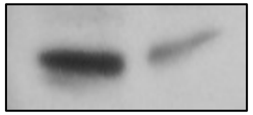

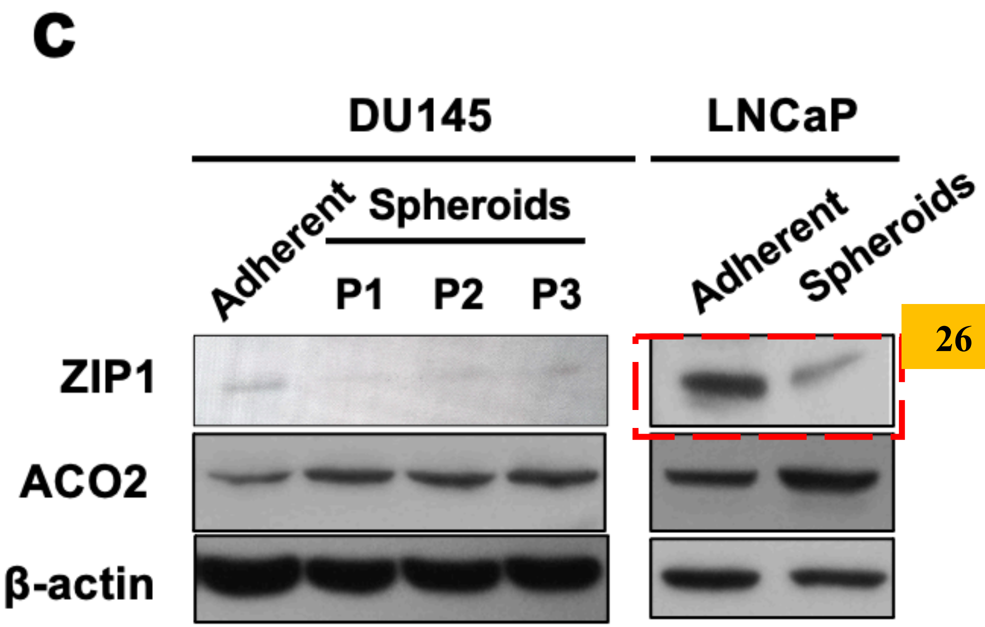
c
DU145
LNCaP
Adherent
Spheroids
P1
P2
P3
Adherent
Spheroids
ZIP1
ACO2
β-actin

26

Fig. 5c

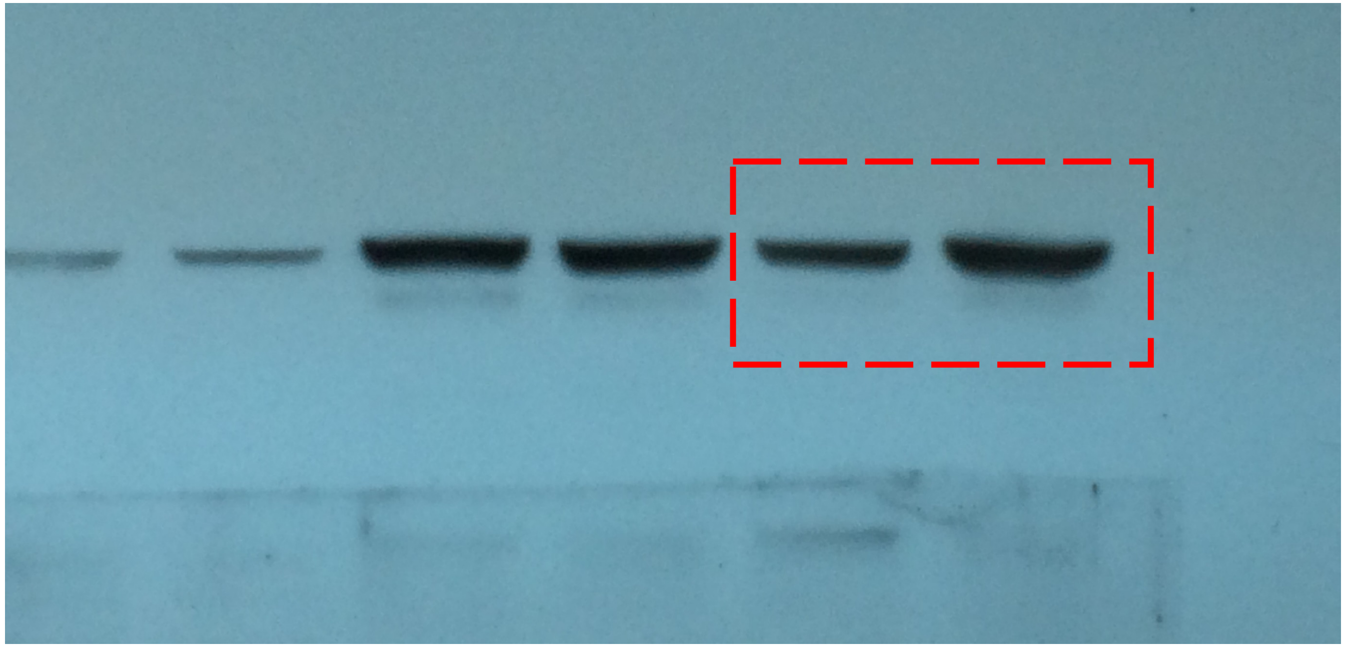

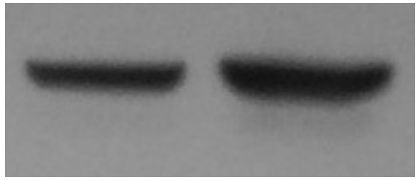

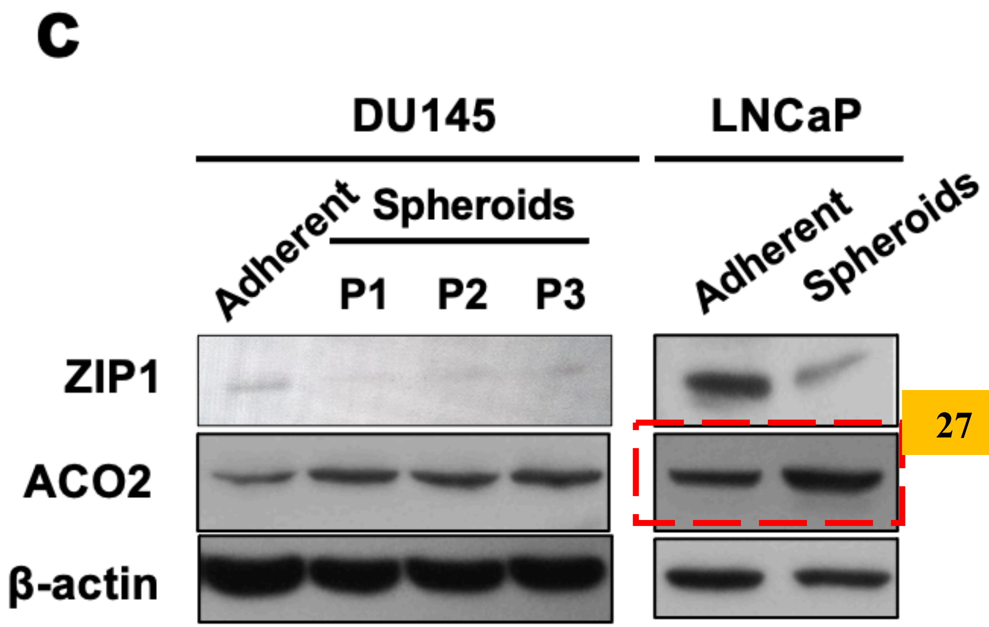
c
DU145
LNCaP
Adherent
Spheroids
P1
P2
P3
Adherent
Spheroids
ZIP1
ACO2
β-actin

Fig. 5c

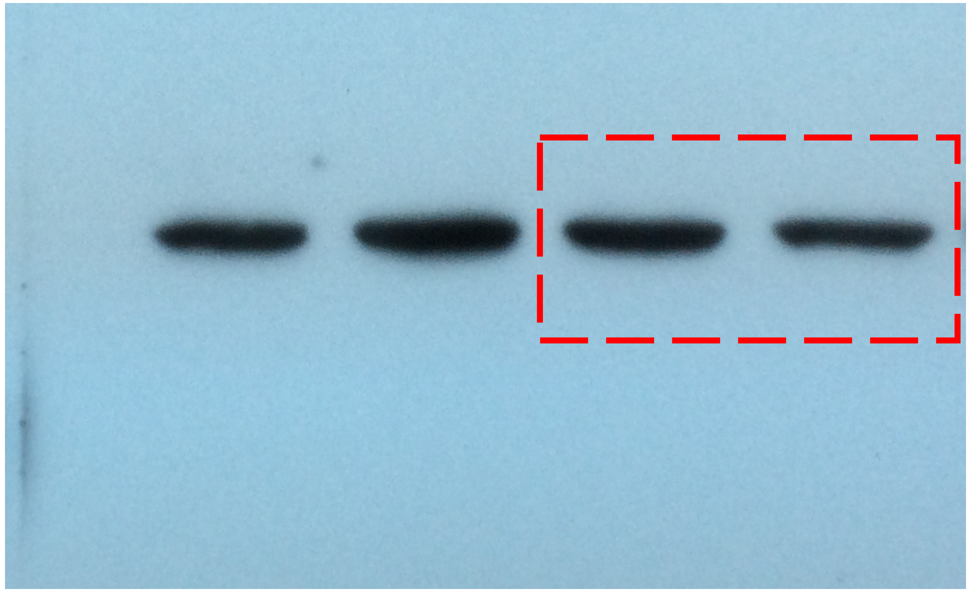

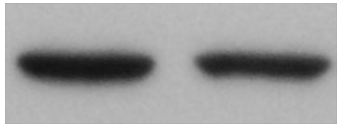

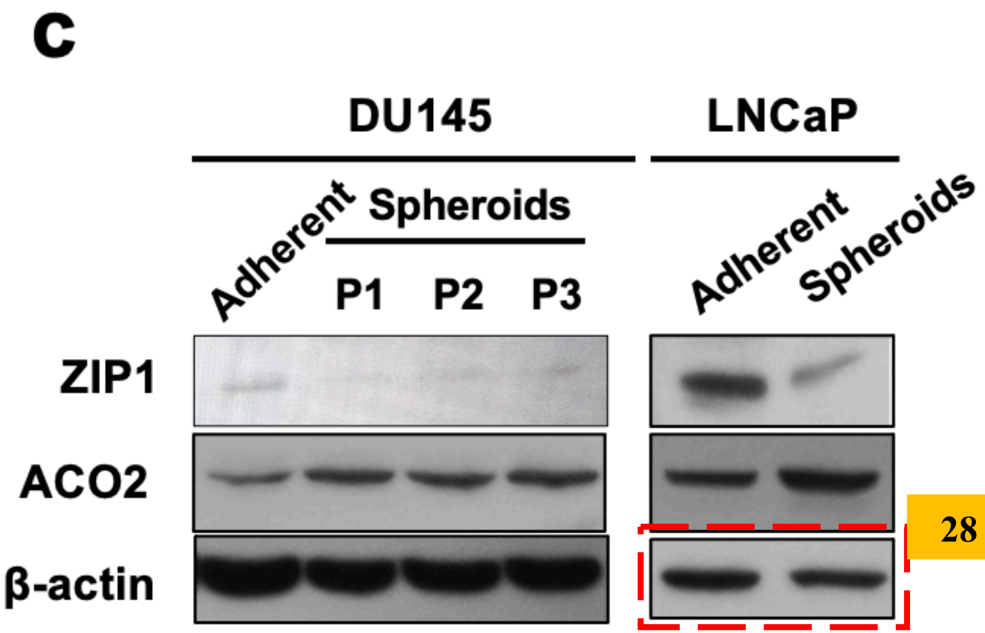
c
DU145
LNCaP
Adherent
Spheroids
P1
P2
P3
Adherent
Spheroids
ZIP1
ACO2
β-actin

Fig. 5c

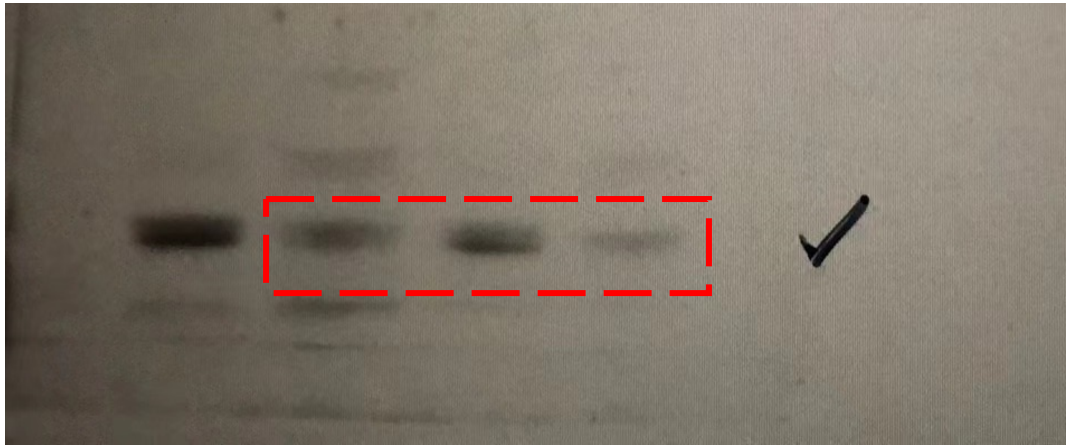

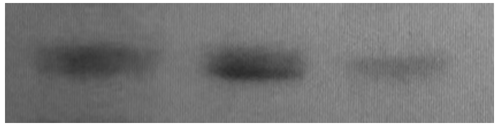

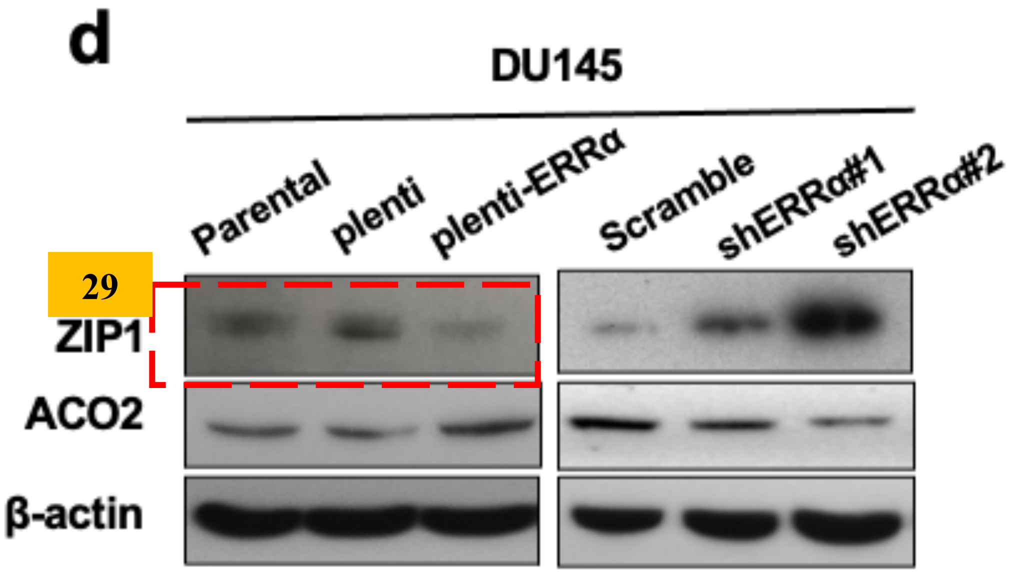
d
DU145
Parental
plenti
plenti-ERRα
Scramble
shERRα#1
shERRα#2
29
ZIP1
ACO2
β-actin

Fig. 5d

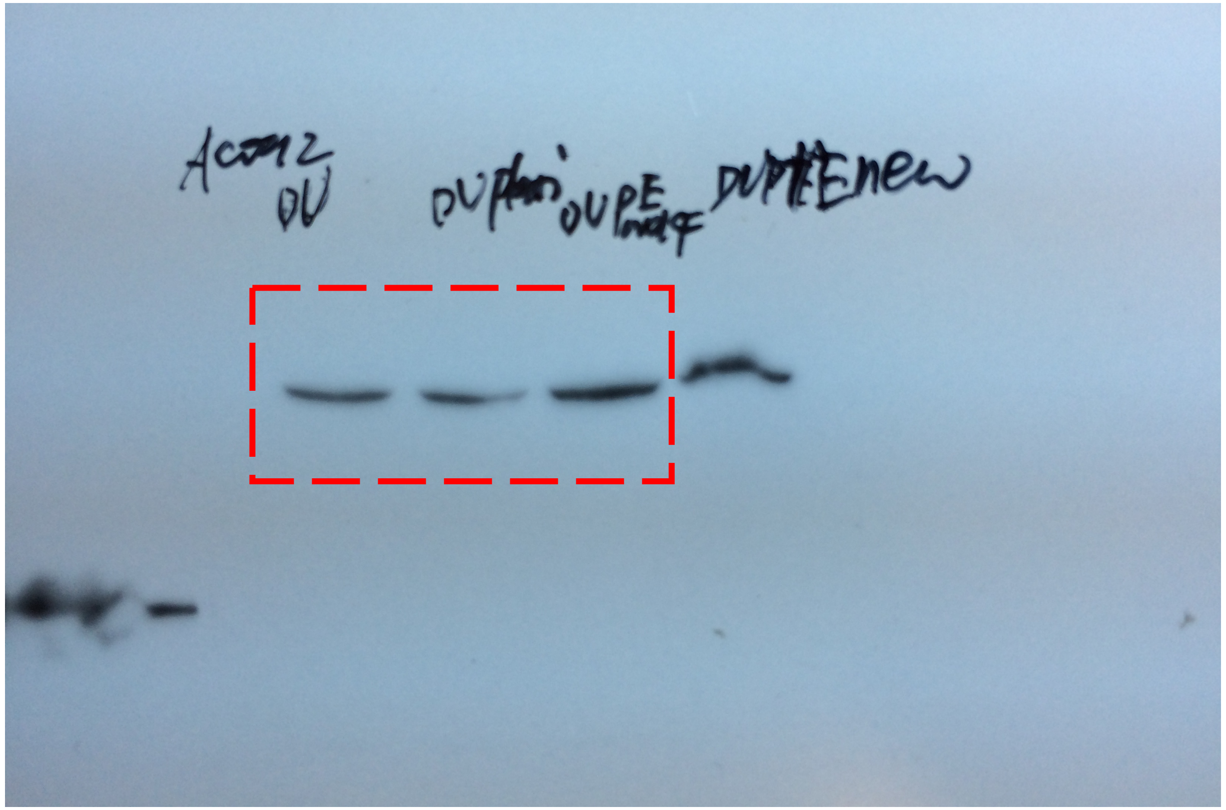

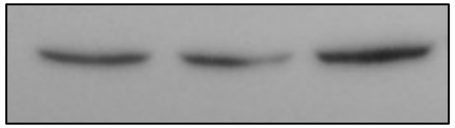

30

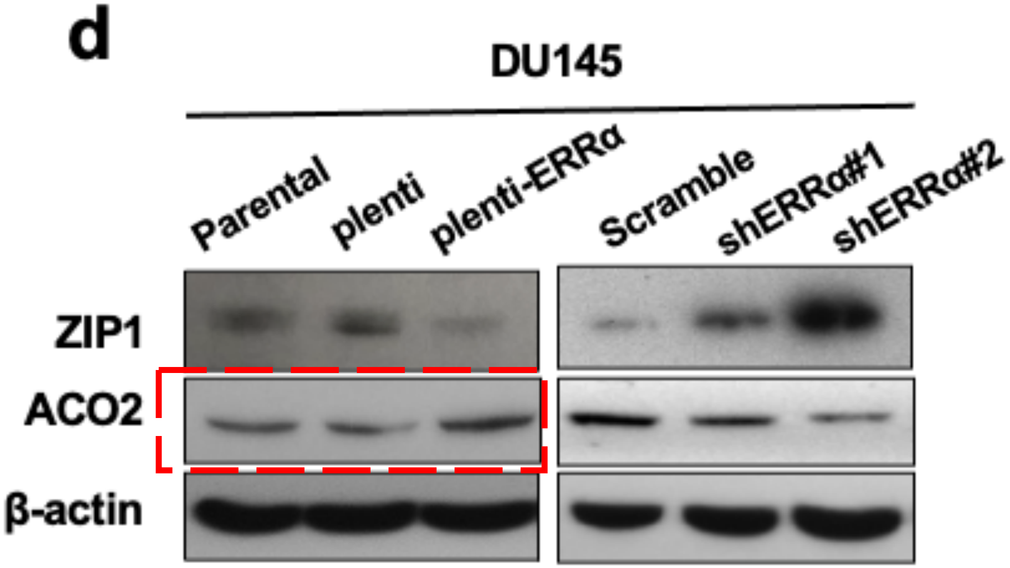
d
DU145
Parental
plenti
plenti-ERRα
Scramble
shERRα#1
shERRα#2
ZIP1
ACO2
β-actin

Fig. 5d

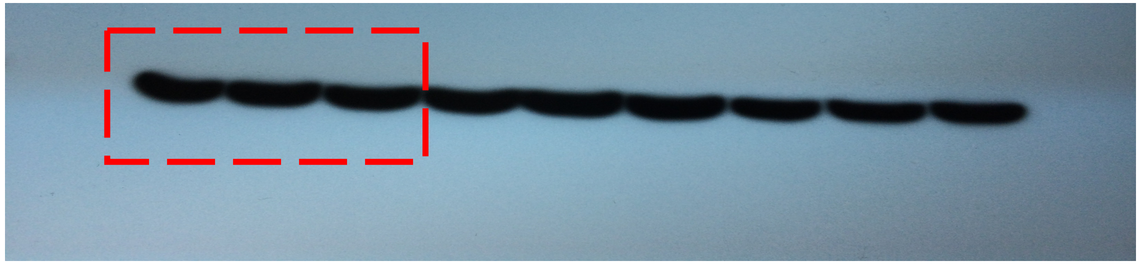

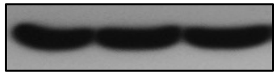

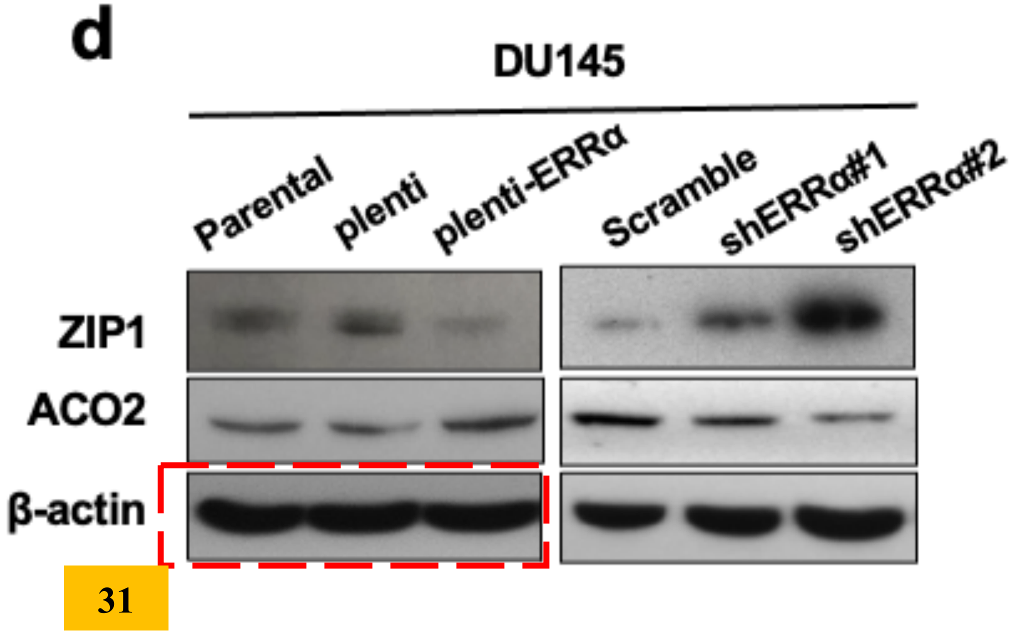
d
DU145
Parental
plenti
plenti-ERRα
Scramble
shERRα#1
shERRα#2
ZIP1
ACO2
β-actin

31

Fig. 5d

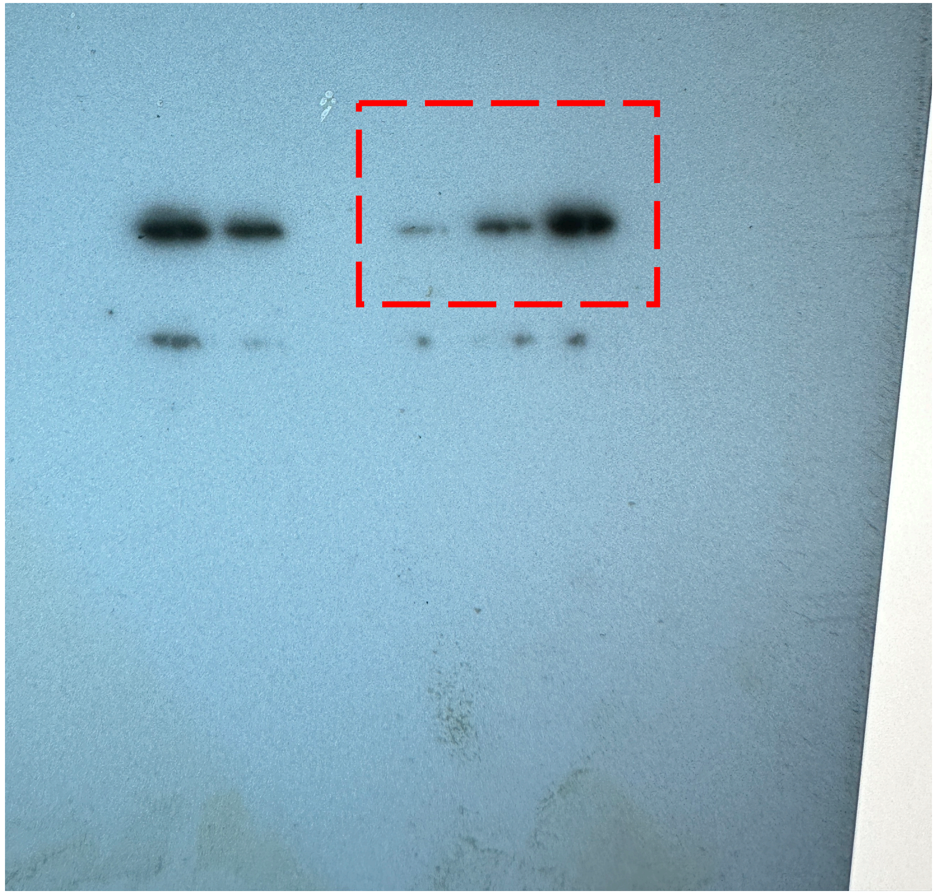

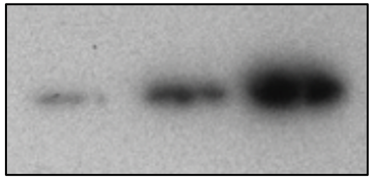

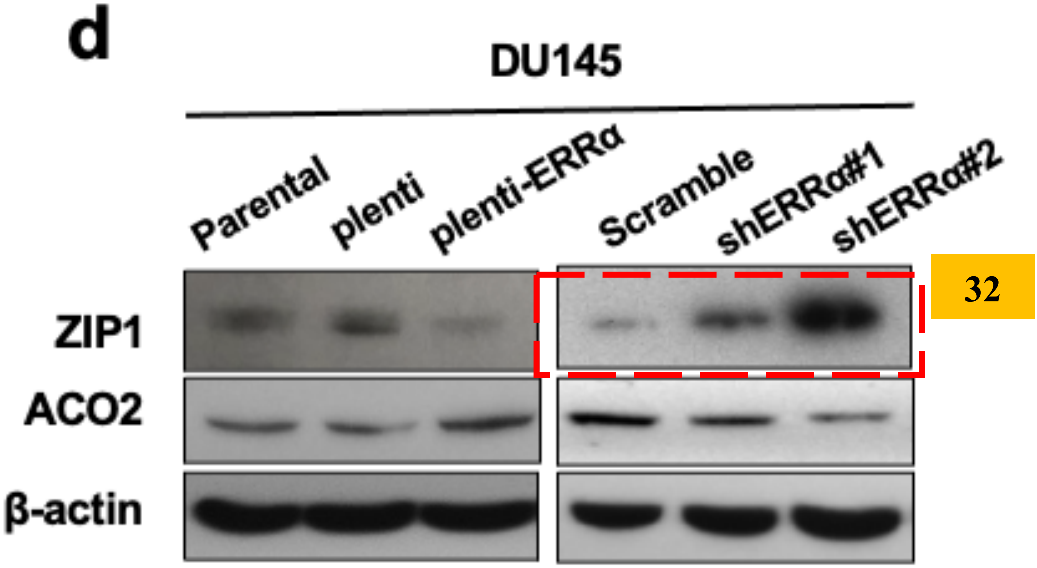
d
DU145
Parental
plenti
plenti-ERRα
Scramble
shERRα#1
shERRα#2
ZIP1
ACO2
β-actin
32

Fig. 5d

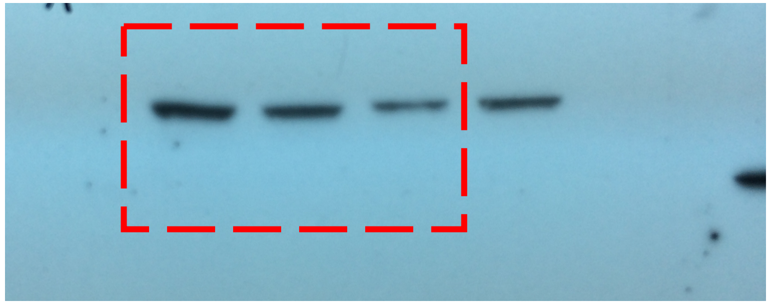

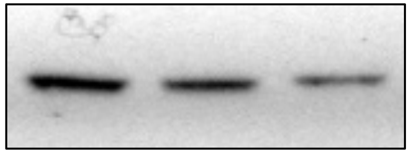

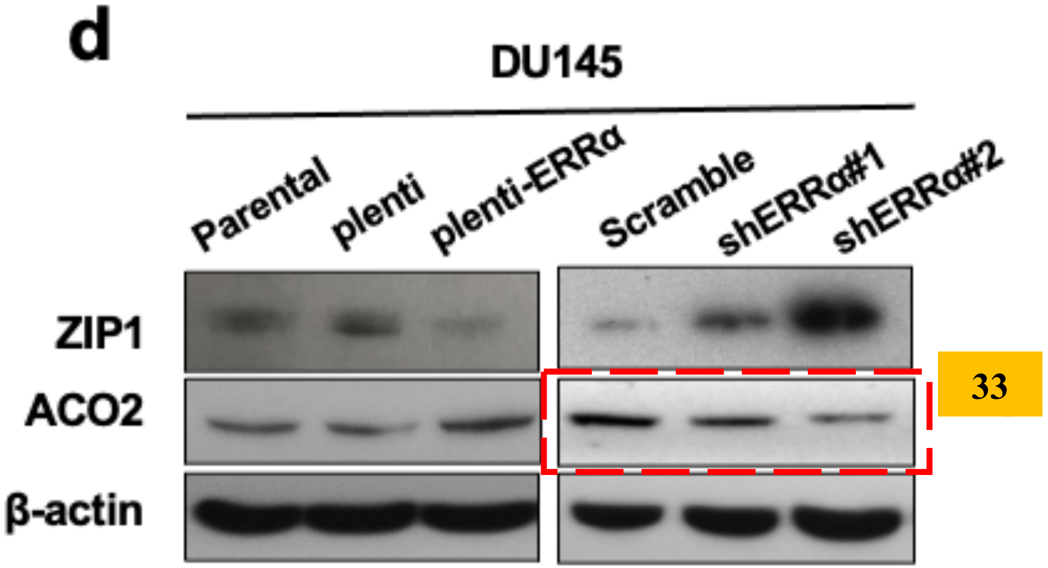
d
DU145
Parental
plenti
plenti-ERRα
Scramble
shERRα#1
shERRα#2
ZIP1
ACO2
β-actin

33

Fig. 5d

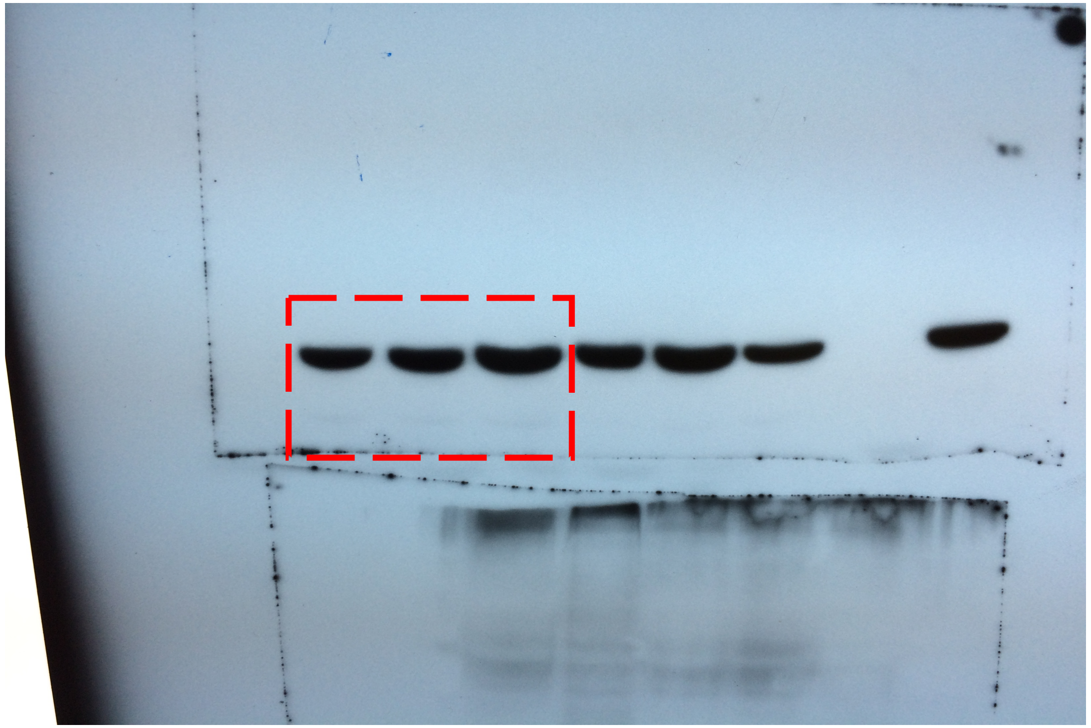

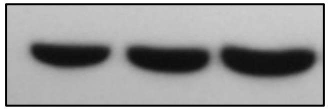

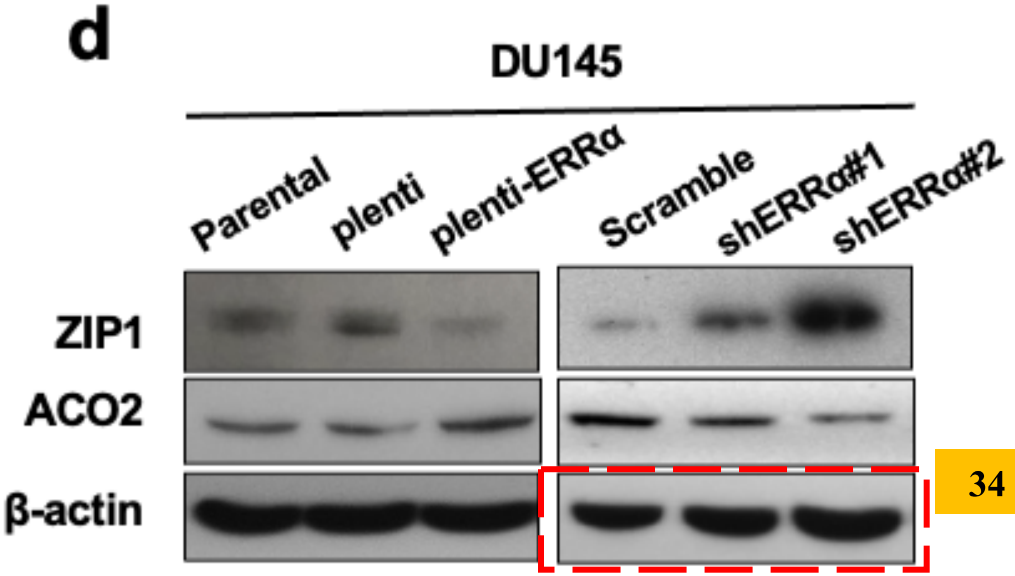
d
DU145
Parental
plenti
plenti-ERRα
Scramble
shERRα#1
shERRα#2
ZIP1
ACO2
β-actin

34

Fig. 5d

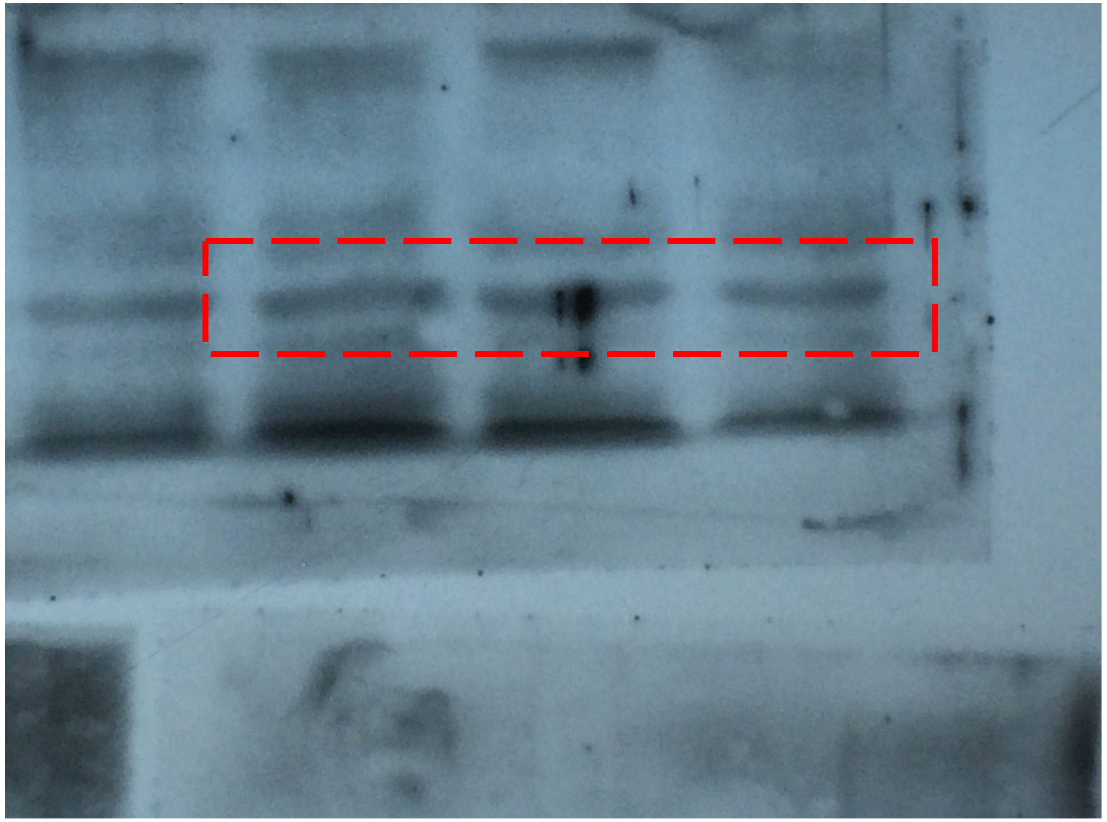

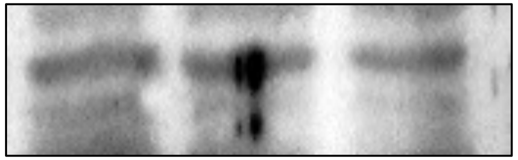

35

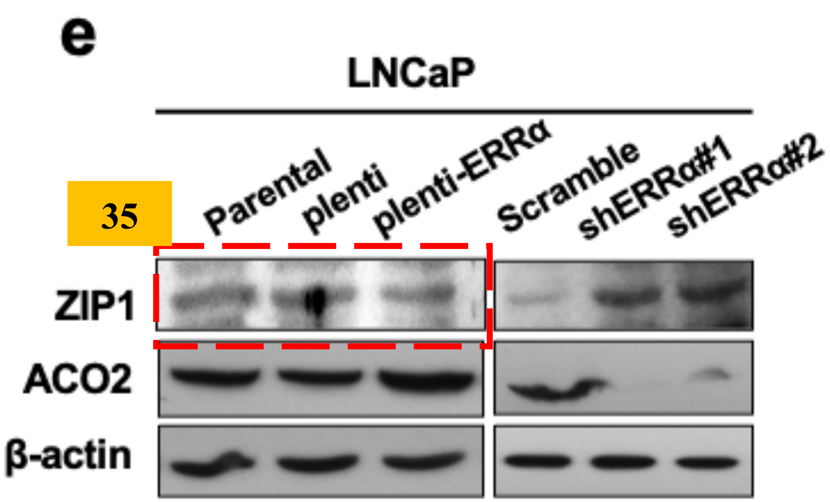
e
LNCaP
Parental
plenti
plenti-ERRα
Scramble
shERRα#1
shERRα#2
ZIP1
ACO2
β-actin

Fig. 5e

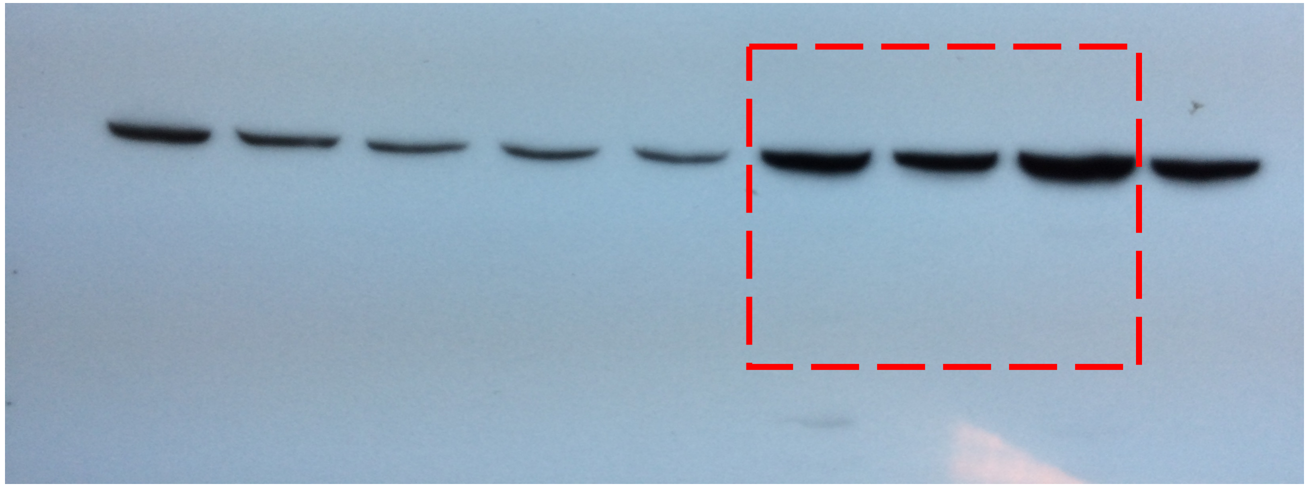

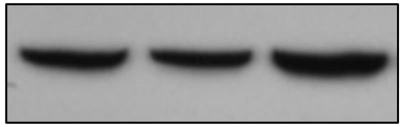

36

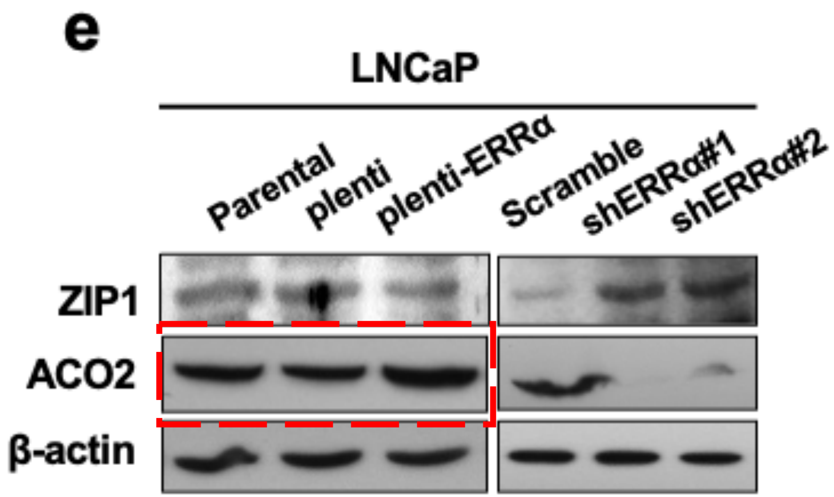
e
LNCaP
Parental
plenti
plenti-ERRα
Scramble
shERRα#1
shERRα#2
ZIP1
ACO2
β-actin

Fig. 5e

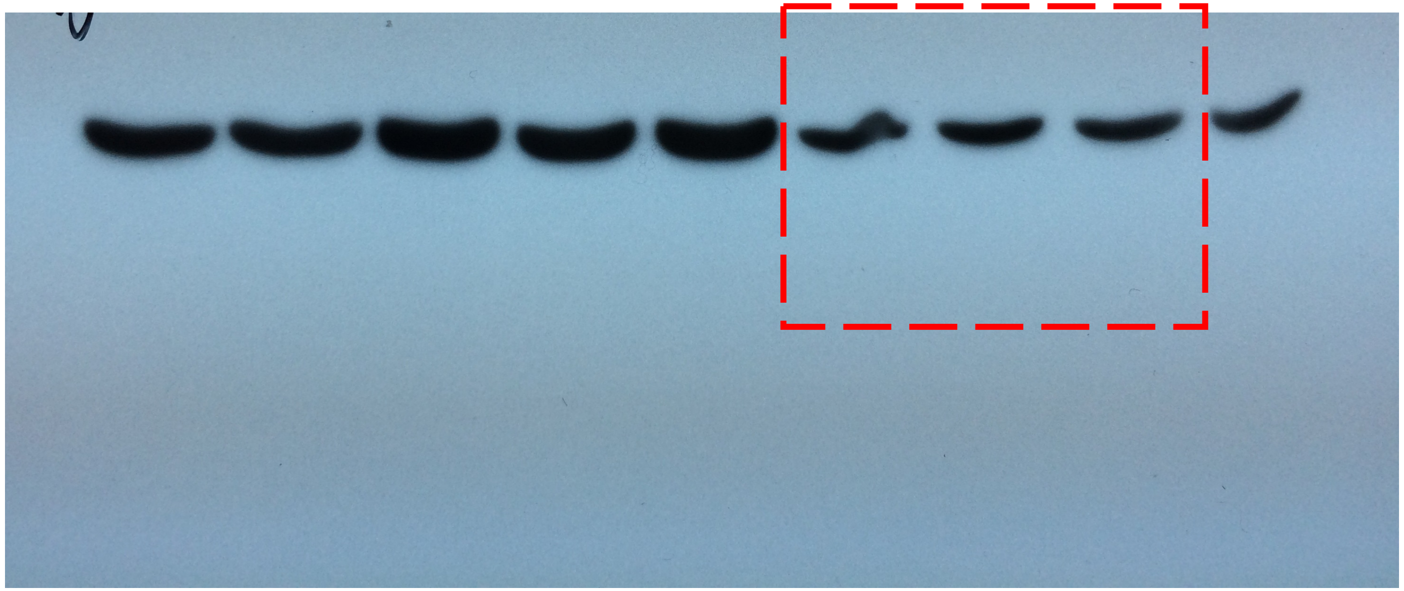

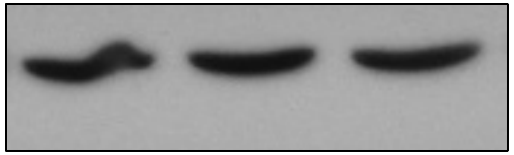

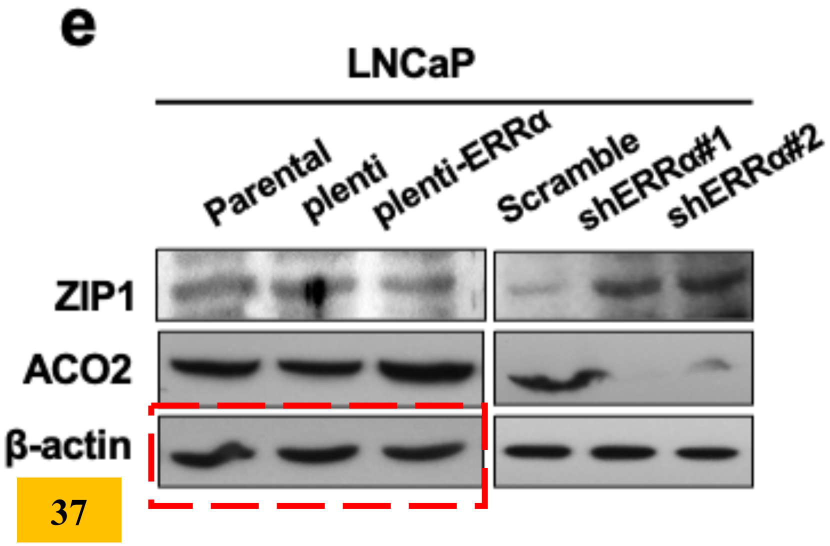
e
LNCaP
Parental
plenti
plenti-ERRα
Scramble
shERRα#1
shERRα#2
ZIP1
ACO2
β-actin

Fig. 5e

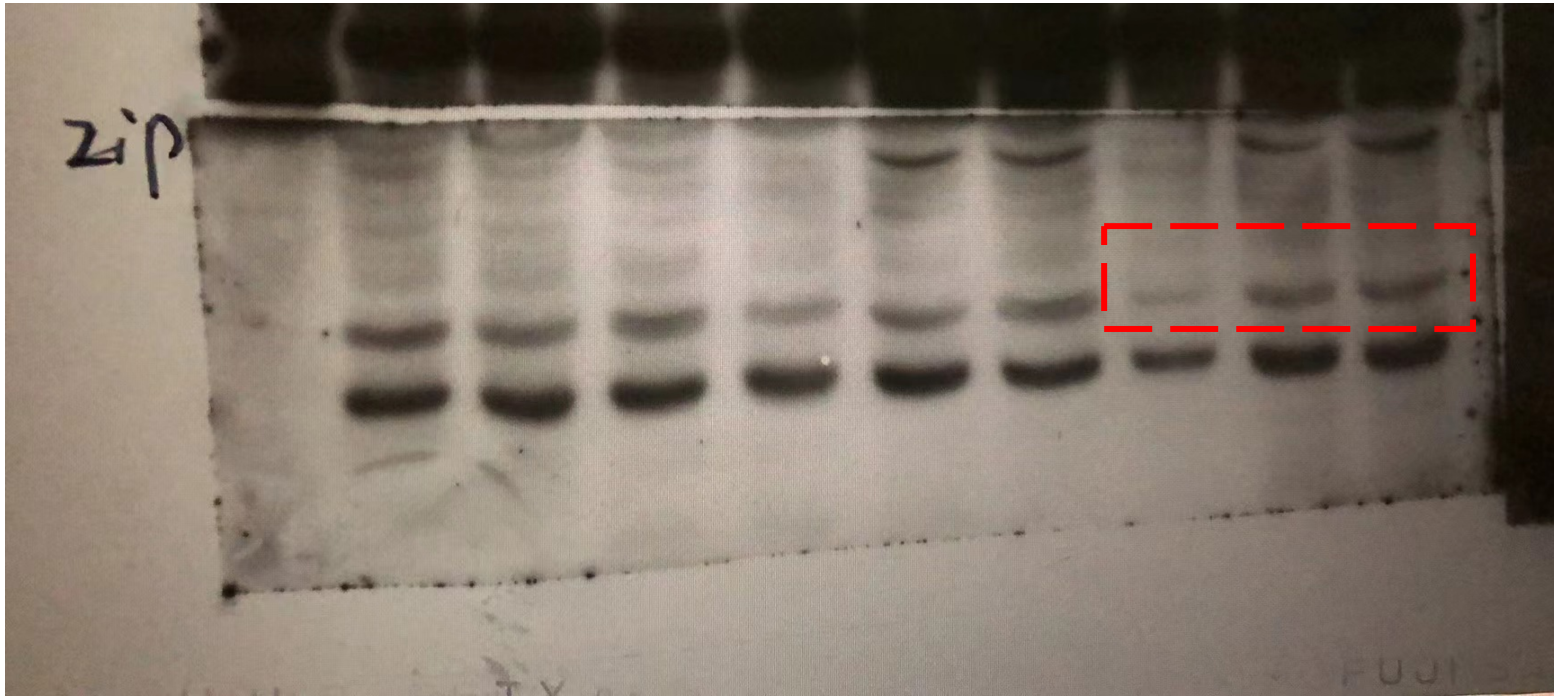
zip

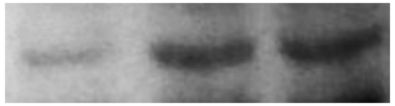

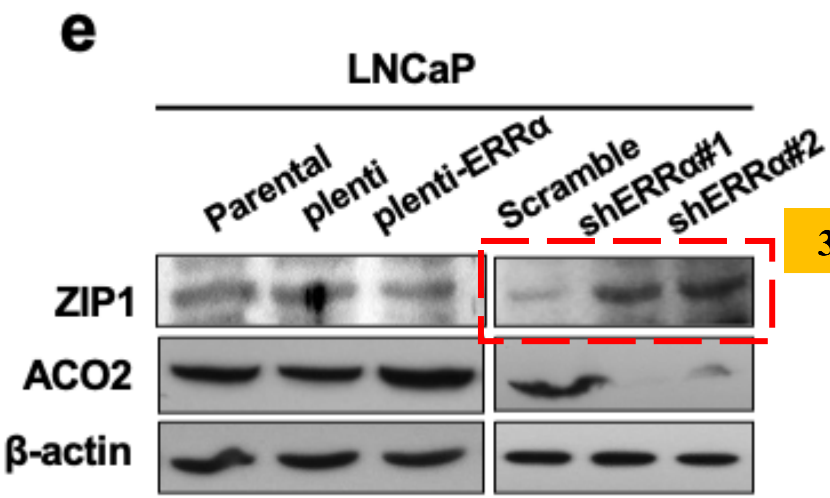
e
LNCaP
Parental
plenti
plenti-ERRα
Scramble
shERRα#1
shERRα#2
ZIP1
ACO2
β-actin

38

Fig. 5e

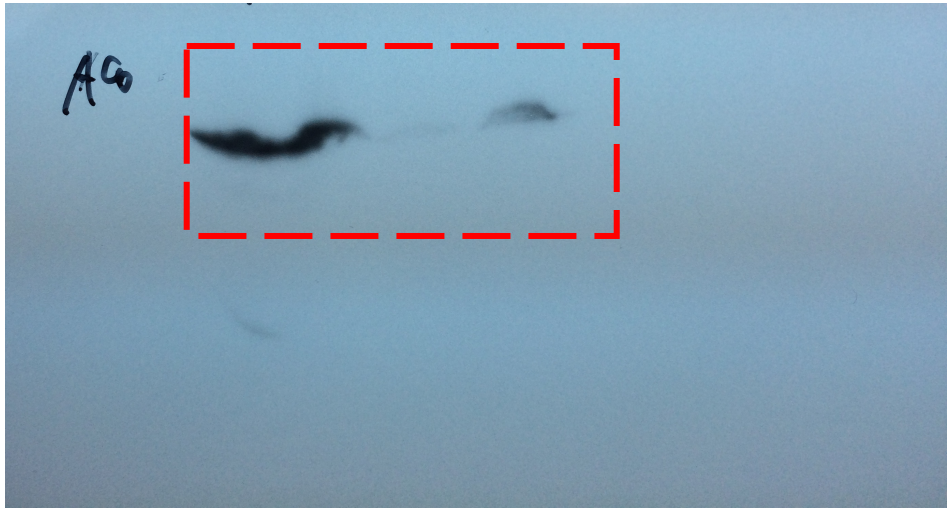

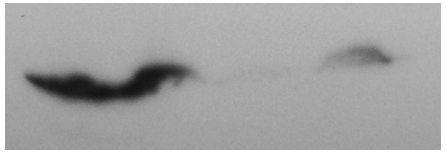

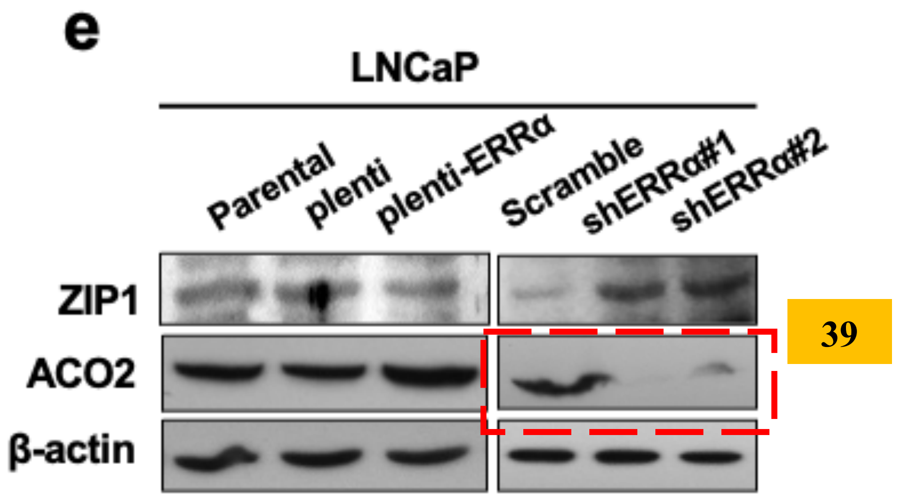
e
LNCaP
Parental
plenti
plenti-ERRα
Scramble
shERRα#1
shERRα#2
ZIP1
ACO2
β-actin
39

Fig. 5e

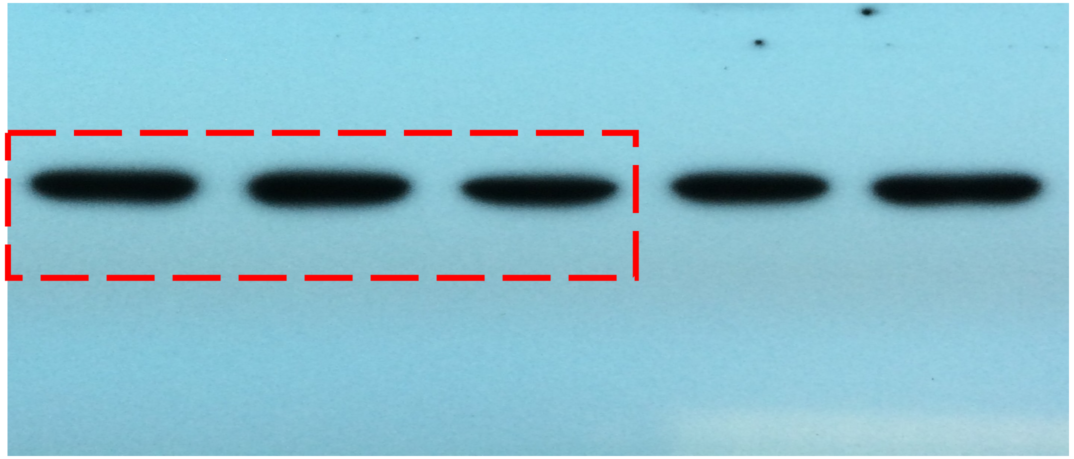

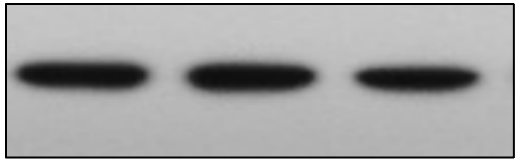

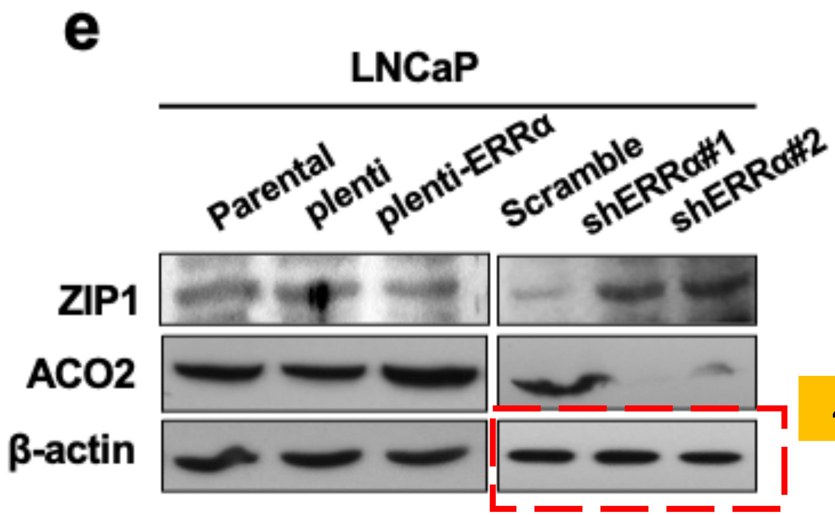
e
LNCaP
Parental
plenti
plenti-ERRα
Scramble
shERRα#1
shERRα#2
ZIP1
ACO2
β-actin

40

Fig. 5e

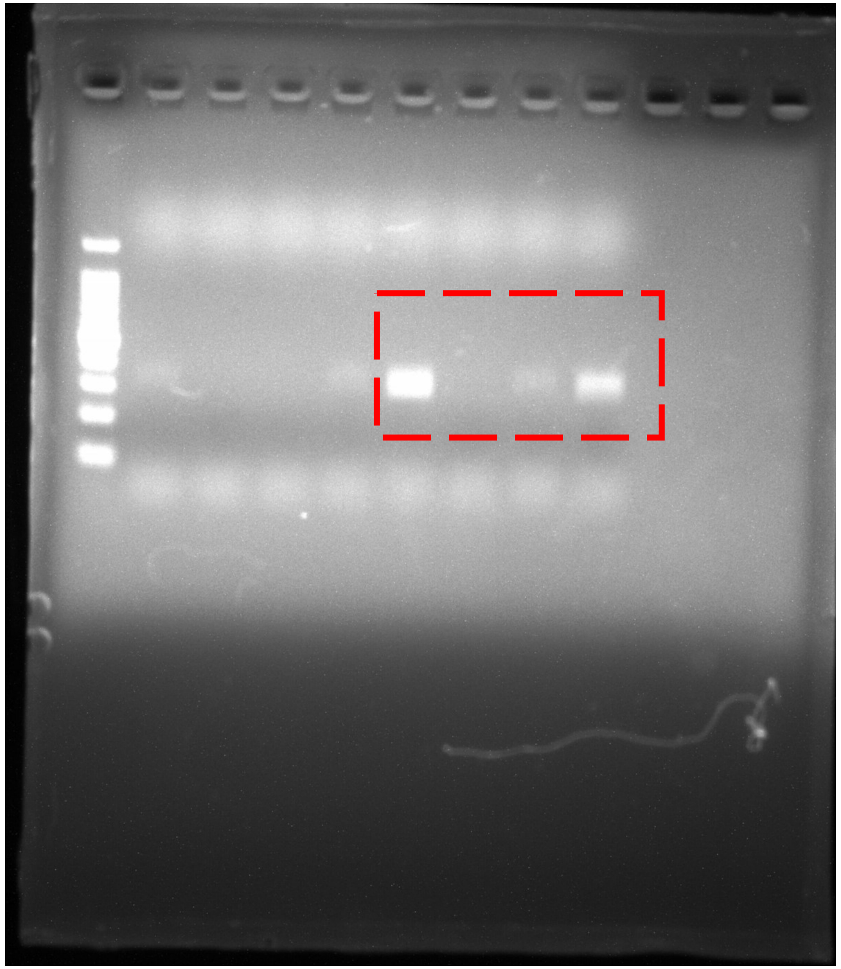

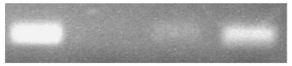

41

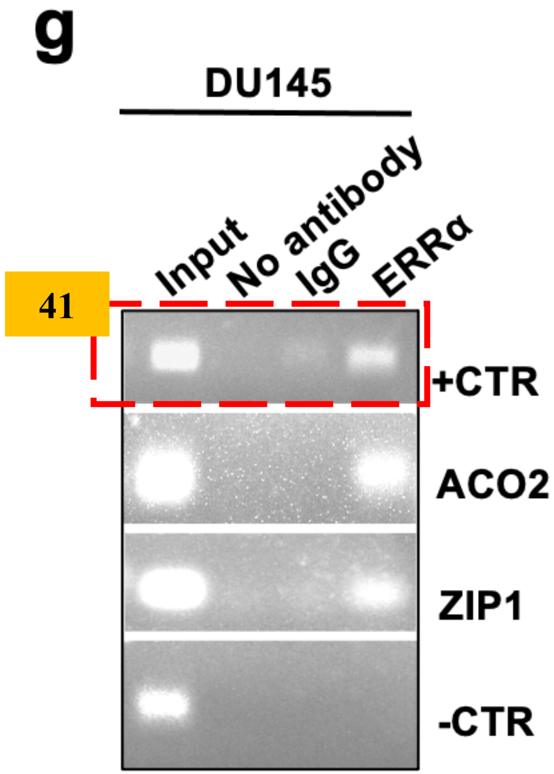

Fig. 5g

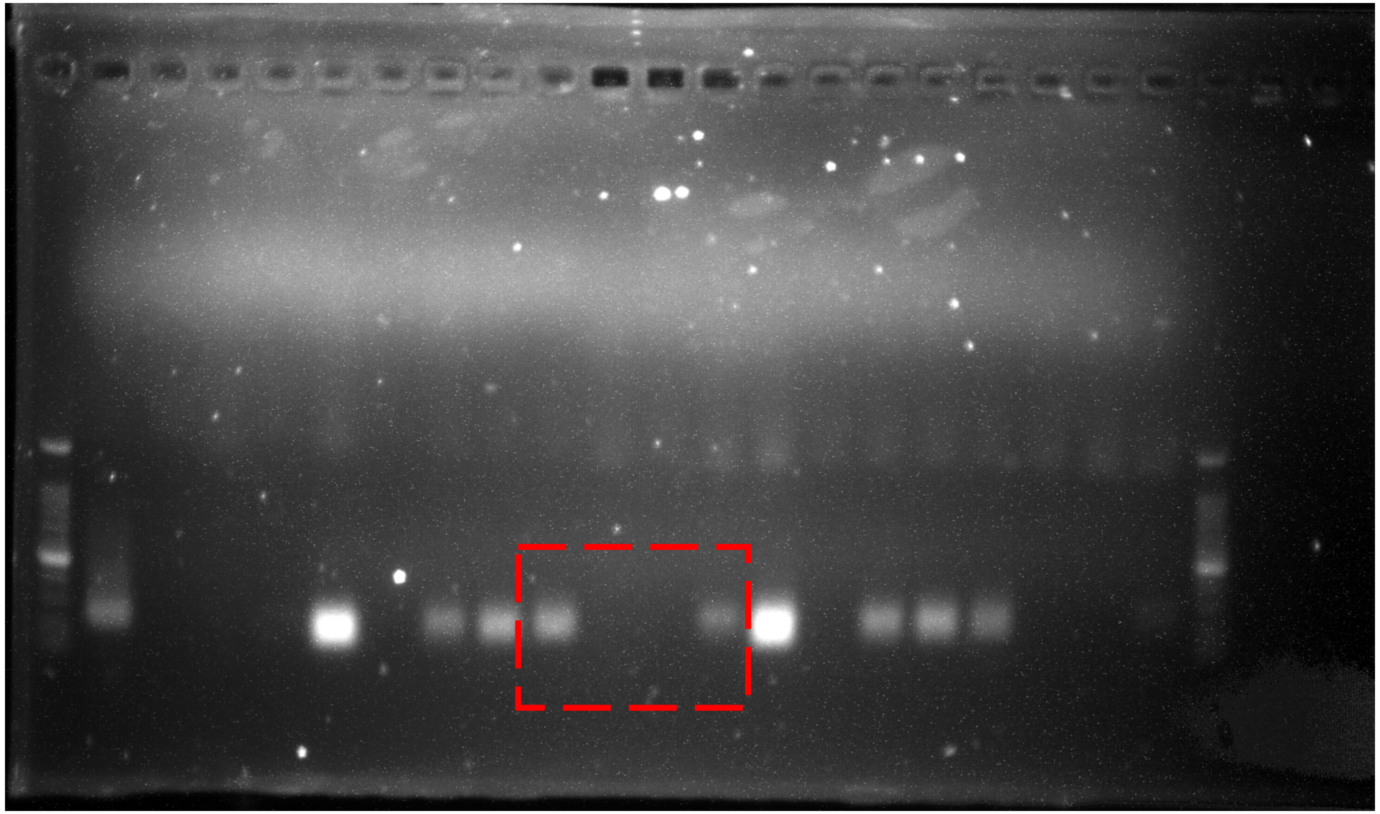

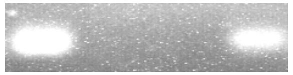

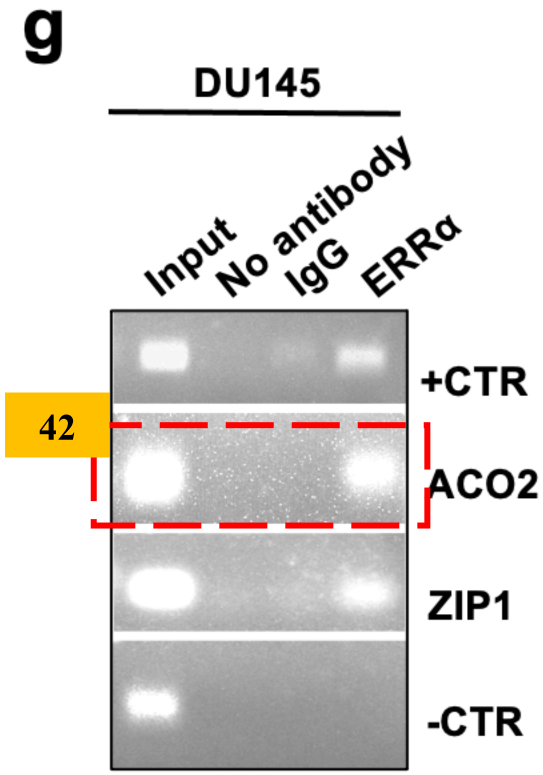

Fig. 5g

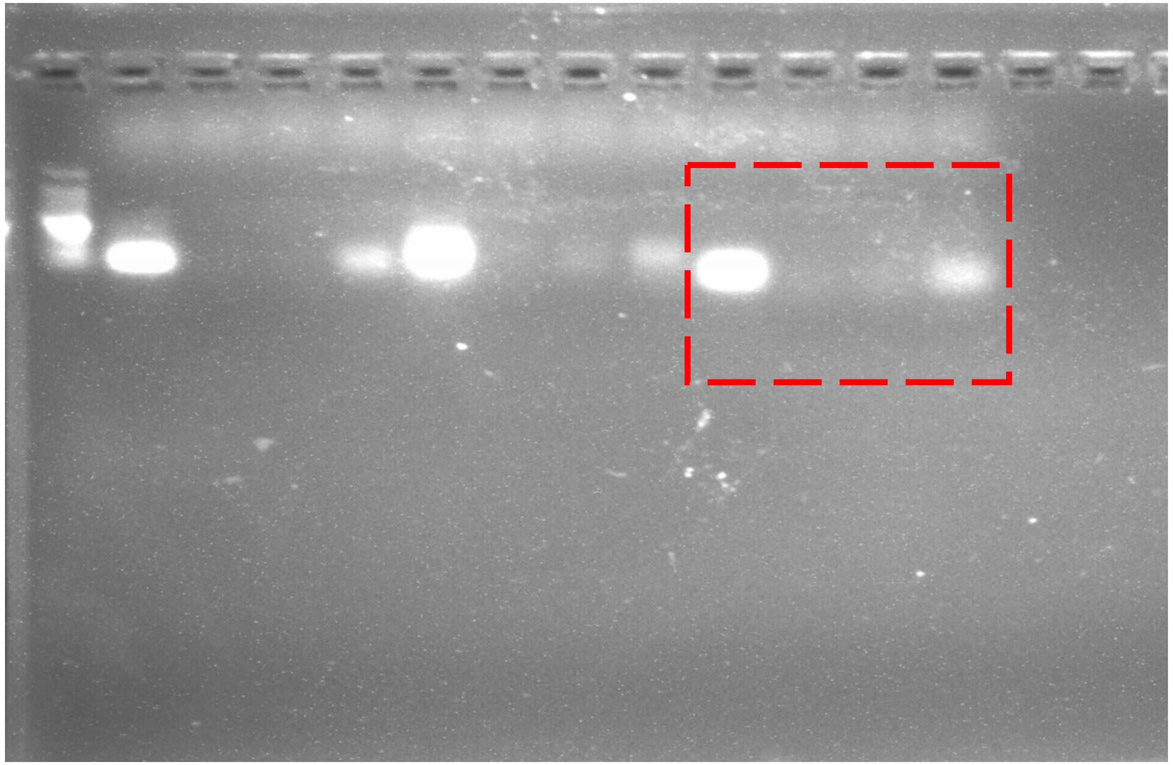

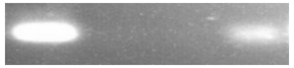

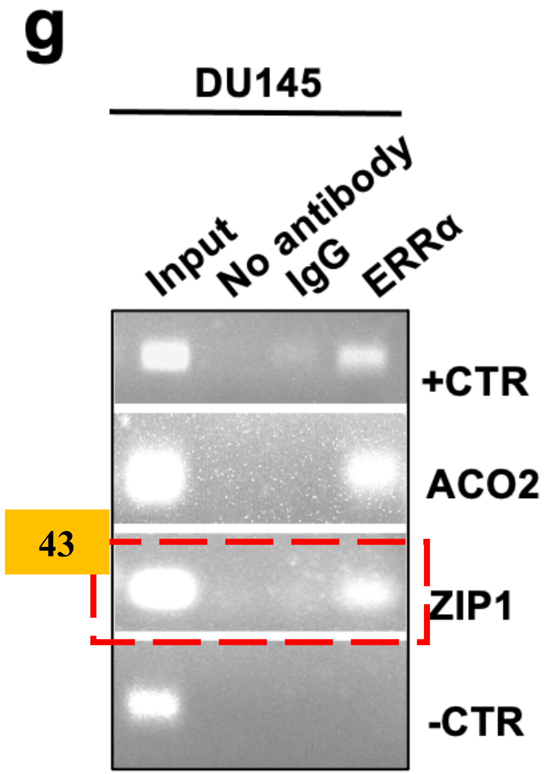

Fig. 5g

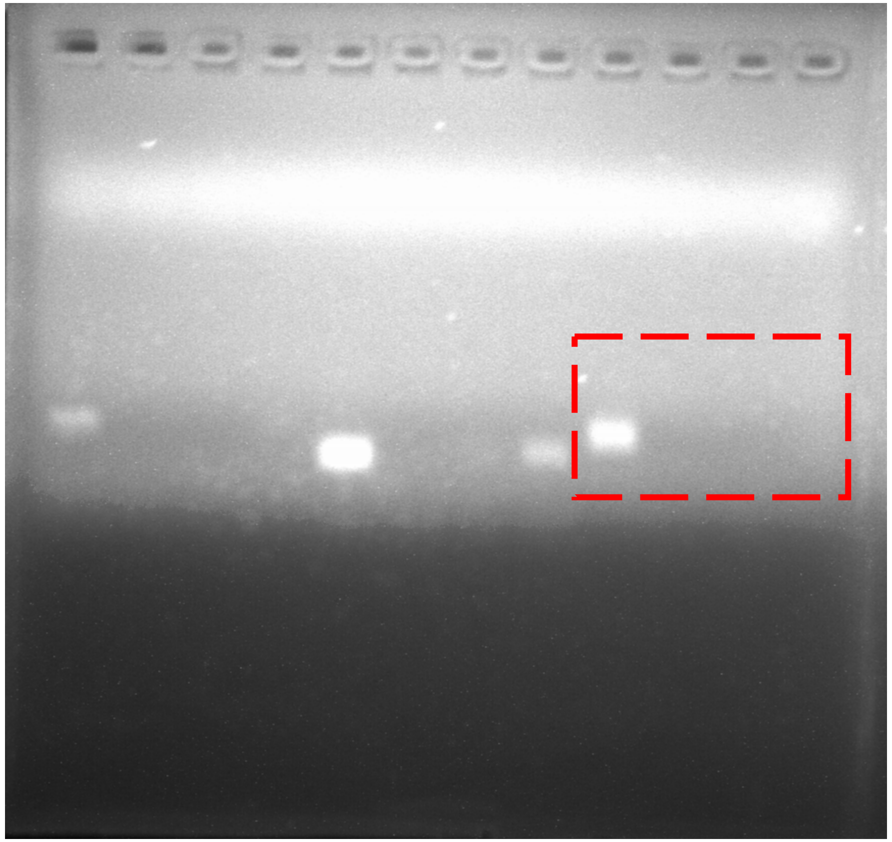

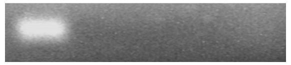

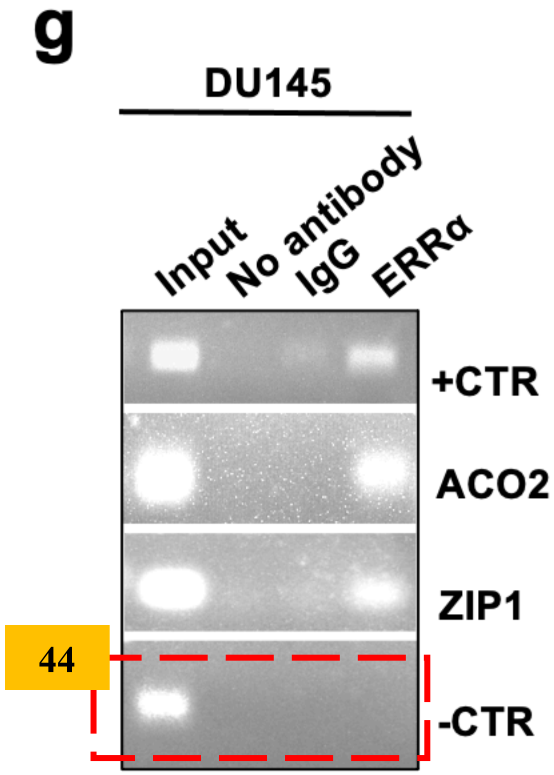

Fig. 5g

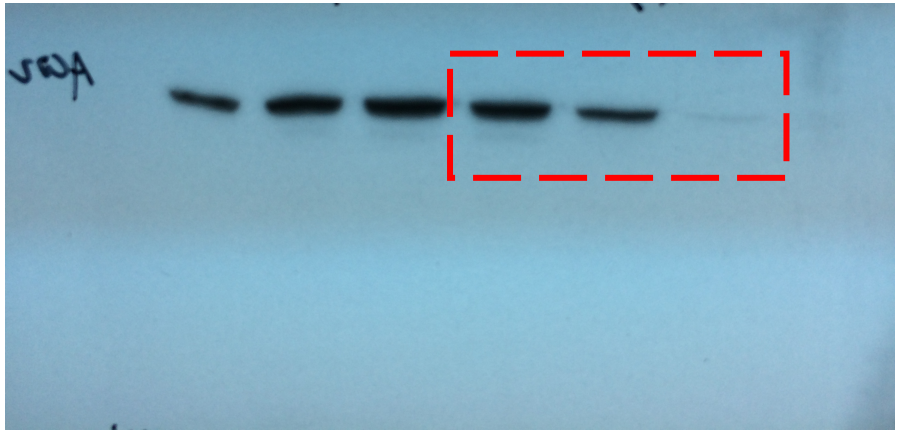

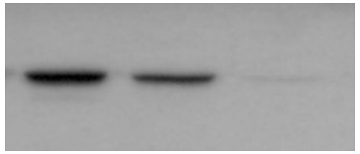

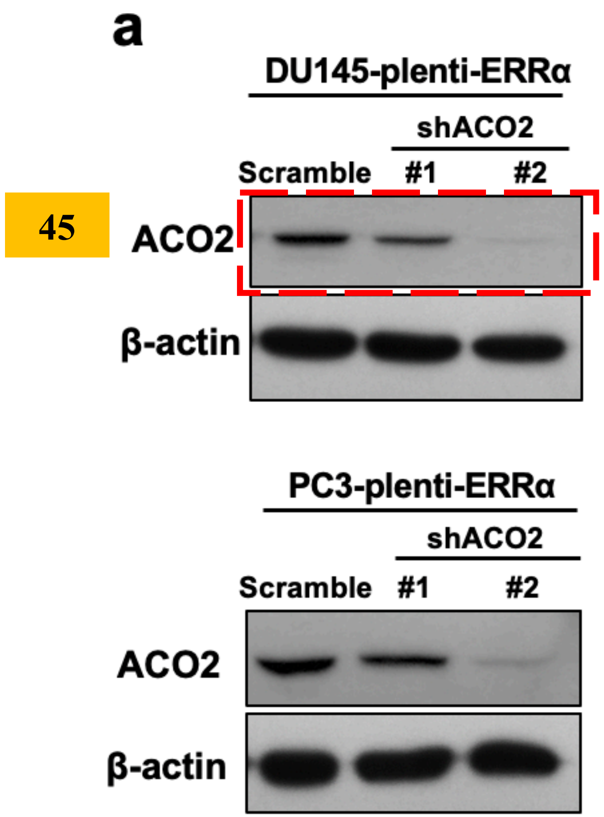
45
a
DU145-plenti-ERRα
shACO2
Scramble
#1
#2
ACO2
β-actin
PC3-plenti-ERRα
shACO2
Scramble
#1
#2
ACO2
β-actin

Fig. 6a

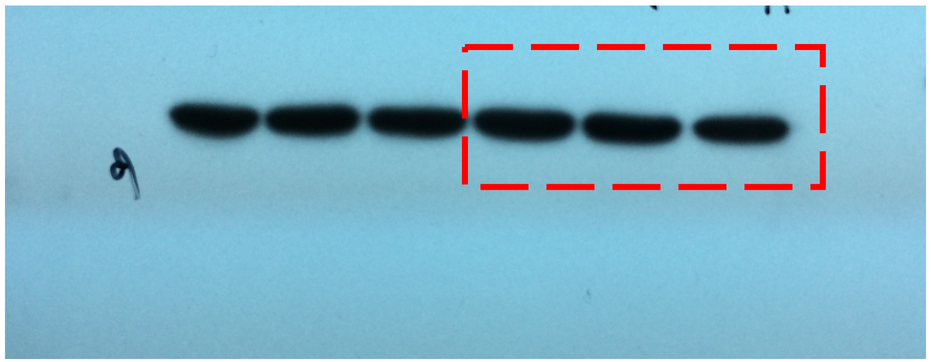

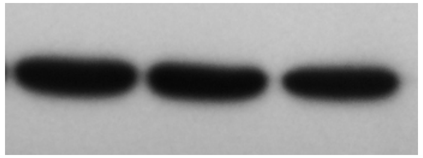

46

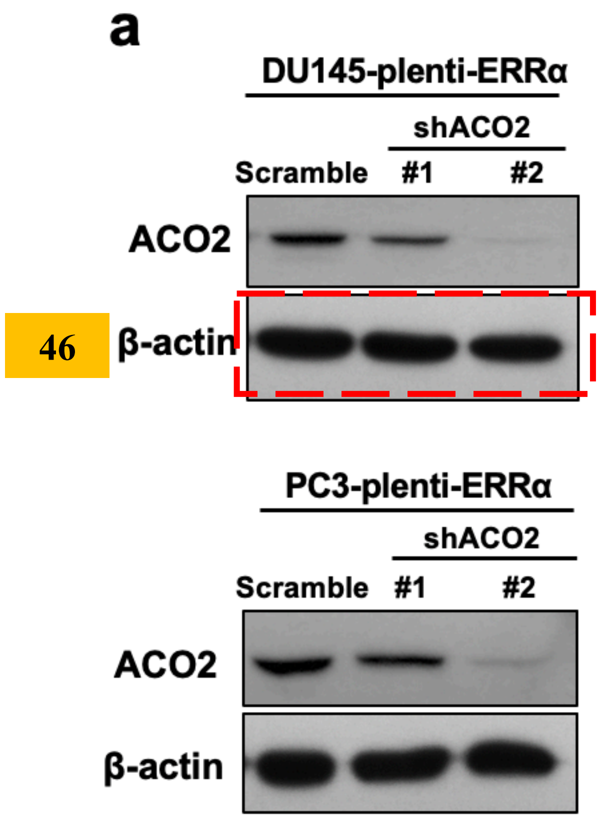
a
DU145-plenti-ERRα
shACO2
Scramble
#1
#2
ACO2
β-actin
PC3-plenti-ERRα
shACO2
Scramble
#1
#2
ACO2
β-actin

Fig. 6a

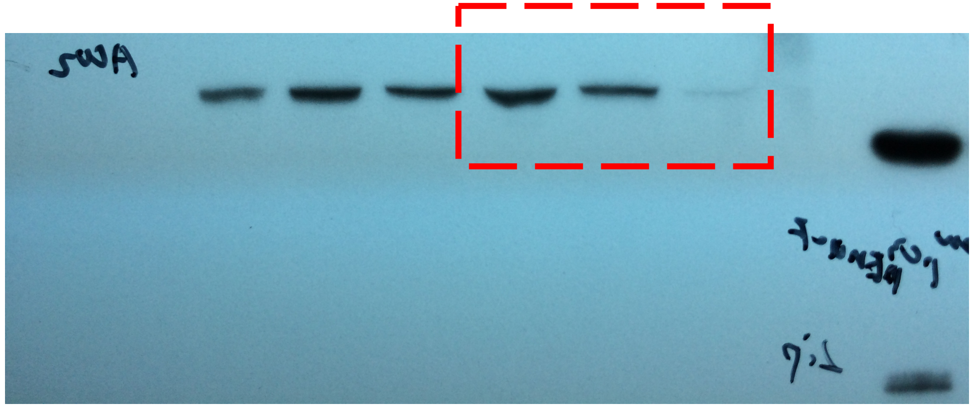

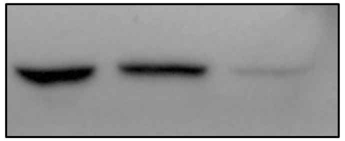

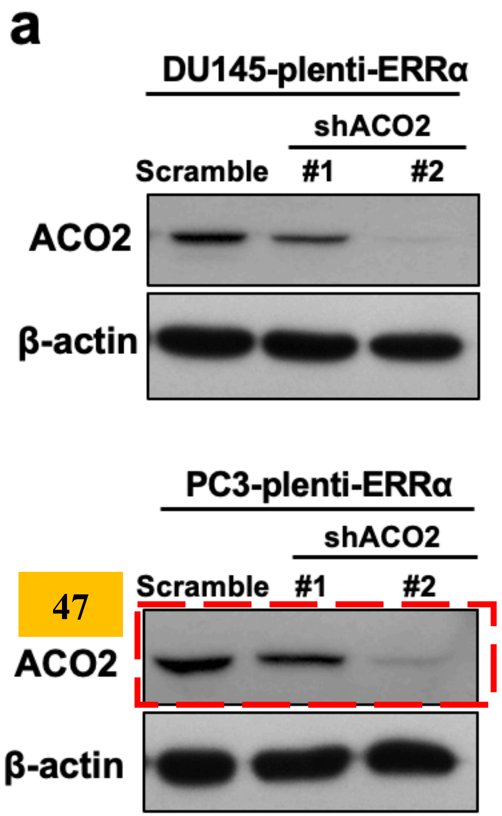
a
DU145-plenti-ERRα
shACO2
Scramble
#1
#2
ACO2
β-actin
PC3-plenti-ERRα
shACO2
47
Scramble
#1
#2
ACO2
β-actin

Fig. 6a

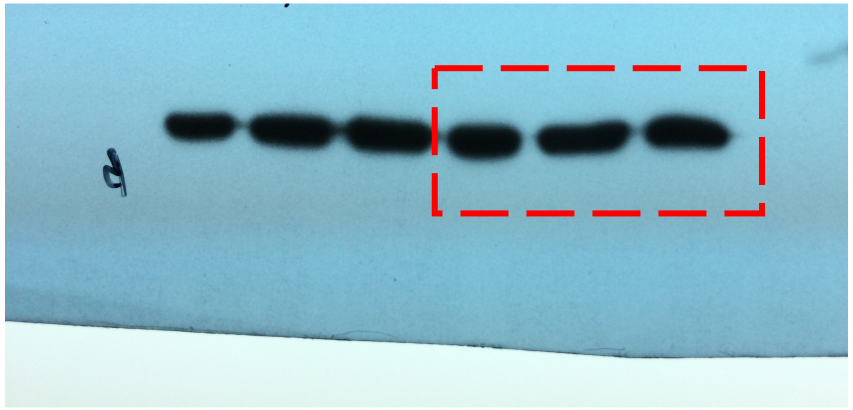

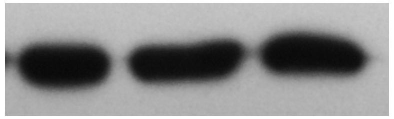

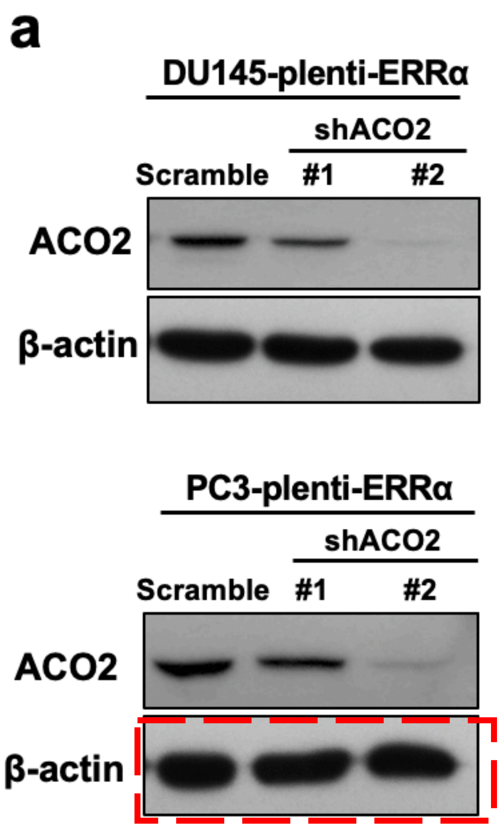
a
DU145-plenti-ERRα
shACO2
Scramble
#1
#2
ACO2
β-actin
PC3-plenti-ERRα
shACO2
Scramble
#1
#2
ACO2
β-actin

48

Fig. 6a

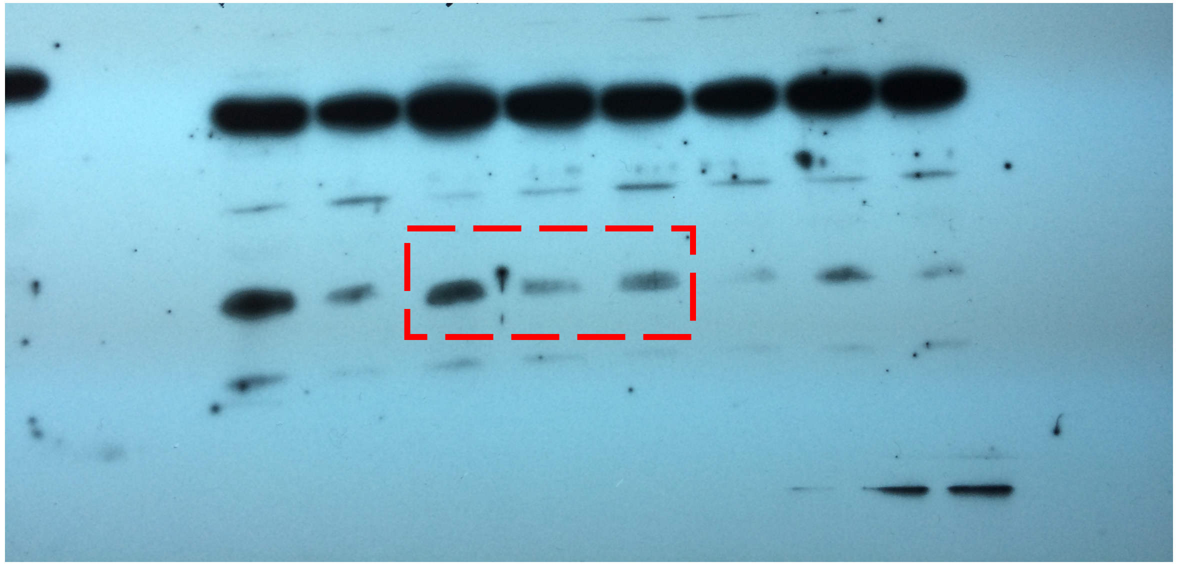

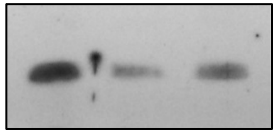

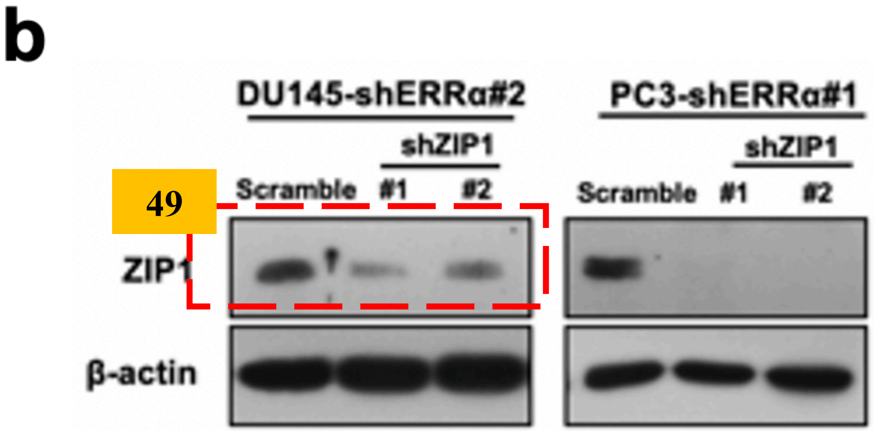
b
DU145-shERRα#2
PC3-shERRα#1
shZIP1
shZIP1
49
Scramble
#1
#2
Scramble
#1
#2
ZIP1
β-actin

Fig. 8b

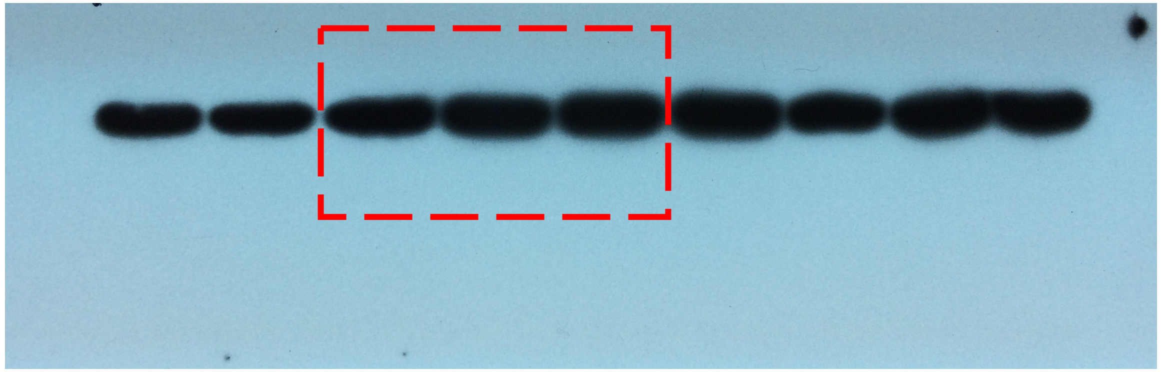

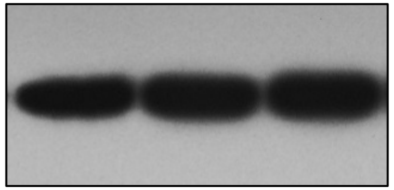

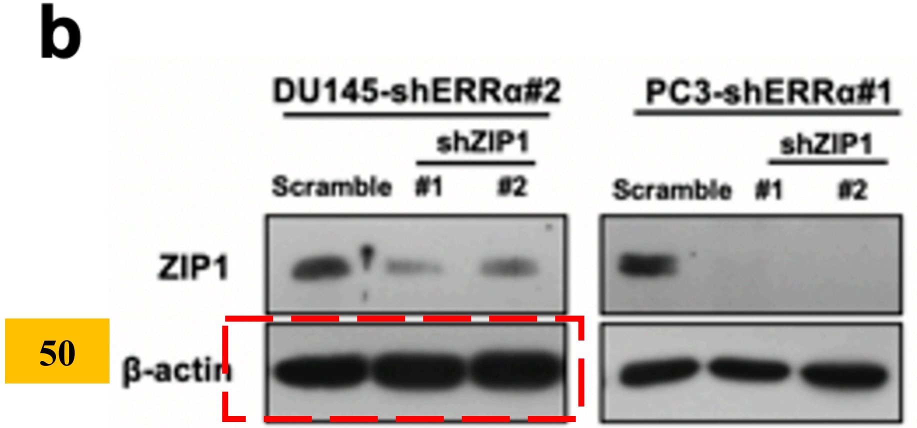
b
DU145-shERRα#2
PC3-shERRα#1
shZIP1
shZIP1
Scramble #1 #2
Scramble #1 #2
ZIP1
β-actin

Fig. 8b

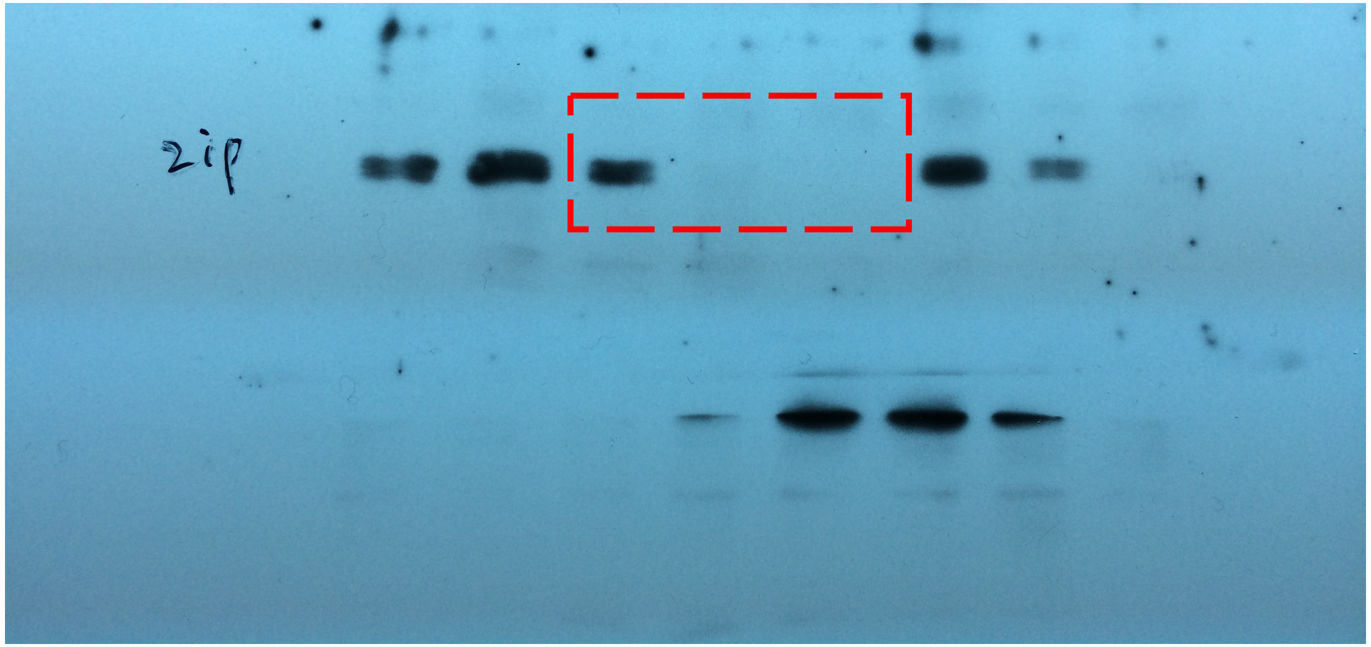
zip

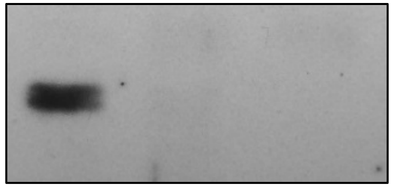

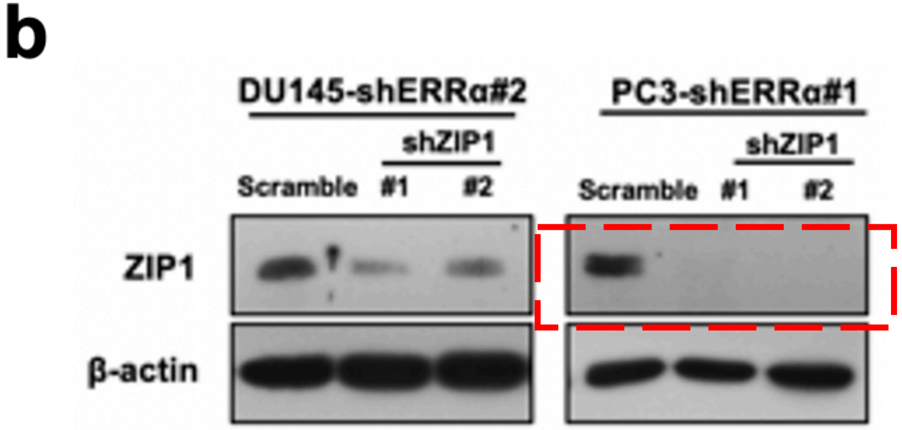
b
DU145-shERRα#2
PC3-shERRα#1
shZIP1
shZIP1
Scramble
#1
#2
Scramble
#1
#2
ZIP1
β-actin

51

Fig. 8b

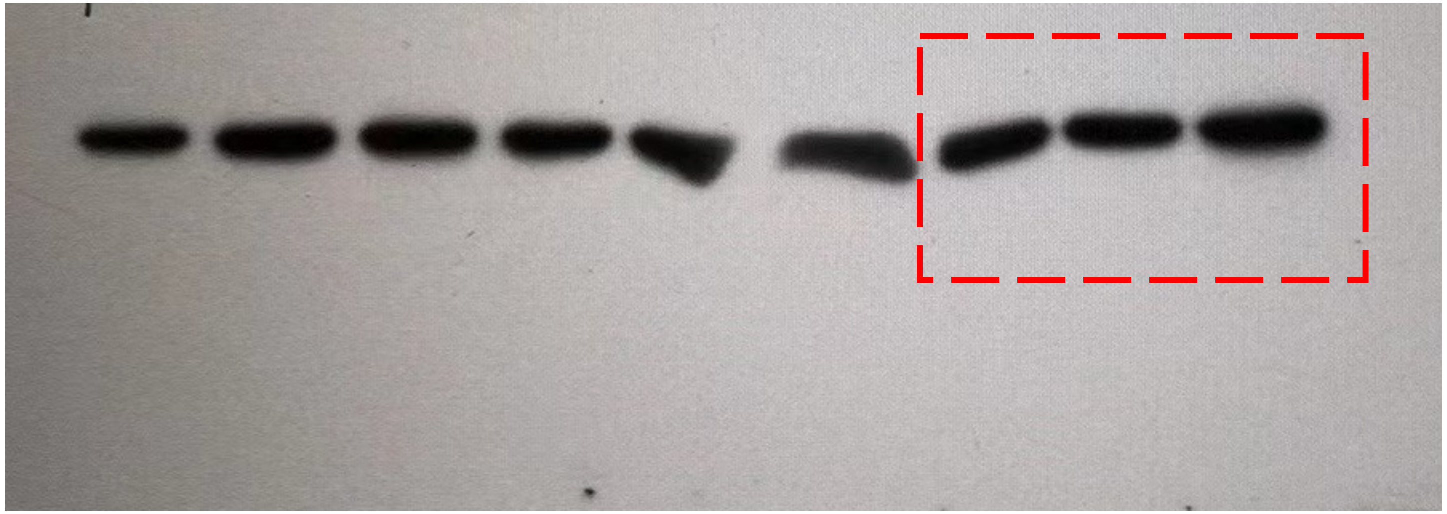

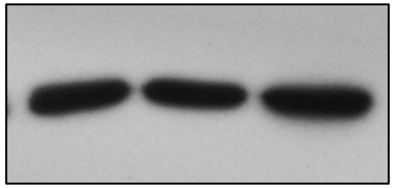

b

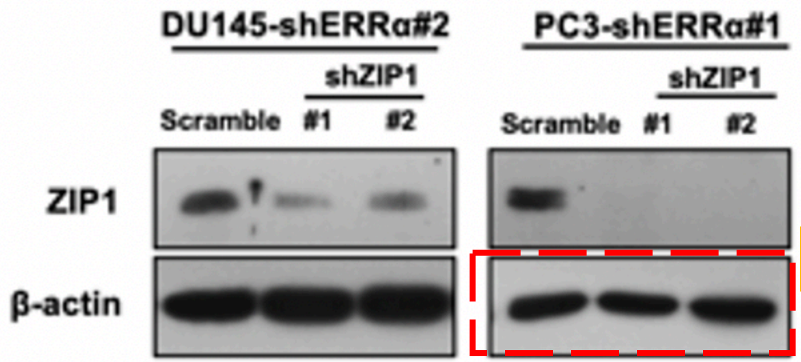
DU145-shERRα#2
PC3-shERRα#1
shZIP1
shZIP1
Scramble
#1
#2
Scramble
#1
#2
ZIP1
β-actin

Fig. 8b

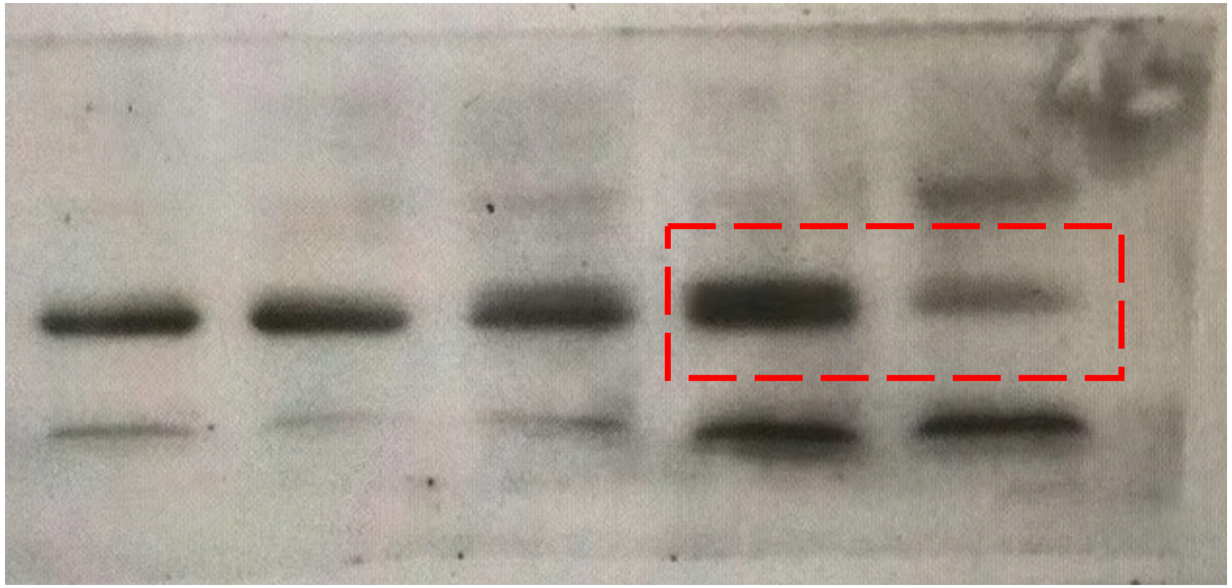

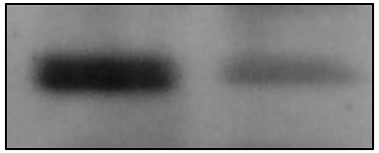

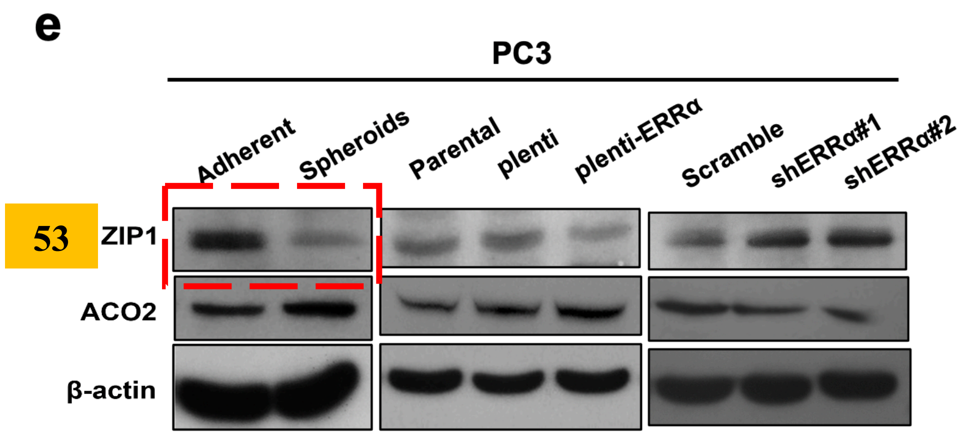
e
PC3
Adherent
Spheroids
Parental
plenti
plenti-ERRα
Scramble
shERRα#1
shERRα#2
53
ZIP1
ACO2
β-actin

Fig. S2e

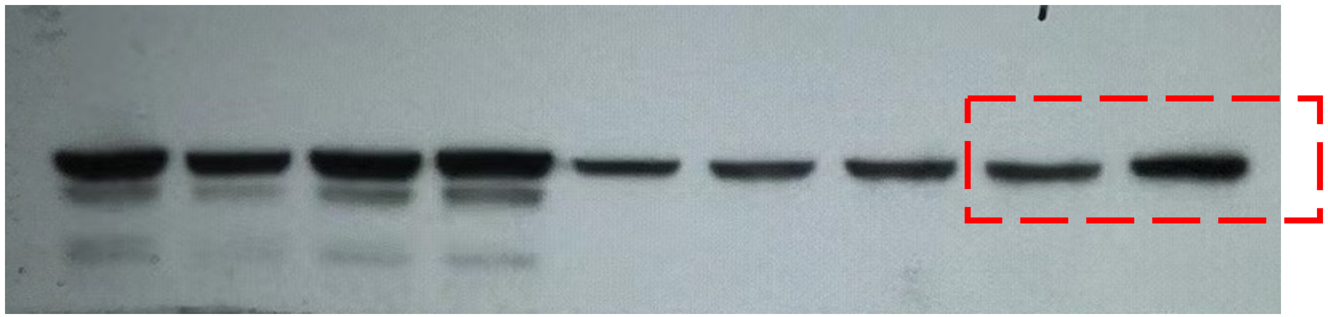

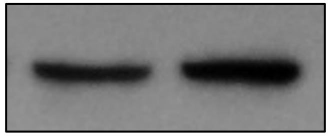

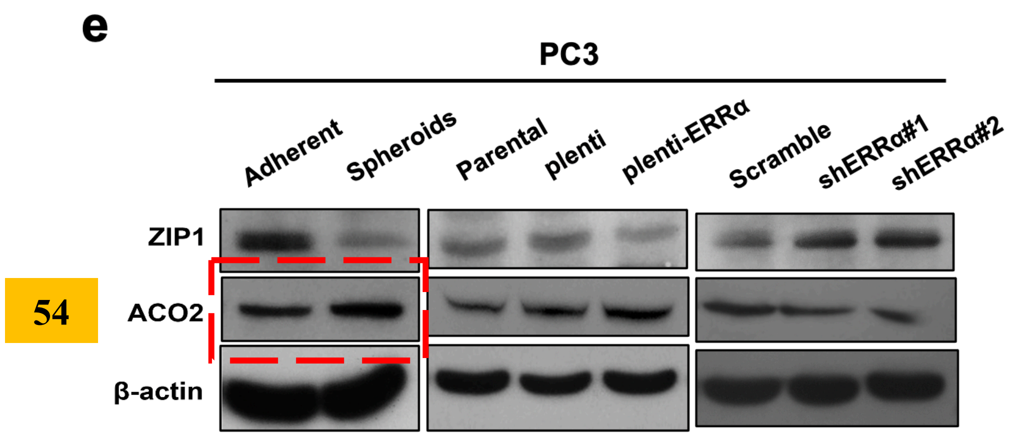
e
PC3
Adherent
Spheroids
Parental
plenti
plenti-ERRα
Scramble
shERRα#1
shERRα#2
ZIP1
ACO2
β-actin
54

Fig. S2e

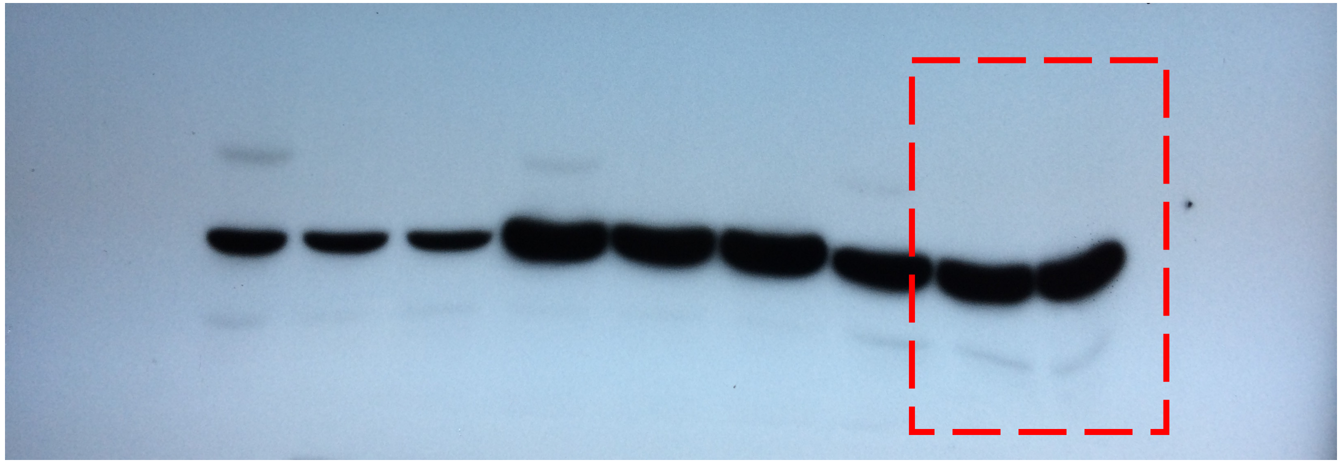

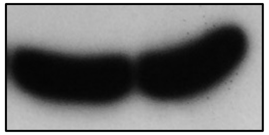

55

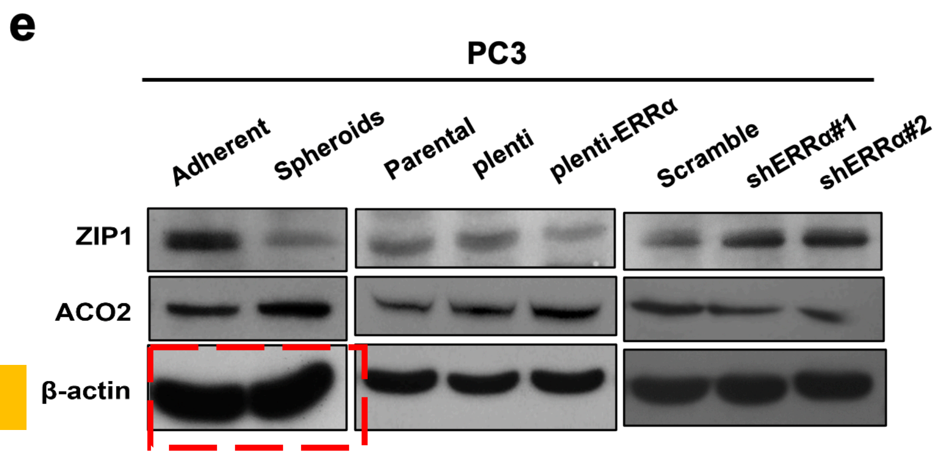

e
PC3
Adherent
Spheroids
Parental
plenti
plenti-ERRα
Scramble
shERRα#1
shERRα#2
ZIP1
ACO2
β-actin

Fig. S2e

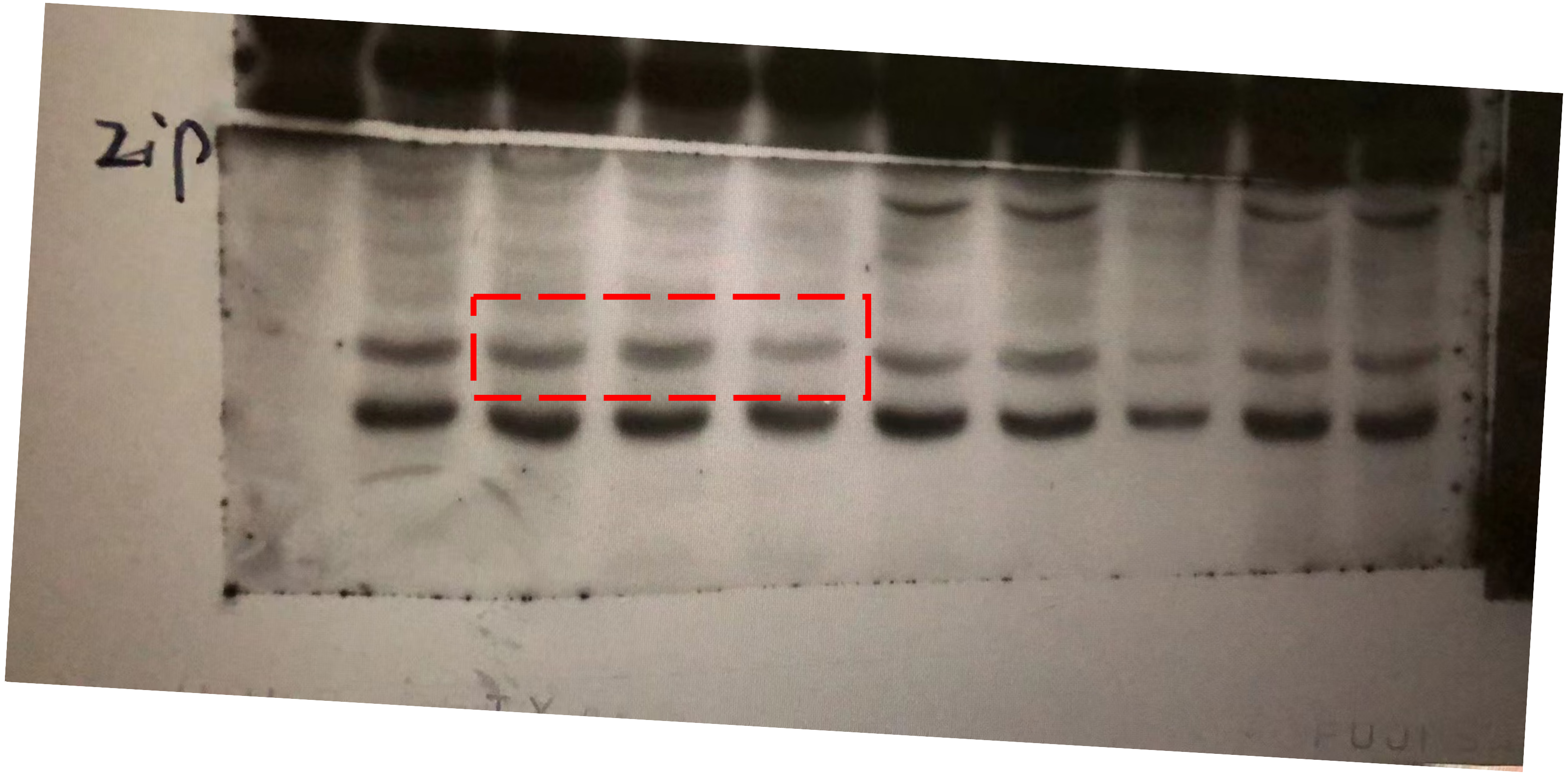
zip

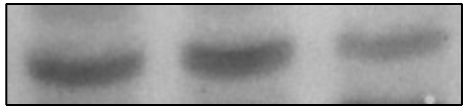

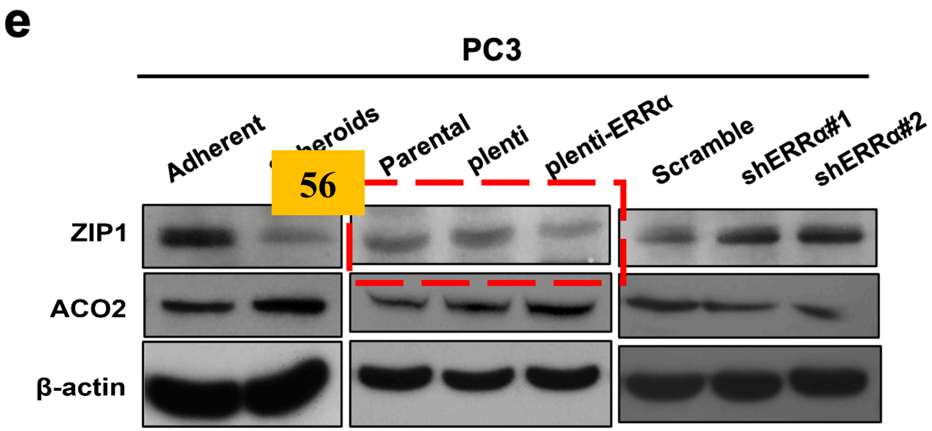
e
PC3
Adherent
eroids
Parental
plenti
plenti-ERRα
Scramble
shERRα#1
shERRα#2
56
ZIP1
ACO2
β-actin

Fig. S2e

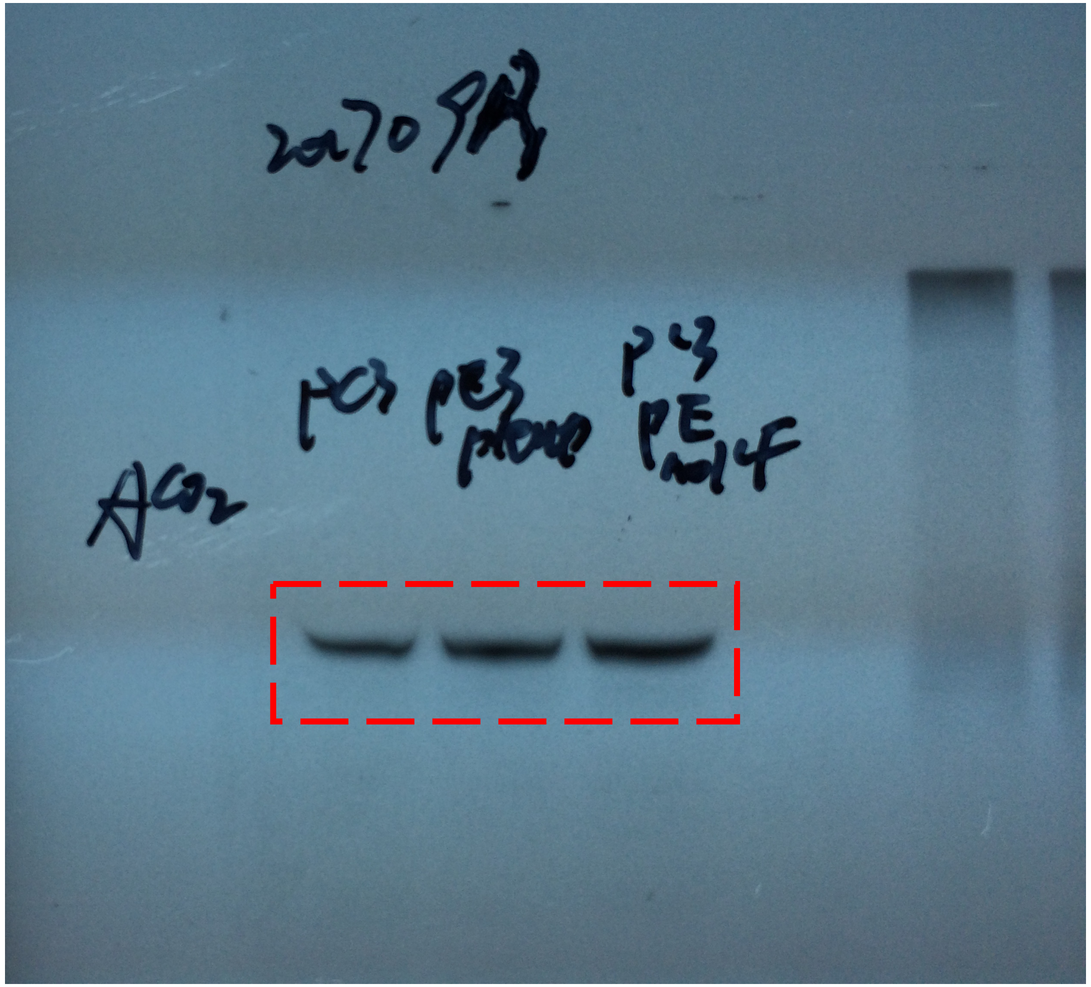

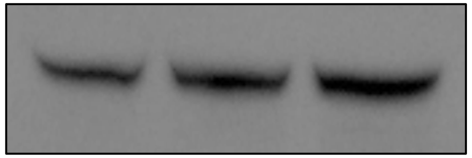

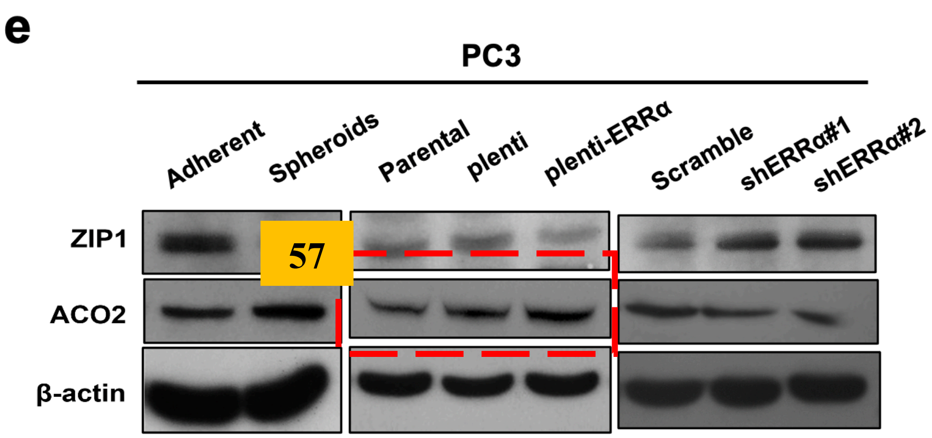

e
PC3
Adherent
Spheroids
Parental
plenti
plenti-ERRα
Scramble
shERRα#1
shERRα#2
ZIP1
57
ACO2
β-actin

Fig. S2e

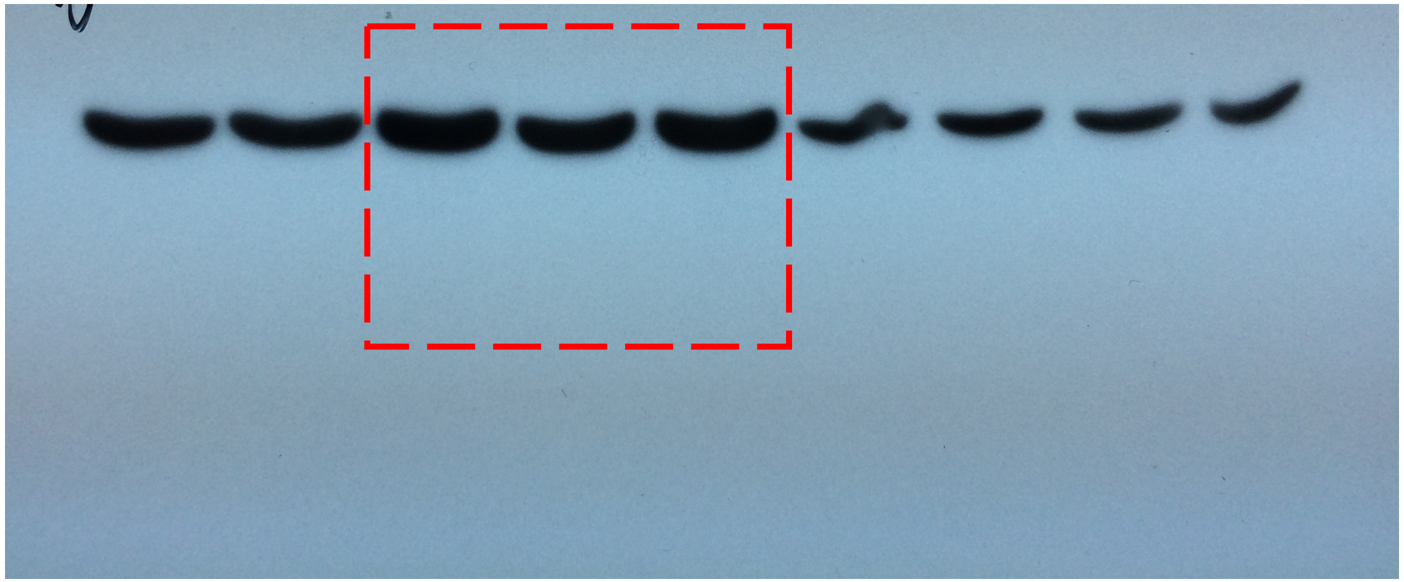

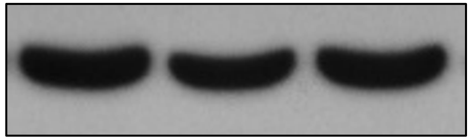

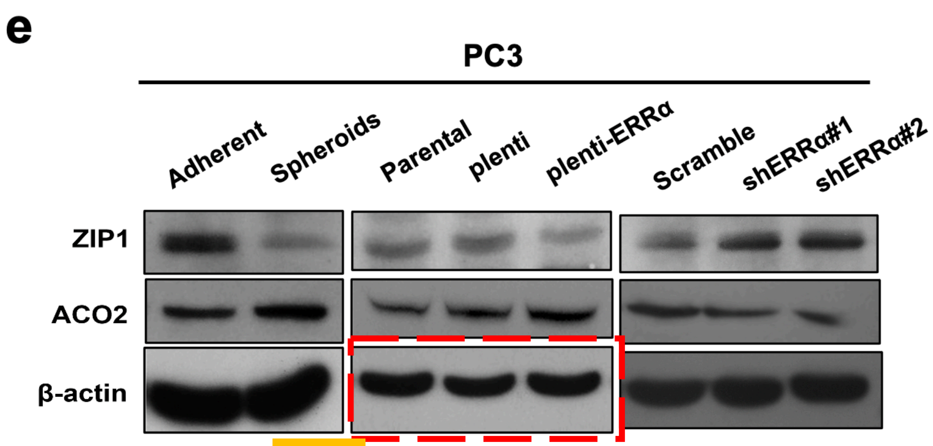
e
PC3
Adherent
Spheroids
Parental
plenti
plenti-ERRα
Scramble
shERRα#1
shERRα#2
ZIP1
ACO2
β-actin

Fig. S2e

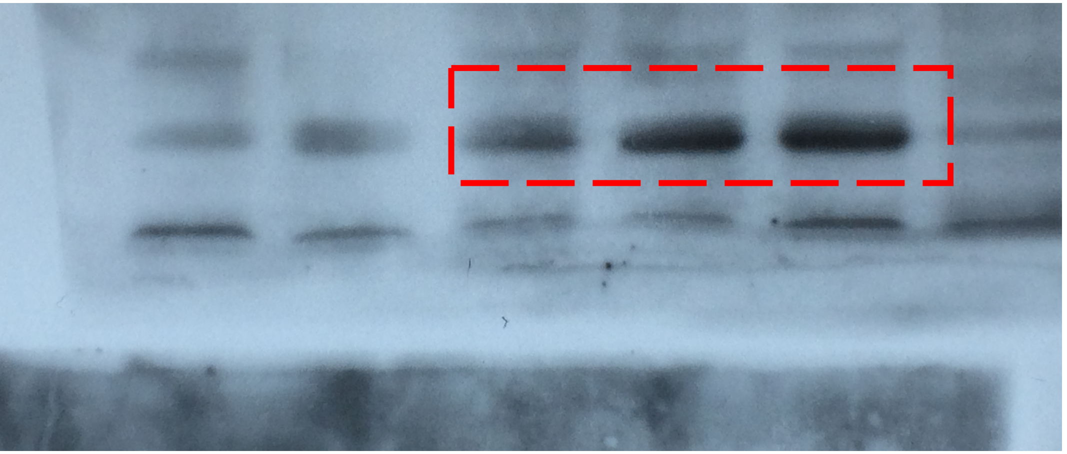

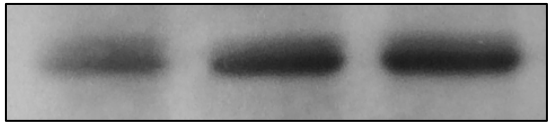

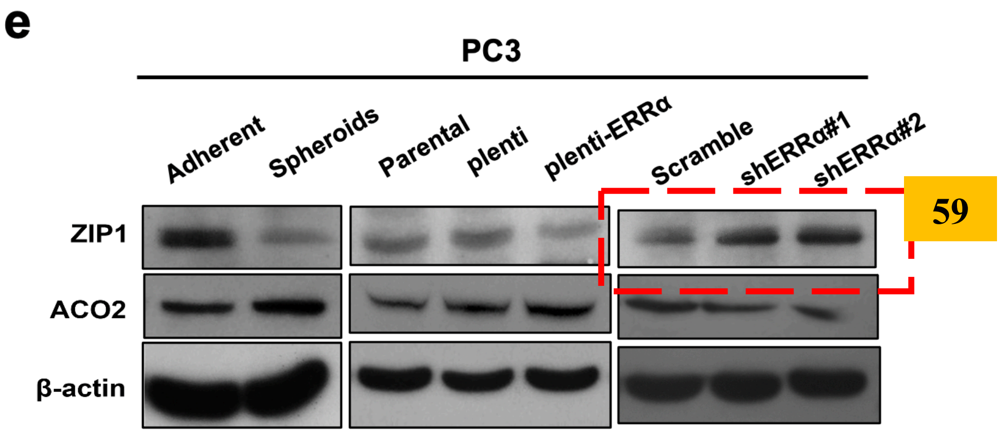
e
PC3
Adherent
Spheroids
Parental
plenti
plenti-ERRα
Scramble
shERRα#1
shERRα#2
ZIP1
ACO2
β-actin
59

Fig. S2e

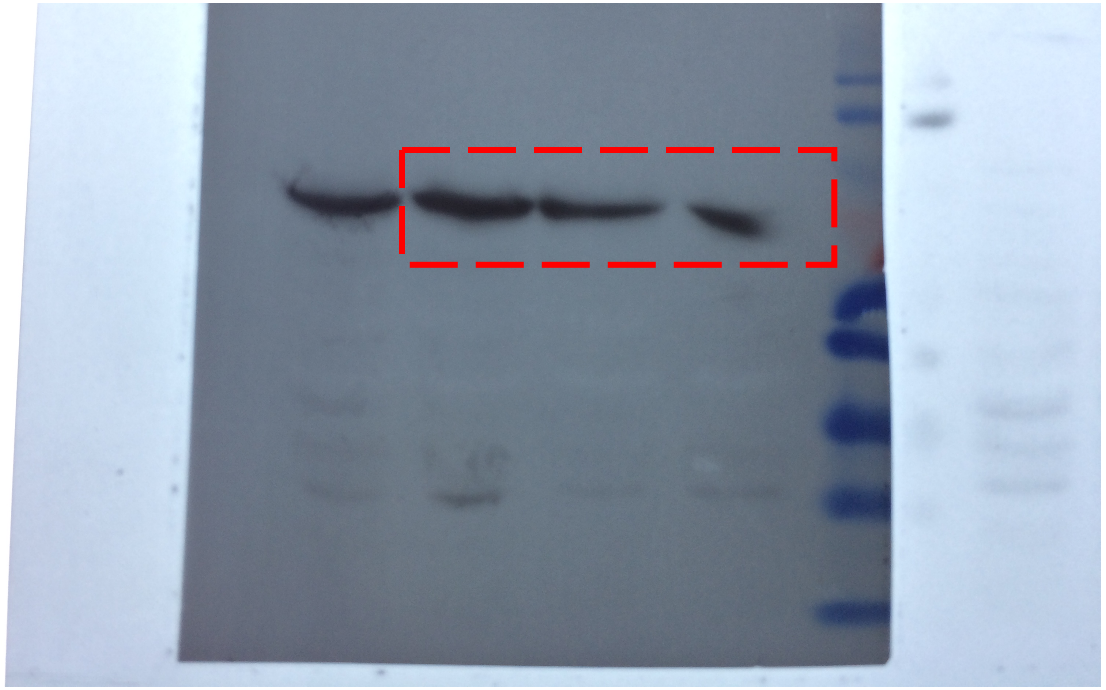

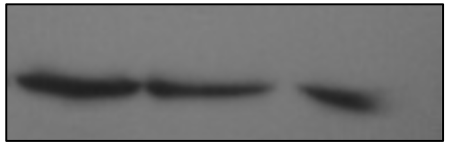

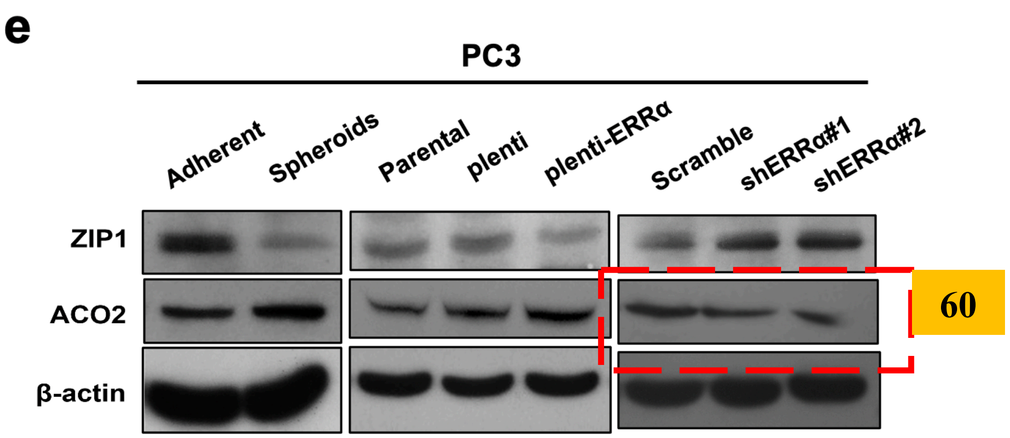
e
PC3
Adherent
Spheroids
Parental
plenti
plenti-ERRα
Scramble
shERRα#1
shERRα#2
ZIP1
ACO2
β-actin
60

Fig. S2e

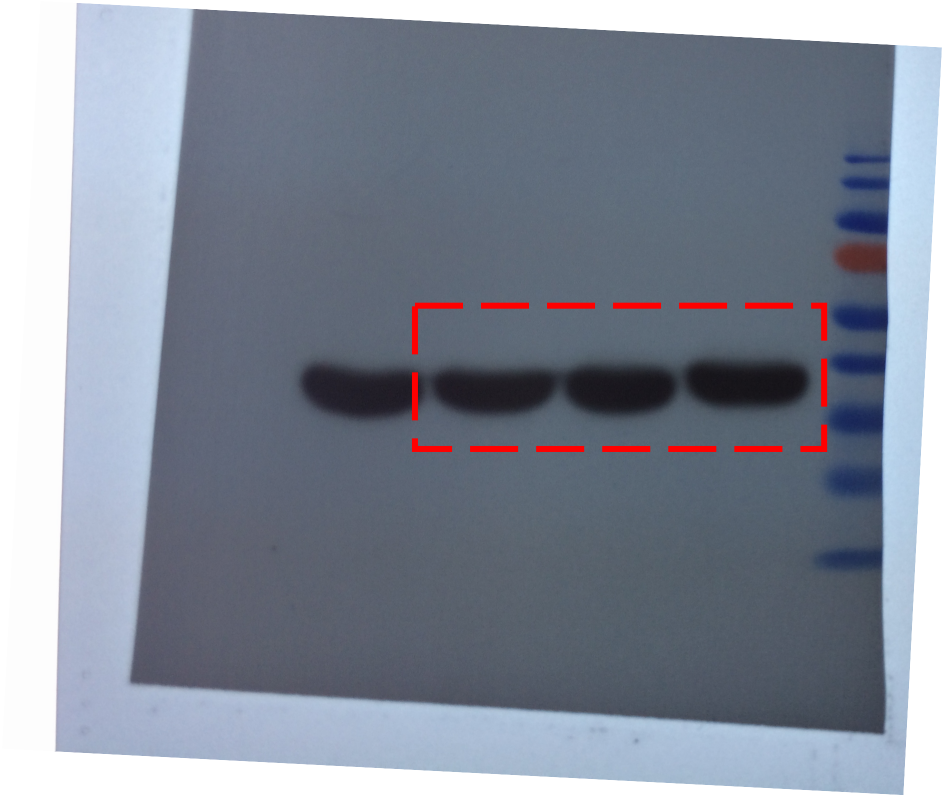

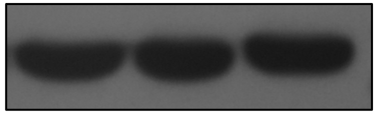

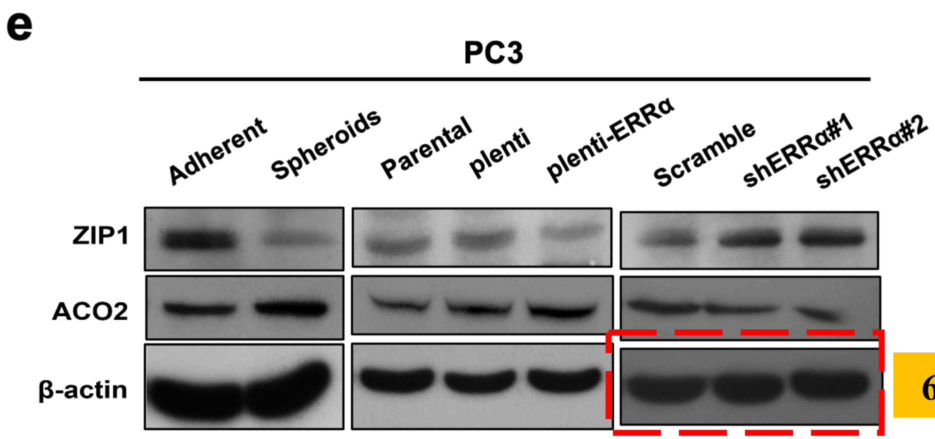
e
PC3
Adherent
Spheroids
Parental
plenti
plenti-ERRα
Scramble
shERRα#1
shERRα#2
ZIP1
ACO2
β-actin

61

Fig. S2e
